# Prioritization of livestock diseases by pastoralists in Oloitoktok Sub County, Kajiado County, Kenya

**DOI:** 10.1371/journal.pone.0287456

**Published:** 2023-07-12

**Authors:** Caroline M. Mburu, Salome Bukachi, Hamilton Majiwa, Dismas Ongore, Matthew Baylis, Kennedy Mochabo, Eric Fevre, Olivia Howland

**Affiliations:** 1 Department of Social Anthropology, University of St Andrews, St Andrews, Scotland, United Kingdom; 2 Institute of Anthropology, Gender and African Studies, University of Nairobi, Nairobi, Kenya; 3 School of Public Health, University of Nairobi, Nairobi, Kenya; 4 Institute of Infection, Veterinary and Ecological Sciences, University of Liverpool, Liverpool, United Kingdom; 5 Faculty of Veterinary Medicine and Surgery, Egerton University, Nakuru, Kenya; 6 Institute of Infection and Global Health, University of Liverpool, Liverpool, United Kingdom; University of Zambia, ZAMBIA

## Abstract

**Introduction:**

Livestock diseases are a big challenge for the livelihood of pastoralists in sub-Saharan Africa because they reduce livestock productivity and increase mortality. Based on the literature available there is limited understanding on how pastoralists prioritize these diseases in the context of their culture, ecosystems and livelihoods. A study was conducted to provide insights on lay prioritization of animal diseases by pastoralists in Kenya.

**Methodology:**

A qualitative study was undertaken between March and July 2021. Thirty in-depth interviews and six focus group discussions (FGDs) were conducted with community members to explore community attitudes on livestock diseases prioritization. Male and female livestock keepers were purposively selected and interviewed and they were all long-term residents of the area. Fourteen key informant interviews (KIIs) were conducted with professionals from different key sectors to provide detailed stakeholder perspectives on livestock diseases. The interviews were analyzed thematically using the QSR Nvivo software to identify the emerging themes related to the study objectives.

**Results:**

The pastoralists prioritized livestock diseases based on effect on their economic wellbeing, cultural values and utilization of ecosystem services. There were gender variabilities in how diseases were prioritized among the pastoralists. Men cited high priority diseases as foot and mouth disease and contagious bovine pleuropneumonia due to their regular occurrence and effect on livelihood. Notably, women regarded coenuruses as very important because it affected sheep and goats with a high mortality rate and lumpy skin disease because it rendered the meat from the carcasses inedible. Malignant catarrhal fever and trypanosomiasis were noted as some of the common diseases in the livestock-wildlife interface but not cited as priority diseases. Challenges related to disease control in pastoralist contexts exist including limited access to livestock treatment services, inadequate information on disease impact and complex environmental factors.

**Conclusion:**

This study sheds light on the body of knowledge in Kenya regarding livestock diseases and their prioritization by livestock keepers. This could aid in the development of a common disease control framework and prioritization at the local level which would take into consideration the dynamic socio-cultural, ecological, livelihood and economic contexts of the communities.

## Introduction

Pastoralism is an important mode of livelihood in the marginal lands of sub-Saharan Africa that are unsuitable for agricultural production and therefore ought to be promoted [1]. Pastoral livelihoods provide income and food for up to 300 million people in sub-Saharan Africa and have socio-cultural importance [2, 3]. Since pastoralists are dependent on livestock as a source of livelihood, losses in livestock perpetuate poverty and poor outcomes [4, 5]. Livestock keepers also continue to face marginalization, alienation and weak animal and human health infrastructure [1, 6, 7]. With increasing expansion of farm land by individuals and big businesses, pastoralist communities continue to be driven out of their traditional areas and into more remote, disease infested lands with minimal access to pasture, water and veterinary services [1, 7].

The Maasai are nomadic pastoralist communities living in the southern parts of Kenya. Studies have shown that their livelihoods are threatened by drought, diseases, environmental degradation and overgrazing [5, 8]. Changes have occurred that affect the Maasai pastoralists including changing land ownership, from communal to privately owned, which affects livestock mobility as well as overpopulation, drought and more frequent disease outbreaks [9]. Partly due to this, there is more interaction between livestock and wildlife which also promotes disease transmission and co-infections are common in livestock [5]. Therefore, disease control measures need to be very dynamic and context specific catering to the different dynamics in the socio ecological landscape of the pastoralists [10]. It has been documented that livestock keeping communities have a good understanding of animals and animal diseases by observing the behavior of the sick animal and are aware of the ecological aspects of diseases [11–13]. They draw on this experience in treating sick animals using both traditional and conventional modes of treatment [2, 14]. Maasai pastoralists often self-medicate their livestock without consulting professionals for the correct medication and dosage because they consider themselves experts due to their long standing experience as livestock keepers [12, 15]. This misuse may be contributing to the development of antimicrobial resistance [15]. To improve this situation, researchers have called for collaborations between professionals and pastoralists, including the allocation of sufficient resources to strengthen disease control mechanisms [15, 16].

It is well known that prioritization of diseases helps in the effective allocation of resources by focusing on the major challenges and this is best done by considering local contexts and including lay stakeholders [17, 18]. However, this is often done at the national level with little involvement from the communities who witness these problems first hand [19–23]. Qualitative studies do elicit context specific information that could aid in the development of locally acceptable and relevant solutions. In rural Tanzania, brucellosis symptoms in livestock were not perceived as major issues of concern and other diseases like foot-and-mouth disease were more prioritized by agro pastoralists [24]. In Cameroon, animals that were known to be chronically sick were retained in the herds to avoid selling them at a low price [25]. In a qualitative study conducted in Georgia on peste des petits ruminants (PPR), it was observed that the pastoralists did not mention this disease as a priority problem [26]. The single disease focus of many research agencies often does not resonate with local communities because lay people conceptualize their challenges holistically [7, 26]. This study therefore was conducted to understand how pastoralists in Oloitoktok sub county in Kajiado County prioritize animal diseases within their socio ecological context.

## Methods

### Design and setting

This study was part of the One Health Network for the Horn of Africa (HORN project).

### Study area

**Fig 1:**
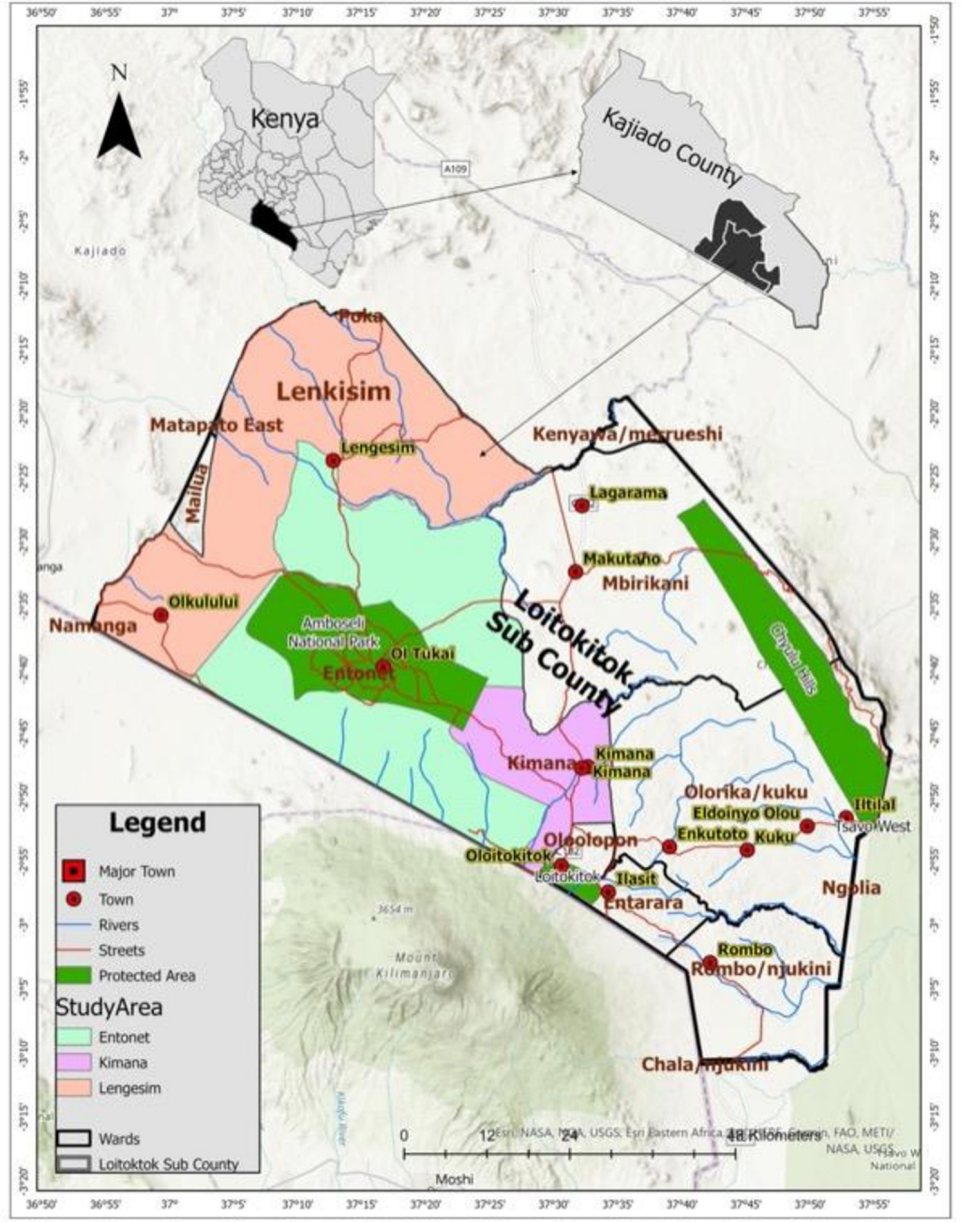
Map of the study area.

The study area was Oloitoktok sub county in Kajiado County which has an estimated population of 191,846 [27]. This study was conducted in selected villages in three wards in the Sub county namely Kimana, Entonet and Lengisim (Fig 1). The wards selected for this study were purposively chosen according to geographical and contextual considerations. Entonet Ward is close to the Amboseli national park and was selected in order to understand how proximity and interaction with wildlife influenced community perceptions on livestock diseases. Lengisim ward, which borders Tanzania, was chosen in order to conceptualize how movement across the border might influence people’s perceptions on livestock diseases. Kimana ward, which is a peri urban site, was selected in order to understand any possible differences in attitudes between rural and more urban pastoralists on disease prioritization.

### Study population

We used purposive sampling to recruit the study participants for the in depth (IDI) and key informant interviews (KII) as well as the focus group discussions (FDGs). We recruited IDI participants by using community elders who identified 10 households in each ward (N = 30) where we interviewed one male and one female in each household. Community leaders also helped us to recruit participants for the FGDs who were different from those who had been involved in the IDIs. The participants were aged between 18–75 years. All the participants were Maasai and residents of the area. For KIIs, we approached the stakeholders directly and requested for the interviews with the help of the subcounty veterinary officer. These were livestock extension officers, clinician, public health officer, wildlife officer and the veterinary officer and community elders (male). They all were working in the subcounty for a period ranging from a few months to five years. The participants all gave written consent to be interviewed.

### Data collection methods

We used in depth interviews (IDIs) with 30 community members to obtain information on their motivation for livestock keeping, animal grazing and movement patterns, common livestock diseases and prioritization of those diseases. Each in depth interview was conducted in the home of the participant and the interviews were conducted in Swahili or in the Maa language with the help of research assistants fluent in both languages. A male interviewer interviewed the male participants and a female interviewer the female participants. An interview guide (S1 Data) was used and each interview lasted 20–60 minutes and all the interviews were audio recorded with the consent of the participants. All the participants were over 18 years of age.

On completion of the IDIs, we conducted 2 focus group discussions in each site (n-6) one each with men and women. The topics that were covered in these discussions were knowledge of livestock diseases, perceived risk factors for livestock diseases, perceived severity, impact and prioritization of these diseases. The participants for the FGDs were those who had not been interviewed using IDIs and they were purposively selected in order to gather knowledgeable and diverse participants. Each FGD had 7–12 people and the male and female researchers moderated the discussions with men and women respectively for between 60–90 minutes. These discussions were conducted in Swahili or the Maa language with the help of research assistants. Participants ranged in age from 20–75 years old. The discussions were conducted in locations accessible to all participants and they were each given 5 USD as transport reimbursement. All the discussions were recorded with the permission of the participants. KIIs were conducted alongside the IDIs and FGDs. These were done to gain insights on common livestock diseases in the area in humans and livestock and aspects of livestock-wildlife interactions. We also explored the local names given to livestock diseases. Lastly, a mapping exercise with community elders was done to understand how the local community utilized their spaces focusing on homes, watering points and grazing areas and we sought information on how these areas were used during the dry and wet seasons.

### Data management and analysis

We recorded all the interviews and also took notes. The research assistants who are fluent in Maa, Swahili and English translated all the interviews conducted in the Maa language. We used the notes taken as data and used them to validate the transcripts. Two of the authors (CM and HM) conducted data analysis using the NVIVO 10 software (QSR International, Australia) for the development of codes. The coding process entailed the two authors categorizing the data to group concepts that related to each other noting the similarities and differences between them. Thereafter the two authors met and agreed on the codes and harmonized them. Later, these codes were grouped into themes according to the study objectives and translated verbatim. Quotes have been used to exemplify the main points of this study.

### Ethics

Ethical approval for this study was sought and obtained from the International Livestock Research Institute review board in Nairobi and the University of Liverpool Central University Research Ethics Committee. Permission to conduct this research was also acquired from the National Commission for Science, Technology and Innovation and the County government of Kajiado.

## Results

### Demographics

This study was conducted between March- July 2021. We conducted 30 in-depth interviews, 15 key informant interviews and 6 focus group discussions with a total of 108 participants in three wards Kimana, Lengisim and Entonet wards in Oloitoktok sub county as illustrated in Table 1 below.

**Table 1:** A summary of the socio-demographic characteristics of the study participants.

Study area	Methods of data collection	Number and gender of participants	Occupation
Kimana ward	FGD	10 Male 11 Female	Livestock keepers
	IDI	5 Male 5 Female	Livestock keepers
Lengisim ward	FGD	9 Male 10 Female	Livestock keepers
	IDI	5 Male 5 Female	Livestock keepers
Entonet ward	FGD	11 Male 12 Female	Livestock keepers
	IDI	5 Male 5 Female	Livestock keepers
Oloitoktok Sub County	KII	3 (2 Male, 1 Female) 1 Male 1 Male 1 Male 2 Male 1 Male 6 Male	Livestock Officers Veterinary Officer Medical Officer Wildlife Officer Clinicians Public Health Officer Village Elders

## Results

The prioritization of animal diseases by the pastoralists was embedded in their socio cultural, economic and ecological context. Important diseases were identified by the livestock keepers based on their impact on livelihoods and some gender differences in prioritization were identified. These aspects are discussed below under three themes which are cultural values and livelihood strategies, lay perceptions of livestock diseases and access to ecosystem services.

### Cultural values and livelihood strategies

Livestock in this community were valued for livelihood and as a cultural practice. The benefits of livestock that were cited included being a source of food for meat, milk and fat as well as a means of income to cater for various family needs. Cattle were especially preferred by men because they provided a higher income when sold. In an in-depth interview with one pastoralist, he opined that: “*Cattle are very important to me; Personally*, *I have not gone to school and if one is not educated you cannot find employment and even if you get employed it will be a very low paying job*. *We therefore focus on our animals that can help us because you can sell and take your child to school*, *you can get milk and that is why cows are very important to us”*. *(IDI Male Entonet Ward)*. Women particularly valued sheep as a source of meat and fat for lactating mothers and sick people as demonstrated in the quotes below:

*“They help me in life because when I give birth*, *I eat meat and I also keep livestock for milk and so I must always have the livestock*”.

*IDI Female Kimana Ward*

*“When sick we slaughter a sheep and consume the fat*, *we also sell these animals and use the money to take someone who is sick to the hospital*, *when there is no food*, *we milk and drink the milk*. *We also use the cream from the milk*.

*IDI Female Lengisim Ward*

Consequently, men prioritized commonly occurring diseases including foot and mouth disease (FMD) and contagious bovine pleuropneumonia (CCPP) because these led to mortality of large numbers of cattle and goats thus affecting their livelihoods significantly. Men in one FGD concluded that, *“Foot and mouth disease is the greatest priority because it affects many animals and is common in the area*. *The second one is contagious caprine pleuropneumonia which affects the goats in masses*. *These two diseases cause many animals to die”*. *(FGD Men Kimana ward)*. This is similar to how professionals in the area prioritized livestock diseases focusing on foot and mouth disease due to its regular occurrence. *“Although diseases like contagious bovine pleuropneumonia and lumpy skin disease are common in this area the main disease of importance is foot-and-mouth disease as it occurs very frequently*. *We vaccinate for this disease but not routinely due to lack of sufficient resources”*.

*Veterinary Officer*, *Male*, *Oloitoktok Sub county*

Women mentioned the lumpy skin disease as the priority disease because the carcass was unfit for human consumption and coenuruses which was always fatal. The loss of milk and meat for women due to disease caused them to rank rarer but fatal diseases as a big priority.

*“The biggest challenge is coenuruses because it has no cure*. *Lumpy skin disease is the second one as it causes losses to the owners since the cow has to be buried because it cannot be consumed*”.

*FGD Women Entonet ward*

*“Lumpy skin disease is way worse because you don’t consume the meat or the milk and you have to throw the animal away*. *Coenuruses is also a big problem because the animals never respond to treatment and always die and it has become very common*.

*FGD Women Lengisin ward*

### Lay perceptions of livestock diseases

The community understanding of livestock diseases also influenced how they prioritized them. The livestock keepers determined that their livestock were diseased based on physical and observable signs mainly rough hair coat, wounds, salivating, watery eyes, running nose, loss of appetite, shivering, lethargy and low milk production. The participants used the word “*isuuro*” to refer to the general condition of dullness, rough hair coat, self-isolation, droopy ears and not feeding. *“An animal is like our child*. *When sick the animal changes*, *the hair coat will be rough and appear different*. *You will notice that it has fed poorly so you can tell that it is sick and will ask the herdsman if the animal was feeding well that day”*. *(IDI Male*, *Kimana ward)*. Livestock officers observed that the pastoralists had lived alongside animals for many years and therefore were generally good at identifying sick animals.

*“Most of these pastoralists have lived with these animals for a long time so they know how to identify a sick animal and even tell you the disease the animal is suffering from*. *They identify disease by checking the behavioral changes in the animal because when an animal is sick it does not feed or water*, *it lags behind and has a rough hair coat and in some cases like when it has FMD the animal limps*”.

*Veterinary officer*, *Oloitoktok subcounty*.

The pastoralists had extensive knowledge on the diseases that afflicted cattle, goats and sheep including the signs and symptoms as shown in Table 2. They also identified the possible causes, prevention strategies and methods of treatment. Professionals in the area corroborated this by saying that the livestock keepers had good understanding of local livestock diseases. The pastoralists described these diseases in Maa and we obtained the English equivalents after the study from veterinary and animal health professionals who spoke Maa and worked in the area.

**Table 2:** Pastoralists perceptions and prioritization of livestock diseases.

Disease *(In Maa)*	Disease *(in English)*	Livestock affected	Characteristics of the disease	Seasonal aspects	Prevention and treatment in livestock
*Olorobi*	Foot and mouth disease	Cattle, goats and sheep	Excessive salivation, running nose, wounds on the hooves, lethargy and not feeding. Low milk production. Highly contagious.	Present all the time but especially during the rainy season.	It is treated using *teramycine* (tetracycline).
*Olegipei*	Contagious Caprine Pleuropneumonia (CCPP)	Goats	Excessive coughing, affects the lungs, kills many goats, diarrhea, labored breathing, weight loss due to poor feeding. Highly contagious.	All the time	A vaccine exists to prevent the disease and penicillin for treatment.
*Oltikana*	East Coast Fever (ECF)	Cattle	Diarrhea, bloody urine, weakness, joint swelling, yellow meat once the animal is slaughtered, excessive salivation, emaciation, animal struggles to breath, sometimes the meat from the carcass cannot be eaten due to foul smell. No milk let down.	Common during and after the rains.	Not identified
*Enariri*	Lumpy Skin Disease (LSD)	Cattle, sheep and goats.	Sores on the hair coat and doesn’t feed. The animal develops a foul smell too. Very contagious and often fatal.	Common during the rainy season.	It is treated using *teramycine* (tetracycline).
*Engeya Enarogua*	Enterotoxaemia	Sheep and goats.	It is a disease with sudden onset and animals die within a very short period. The animal develops blood clots which are noticed when the animal is slaughtered. Affects healthy animals, the animal just dies and didn’t appear ill.	Common during the rainy season.	Not identified.
*Enguruya Ol Chang’et /* *Engeya Oluguny’*	Malignant Catarrhal Fever	Cattle	Blindness, Rough hair coat, weak joints, dry mouth and makes a wailing sound. It affects the head and has no cure. Animal is restless and keeps running. Shakes the head and starts circling. Tail falls off.	Occurs after the wildebeests have given birth and then the grass grows and the livestock feed on that grass.	No cure
*Olmillo*	Coenurosis	Goats and sheep	The animal gets worms in the head and keeps circling. Affects the spine too, attacks sheep and goats but mostly goats. Emaciation because it doesn’t feed, it doesn’t die fast, there is circling and intense bellowing. It runs off so they have to tie it. Happens all the time, it is a new disease but becoming more and more common. They slaughter for meat but they don’t eat the head meat because it has worms and fluids. There is diarrhea too. It can be sick for a long time. Touching the ears makes the animal wince in pain.	Common all the time.	They use tetracycline, cutting off one ear, feeding it sugar water but most of the time all these don’t work and it eventually dies.
*Nunuk*	Bovine ephemeral fever (Three-day disease)	Cattle	There is excessive salivation, shivering, hard stool and no milk let down. Rarely kills the animal.	During drought	They pour ash on the back of the animal and leave it in the sun for a while. This they do to warm up the animal as it shivers. When the cow urinates then they know that the medicine has worked.
*Eng’ororo/Eng’oroto*	Nagana/trypanosomiasis	Cattle	The animal becomes malnourished, dry mouth, hard black dung, swelling of the front limbs, blood clots which one notices after the animal dies and is slaughtered. No milk let down too and emaciation. Highly fatal.	Always there but especially when animals move to the mountains.	They use the medicine Veriben to treat the animal.
*Enadogulak/Enadomonyet/Enadogurum*	Bloody diarrhoea	Cattle, sheep and goats	Diarrhea happens when the animal eats soil instead of salts. When slaughtered the intestines are swollen and filled with gas.	Not identified	For the calves they apply kerosene on the anus or burn the tail with a hot iron. Usually recover with these strategies and tetracycline.
*Nkoleny*	Not identified	Cattle and goats	Caused by ticks and makes the cow very sleepy and not come home so sometimes they are eaten by wild animals. Causes paralysis. Goat kids are the most affected. The hind limbs are weak and the animal cannot walk. It has no cure. It attacks the joints of the animals. It is transmitted from wild animals.	Not identified	Not identified
*Olmonko*	Not identified	Cattle	Wounds that ooze blood on the animal’s body. It is transmitted from wildlife	Not identified	They apply cow dung on the wound. The cow dung is a covering to prevent the birds from licking the wound. They pour salt and other antibiotic capsules on the wound. They also use tetracycline
*Olodua*	Peste des petits ruminants	Cattle, sheep and goats	The liver enlarges and becomes watery and yellowish. Blackish diarrhea. Kidneys enlarge and then they burst. It may come suddenly and you identify the disease after the animal has died and you slaughter it.	Not identified	Only the liver is affected and once one dies the farmer vaccinates the rest of the herd.

They recognized that cattle dips, deworming and vaccination were useful in preventing animal diseases. Other known strategies were quarantining sick animals although they noted that this was not routinely practiced. The livestock keepers used both traditional and conventional methods to treat their sick animals. Penicillin and oxytetracycline were the most used drugs for the majority of the diseases because they were believed to be very effective. Diseases which were believed to be cured by locally available drugs were considered less of a priority including trypanosomiasis. Traditional methods were used for very specific diseases including bovine ephemeral fever (three day disease) and bloody diarrhea which were regarded as fairly easy to treat. *“There is a disease called nunuk (three-day disease)*. *When an animal is sick with this disease*, *we apply ash on its body and leave it out in the sun and in most cases*, *it recovers*. *For other diseases we use oxytetracycline*, *we don’t like to call the doctors because with some of the medicines they use if the animal dies the carcass cannot be consumed”*.

*IDI Female Entonet ward*.

*“We use mainly oxytetracycline and penicillin and the good thing with penicillin is that it cures many diseases*. *Even for contagious caprine pleuropneumonia which is very dangerous the penicillin helps*. *We have lived with livestock for a long time so we know what to do*”.

*KII Elder Kimana Ward*.

Animal health professionals agreed that pastoralists hardly ever sought their advice and preferred to treat animals themselves often through trial and error.

*“They just use guesswork because they come to the market and buy oxytetracycline*, *penicillin and dewormers*. *For dewormers*, *I can say they are doing better because they understand the dosage and the right cycles to deworm*. *We have taught them the dosages by estimating the weight of the animals*. *For treatment however*, *they don’t differentiate between oxytetracycline and penicillin*. *They can inject the former in the morning and the latter in the evening*. *So*, *there is drug interaction and the animal dies in some cases*.”

*KII Female livestock health officer*, *Oloitoktok*.

### Theme 3: Access to ecosystem services

The pastoralists were aware that drought and diseases were increasing in intensity with lack of reliable water sources and pasture. Although, they knew that their socio ecological interactions were likely to lead to disease in livestock due to comingling of herds and livestock-wildlife interactions they perpetuated this behavior to access crucial ecosystem services including pasture and water. In an effort to search for grazing lands, the pastoralists went further into areas with wildlife causing their animals to be preyed upon by lions, leopards and hyenas. The community also knew that this close proximity between wildlife and livestock led to disease transmission across the species. As a result of drought, livestock congregated in specific places to graze and this was perceived as a leading cause of increased disease transmission. A key informant noted that, *“Drought can be severe and we lack water and so we have to take animals to the Chyulu hills and that is far*. *It is about 27 kms from here and the animals have to come back here for water*. *As a result*, *the animals lose weight*, *become emaciated and are more susceptible to disease especially because in those areas there are many ticks which cause diseases*.*”*.

*Male*, *Elder*, *Entonet ward*.

Disease causation was attributed to the movement and grazing patterns of the livestock as well as seasonal changes. The community moved their livestock in search of pasture and water to various places including to the Chyulu hills, Mombasa, Amboseli, Maasai Mara and sometimes to Tanzania. The extent of this movement depended on how severe the drought was, which was often between July to October. The pastoralists perceived disease transmission to occur as animals including wildlife congregated in one place. The pastoralists had to trade off this risk for the benefit of pasture and water in this ecosystem. Although, they understood diseases like malignant catarrhal fever and trypanosomiasis to occur from these interactions, these were not mentioned as important diseases. *“We know that wild animals transmit diseases such as malignant catarrhal fever (MCF) because of the contamination of the pasture with the afterbirth from wildebeests giving birth*. *Trypanosomiasis is also caused by some insects which are found in the Chyulu hills*”.

*IDI Female Lengisin ward*.

Community leaders also noted that in the past the management of pasture lands was well coordinated by the community. This ensured that enough pasture was available throughout the seasons, land degradation was minimal and there were fewer livestock-wildlife interactions. This was not the case anymore due to political interference and changing societal values. This had led to increased human-wildlife conflicts and environmental degradation. The lack of proper disease control programs was also a big challenge and this was corroborated by livestock officers as in the excerpt below:

*“Pastoralists in this area face a lot of challenges*. *The first challenge is drought*. *Pastoralists keep migrating from this area towards Chyulu hills and Amboseli*. *Secondly there is the problem of conflicts with wild animals which prey on their livestock and lack of proper disease control*”.

*KII Livestock health assistant*, *Oloitoktok Sub county*.

Local leaders observed that the movement of livestock was supposed to be regulated through permits to prevent the transmission of disease but this was not implemented. They also corroborated the findings that disease transmission did indeed occur more during the drought season due to increased animal movement and interactions.

*“During the drought season many diseases spread when animals go to common areas looking for pasture and water*. *This is the time where diseases like foot and-mouth*, *lumpy skin and contagious caprine pleuropneumonia (CBPP) among others are very common*. *Animals will migrate to a common area such as Chyulu hills in search of pasture and this is the time they interact and spread diseases to each other*.”

*KII Animal Health officer*, *Oloitoktok county*.

## Discussion

It is imperative to understand how local communities understand and conceptualize diseases as this is integral in developing contextually suitable and effective ways of mitigation especially in synchronizing local and professional perspectives. In order for disease control efforts to be sustainable in pastoralist settings culture, livelihoods and ecology need to be considered [28]. Pastoralists are well aware of livestock diseases and appropriate control strategies in their context and thus should be included in disease control strategies [17, 29]. This study focused on determining the prioritization of animal diseases by pastoralists in Oloitoktok subcounty in Kenya. We wanted to understand how the pastoralists determined diseases of importance as rooted in their socio ecological context. This study found that livestock production played a big role in sustaining the livelihoods of the pastoralists by providing food and disposable income. This demonstrates that their livelihoods were greatly affected when animals suffered and died from diseases and this could lead to poverty because of lack of resilience. Concurringly, studies in various parts of Africa have shown that livestock play a significant role in the livelihoods of many pastoralists in sub Saharan Africa [2, 5]. There were gender differences in diseases which were considered important by the livestock keepers. Men prioritized diseases with a high mortality and affecting cattle such as FMD while women ranked as important lumpy skin disease because the meat from the carcass was inedible. Women regarded coenuruses as a priority disease because it afflicted sheep and goats and had a 100% mortality rate. In many pastoralist settings in Kenya, women own small ruminants and thus losses in these species could be more acutely felt by women and disenfranchise them [30] In Ethiopia women regarded lumpy skin disease to be more important than anthrax which was important to men [31]. Others have observed that pastoralists livelihoods are increasingly being compromised by disease outbreaks and changes in land tenure which affects mobility and thus access to pasture and water [1, 7, 9].

In this particular study, the pastoralists regularly used the term “*isuuro”* to describe a diseased animal and this meant the animal had a starey coat, was not feeding and appeared dull. This grouping of symptoms into one category could lead to misdiagnosis and erroneous treatment of animals. [11]. In Ethiopia, similar to this study’s findings pastoralists identified and classified disease syndromes based on the part of the body that was affected or the major clinical sign [32]. Pastoralists struggle to identify diseases that do not have distinct clinical symptoms [11]. Others though have said that caution needs to be exercised when applying local syndromes to known diseases because there can be local variations such as several syndromic terms at different stages of disease [32, 33]. They reported treating animals using antibiotics which they used liberally and without formal diagnosis. In this study, both conventional and traditional methods were used to treat the animals but they reported to mainly use penicillin and tetracycline to treat their livestock. Diseases which were perceived to be easy to treat such as trypanosomiasis, bloody diarrhea and bovine ephemeral fever were low priority diseases. Other pastoralists in Kenya have also been found to use vaccination, antibiotics and local treatment practices to control diseases in livestock [2]. The livestock keepers primary concern often is that the medicine they administere to their animals is effective and that is why they purchase veterinary medicines [34]. Livestock keepers in this study were knowledgeable on many livestock diseases. In their study in Tanzania, the Maasai named animal diseases by symptoms, body part affected and the ecology of the disease [11]. Self-treatment of livestock is common among pastoralists in many parts of Africa [2, 15, 24]. Studies have demonstrated that there is misuse of antibiotics among livestock keepers in Africa and that this could fuel the development of antimicrobial resistance [12, 35]. Limited access to veterinary services, reliance on friends and past experiences in determining treatment and easy access of drugs in the villages have been identified as some of the reasons why pastoralists self-medicate their livestock [36, 37].

The livestock keepers were well aware that grazing patterns, animal movements and livestock-wildlife interactions contributed to disease transmission but continued to engage in these activities to access pasture and water for their animals. Designing disease control strategies is complex in pastoralist settings because of the uncontrolled livestock mobility and sustained livestock wildlife interactions [5, 10, 38]. In their study in northern Kenya, pneumonia, Peste des Petits ruminants (PPR), contagious caprine pleuropneumonia (CCPP), tick borne diseases and diarrhea were some of the common diseases identified by the pastoralists in livestock [2]. Pastoralists in Uganda knew that close proximity and interaction with wildlife was a risk factor for disease [39, 40]. However, this knowledge did not impact on practice because they had to balance the risk of disease with access to crucial ecosystem services like pasture and water [5]. The Maasai have vast knowledge on livestock diseases and they base their diagnosis on the season, vector and species affected. This study findings corroborates others which have noted that foot-and-mouth disease, tick borne diseases, PPR and CCPP are some of the common livestock diseases in pastoral areas [2].

In our study, the diseases that were prioritized by the pastoralists were those that occurred very often like FMD and CBPP and those that could not be cured such as coenuruses or that rendered the carcass unusable. Pastoralists care about how diseases affect their entire livelihood [34]. Livestock keepers in Uganda and Ethiopia prioritized diseases based on fatality rates, incidence, market value, reduced production and associated treatment costs [32, 41]. In their study in Kajiado County in Kenya, the diseases which were considered to have a significant impact were CBPP, FMD and lumpy skin disease [42]. In other places such as in Denmark researchers have found that peoples experience with a disease in terms of severity and fatality and not frequency of occurrence determines perceptions of priority of disease [43]. Informal assessments of risk are crucial and they are determined by the context and how people experience that disease [44].

## Conclusion

The pastoralists allocate importance of disease based on their knowledge, impact on livelihood and socio ecological factors. This study has demonstrated how livestock diseases are perceived and prioritized by the Maasai and their prioritization of the diseases might be different from veterinarians’. This understanding can inform interventions which are beneficial to the communities in a socially, culturally and ecologically sensitive and appropriate manner. This is because for any control efforts to be fruitful, the communities need to be involved. The involvement of communities is essential to the efficacy of livestock health interventions. Others have shown that it is important to use qualitative research to obtain information on the local context to understand how people in that context understand disease risk [32, 40]. This study recommends that disease control programs in this locality should incorporate lay perspectives to be accepted, helpful and sustainable. Educating the pastoralists on the use and misuse of antibiotics is important and extension workers could incorporate indigenous knowledge in community sensitization and engagement.

### Limitations

This study utilized qualitative data which means that the results of this study cannot be generalized. Nevertheless, it provides rich insights which can be used in further studies and in the development of culturally and ecologically relevant disease control approaches. Majority of the participants responded in the Maa language and thus some insights could have been lost during translation. This though was mitigated through the use of research assistants who were fluent in the Maa, Swahili and English languages.

## Supporting information

S1 Data (ZIP)

## References

[1] Ripkey C, Little PD, Dominguez-Salas P, Kinabo J, Mwanri A, Girard AW. Increased climate variability and sedentarization in Tanzania: Health and nutrition implications on pastoral communities of Mvomero and Handeni districts, Tanzania. Glob Food Secur. 2021 Jun;29:100516.

[2] Abdilatif MH, Onono JO, Mutua FK. Analysis of pastoralists’ perception on challenges and opportunities for sheep and goat production in Northern Kenya. Trop Anim Health Prod. 2018 Oct;50(7):1701–10. doi: 10.1007/s11250-018-1613-8 29770944

[3] Desta AH. Pastoralism and the Issue of Zoonoses in Ethiopia. J Biol. 2016;7.

[4] Howland O, Noe C, Brockington D. The multiple meanings of prosperity and poverty: a cross-site comparison from Tanzania. J Peasant Stud. 2021 Jan 2;48(1):180–200.

[5] Nthiwa D, Alonso S, Odongo D, Kenya E, Bett B. A participatory epidemiological study of major cattle diseases amongst Maasai pastoralists living in wildlife-livestock interfaces in Maasai Mara, Kenya. Trop Anim Health Prod. 2019 Jun;51(5):1097–103. doi: 10.1007/s11250-018-01790-1 30684224 PMC6520318

[6] Chenais E, Fischer K. Increasing the Local Relevance of Epidemiological Research: Situated Knowledge of Cattle Disease Among Basongora Pastoralists in Uganda. Front Vet Sci. 2018 Jun 7;5:119. doi: 10.3389/fvets.2018.00119 29951490 PMC6008553

[7] Quinn CH, Huby M, Kiwasila H, Lovett JC. Local perceptions of risk to livelihood in semi-arid Tanzania. J Environ Manage. 2003 Jun;68(2):111–9. doi: 10.1016/s0301-4797(03)00013-6 12781751

[8] Ameso E, Bukachi S, Olungah C, Haller T, Wandibba S, Nangendo S. Pastoral Resilience among the Maasai Pastoralists of Laikipia County, Kenya. Land. 2018 Jun 19;7(2):78.

[9] Kaoga J, Olago D, Ouma G, Ouma G, Onono J. The evolving cultural values and their implications on the Maasai Pastoralists, Kajiado County, Kenya. Sci Afr. 2021 Sep;13:e00881.

[10] Omondi GP, Obanda V, VanderWaal K, Deen J, Travis DA. Animal movement in a pastoralist population in the Maasai Mara Ecosystem in Kenya and implications for pathogen spread and control. Prev Vet Med. 2021 Mar;188:105259. doi: 10.1016/j.prevetmed.2021.105259 33453561 PMC8787859

[11] Mangesho PE, Neselle MO, Karimuribo ED, Mlangwa JE, Queenan K, Mboera LEG, et al. Exploring local knowledge and perceptions on zoonoses among pastoralists in northern and eastern Tanzania. Budke CM, editor. PLoS Negl Trop Dis. 2017 Feb 1;11(2):e0005345. doi: 10.1371/journal.pntd.0005345 28146556 PMC5325590

[12] Mangesho PE, Caudell MA, Mwakapeje ER, Ole-Neselle M, Kimani T, Dorado-García A, et al. Knowing Is Not Enough: A Mixed-Methods Study of Antimicrobial Resistance Knowledge, Attitudes, and Practises Among Maasai Pastoralists. Front Vet Sci. 2021 Mar 22;8:645851. doi: 10.3389/fvets.2021.645851 33834048 PMC8023390

[13] Mangesho PE, Caudell MA, Mwakapeje ER, Ole-Neselle M, Kabali E, Obonyo M, et al. “We are doctors”: Drivers of animal health practices among Maasai pastoralists and implications for antimicrobial use and antimicrobial resistance. Prev Vet Med. 2021 Mar;188:105266. doi: 10.1016/j.prevetmed.2021.105266 33517159

[14] Kioko J, Baker J, Shannon A, Kiffner C. Ethnoecological knowledge of ticks and treatment of tick-borne diseases among Maasai people in Northern Tanzania. Vet World. 2015 Jun;8(6):755–62. doi: 10.14202/vetworld.2015.755-762 27065643 PMC4825278

[15] Caudell MA, Quinlan MB, Subbiah M, Call DR, Roulette CJ, Roulette JW, et al. Antimicrobial Use and Veterinary Care among Agro-Pastoralists in Northern Tanzania. Browning GF, editor. PLOS ONE. 2017 Jan 26;12(1):e0170328. doi: 10.1371/journal.pone.0170328 28125722 PMC5268417

[16] Kiplagat SK, Kitala PM, Onono JO, Beard PM, Lyons NA. Risk Factors for Outbreaks of Lumpy Skin Disease and the Economic Impact in Cattle Farms of Nakuru County, Kenya. Front Vet Sci. 2020 May 29;7:259. doi: 10.3389/fvets.2020.00259 32548130 PMC7274042

[17] Kairu-Wanyoike SW, Kiara H, Heffernan C, Kaitibie S, Gitau GK, McKeever D, et al. Control of contagious bovine pleuropneumonia: Knowledge, attitudes, perceptions and practices in Narok district of Kenya. Prev Vet Med. 2014 Aug;115(3–4):143–56. doi: 10.1016/j.prevetmed.2014.03.029 24768437 PMC4062945

[18] Mpouam SE, Mingoas JPK, Mouiche MMM, Kameni Feussom JM, Saegerman C. Critical Systematic Review of Zoonoses and Transboundary Animal Diseases’ Prioritization in Africa. Pathogens. 2021 Aug 3;10(8):976. doi: 10.3390/pathogens10080976 34451440 PMC8401284

[19] Munyua P, Bitek A, Osoro E, Pieracci EG, Muema J, Mwatondo A, et al. Prioritization of Zoonotic Diseases in Kenya, 2015. Markotter W, editor. PLOS ONE. 2016 Aug 24;11(8):e0161576. doi: 10.1371/journal.pone.0161576 27557120 PMC4996421

[20] Ng V, Sargeant JM. A Stakeholder-Informed Approach to the Identification of Criteria for the Prioritization of Zoonoses in Canada. Adler B, editor. PLoS ONE. 2012 Jan 6;7(1):e29752. doi: 10.1371/journal.pone.0029752 22238648 PMC3253104

[21] Pieracci EG, Hall AJ, Gharpure R, Haile A, Walelign E, Deressa A, et al. Prioritizing zoonotic diseases in Ethiopia using a one health approach. One Health. 2016 Dec;2:131–5. doi: 10.1016/j.onehlt.2016.09.001 28220151 PMC5315415

[22] Sekamatte M, Krishnasamy V, Bulage L, Kihembo C, Nantima N, Monje F, et al. Multisectoral prioritization of zoonotic diseases in Uganda, 2017: A One Health perspective. Kuhn JH, editor. PLOS ONE. 2018 May 1;13(5):e0196799. doi: 10.1371/journal.pone.0196799 29715287 PMC5929520

[23] Trang DT, Siembieda J, Huong NT, Hung P, Ky VD, Bandyopahyay S, et al. Prioritization of zoonotic diseases of public health significance in Vietnam. J Infect Dev Ctries. 2015 Dec 30;9(12):1315–22. doi: 10.3855/jidc.6582 26719937

[24] Mburu CM, Bukachi SA, H. Tokpa K, Fokou G, Shilabukha K, Ezekiel M, et al. Lay attitudes and misconceptions and their implications for the control of brucellosis in an agro-pastoral community in Kilombero district, Tanzania. Chaves-Olarte E, editor. PLoS Negl Trop Dis. 2021 Jun 10;15(6):e0009500. doi: 10.1371/journal.pntd.0009500 34111114 PMC8219154

[25] Healy Profitós JM, Moritz M, Garabed RB. What to do with chronically sick animals? Pastoralists’ management strategies in the far north region of Cameroon. Pastor Res Policy Pract. 2013;3(1):8. 10.1186/2041-7136-3-8 PMC4193801 25309717

[26] Chenais E, Wennström P, Kartskhia N, Fischer K, Risatti G, Chaligava T, et al. Perceptions of pastoralist problems: A participatory study on animal management, disease spectrum and animal health priorities of small ruminant pastoralists in Georgia. Prev Vet Med. 2021 Aug;193:105412. doi: 10.1016/j.prevetmed.2021.105412 34144495

[27] Kenya National Bureau of Statistics, editor. 2019 Kenya population and housing census. Nairobi: Kenya National Bureau of Statistics; 2019.

[28] Perry B, Grace D. The impacts of livestock diseases and their control on growth and development processes that are pro-poor. Philos Trans R Soc B Biol Sci. 2009 Sep 27;364(1530):2643–55. doi: 10.1098/rstb.2009.0097 19687035 PMC2865091

[29] Ebata A, Hodge C, Braam D, Waldman L, Sharp J, MacGregor H, et al. Power, participation and their problems: A consideration of power dynamics in the use of participatory epidemiology for one health and zoonoses research. Prev Vet Med. 2020 Apr;177:104940. doi: 10.1016/j.prevetmed.2020.104940 32244084

[30] Ogolla KO, Chemuliti JK, Ngutu M, Kimani WW, Anyona DN, Nyamongo IK, et al. Women’s empowerment and intra-household gender dynamics and practices around sheep and goat production in South East Kenya. Van Campenhout B, editor. PLOS ONE. 2022 Aug 4;17(8):e0269243. doi: 10.1371/journal.pone.0269243 35925935 PMC9352016

[31] Gizaw S, Desta H, Alemu B, Tegegne A, Wieland B. Importance of livestock diseases identified using participatory epidemiology in the highlands of Ethiopia. Trop Anim Health Prod. 2020 Jul;52(4):1745–57. doi: 10.1007/s11250-019-02187-4 31898026 PMC7858201

[32] Jones BA, Muhammed A, Ali ET, Homewood KM, Pfeiffer DU. Pastoralist knowledge of sheep and goat disease and implications for peste des petits ruminants virus control in the Afar Region of Ethiopia. Prev Vet Med. 2020 Jan;174:104808. doi: 10.1016/j.prevetmed.2019.104808 31710946 PMC6983938

[33] Launiala A. How much can a KAP survey tell us about people’s knowledge, attitudes and practices? Some observations from medical anthropology research on malaria in pregnancy in Malawi. 2009;11:13.

[34] Moritz M, Ewing D, Garabed R. On Not Knowing Zoonotic Diseases: Pastoralists’ Ethnoveterinary Knowledge in the Far North Region of Cameroon. Hum Organ. 2013 Jan;72(1):1–11. doi: 10.17730/humo.72.1.72672642576gw247 23990687 PMC3754808

[35] Caudell MA, Dorado-Garcia A, Eckford S, Creese C, Byarugaba DK, Afakye K, et al. Towards a bottom-up understanding of antimicrobial use and resistance on the farm: A knowledge, attitudes, and practices survey across livestock systems in five African countries. Yildirim A, editor. PLOS ONE. 2020 Jan 24;15(1):e0220274. doi: 10.1371/journal.pone.0220274 31978098 PMC6980545

[36] Iramiot JS, Kajumbula H, Bazira J, Kansiime C, Asiimwe BB. Antimicrobial resistance at the human–animal interface in the Pastoralist Communities of Kasese District, South Western Uganda. Sci Rep. 2020 Sep 7;10(1):14737. doi: 10.1038/s41598-020-70517-w 32895433 PMC7477235

[37] Mochabo KM, Onono JO, Oluga GA, Nanyingi M, Mbaria J, Fevre E, et al. Mapping of Livestock Value Chains as a Tool for Understanding Disease Risks in Agro-Pastoral Systems of Kajiado County, Kenya [Internet]. In Review; 2022 May [cited 2023 Mar 27].

[38] de Garine-Wichatitsky M, Miguel E, Mukamuri B, Garine-Wichatitsky E, Wencelius J, Pfukenyi DM, et al. Coexisting with wildlife in transfrontier conservation areas in Zimbabwe: Cattle owners’ awareness of disease risks and perceptions of the role played by wildlife. Comp Immunol Microbiol Infect Dis. 2013 May;36(3):321–32. doi: 10.1016/j.cimid.2012.10.007 23219685

[39] Kansiime C, Mugisha A, Makumbi F, Mugisha S, Rwego IB, Sempa J, et al. Knowledge and perceptions of brucellosis in the pastoral communities adjacent to Lake Mburo National Park, Uganda. BMC Public Health. 2014 Dec;14(1):242. doi: 10.1186/1471-2458-14-242 24612845 PMC3975325

[40] Paige SB, Malavé C, Mbabazi E, Mayer J, Goldberg TL. Uncovering zoonoses awareness in an emerging disease ‘hotspot.’ Soc Sci Med. 2015 Mar;129:78–86. 25128439 10.1016/j.socscimed.2014.07.058 PMC4482355

[41] Byaruhanga C, Oosthuizen MC, Collins NE, Knobel D. Using participatory epidemiology to investigate management options and relative importance of tick-borne diseases amongst transhumant zebu cattle in Karamoja Region, Uganda. Prev Vet Med. 2015 Dec;122(3):287–97. doi: 10.1016/j.prevetmed.2015.10.011 26527312

[42] Onono J, Mutua P, Kitala P, Gathura P. Knowledge of pastoralists on livestock diseases and exposure assessment to brucellosis within rural and peri-urban areas in Kajiado, Kenya. F1000Research. 2019 Nov 13;8:1916. doi: 10.12688/f1000research.20573.1 33204408 PMC7642991

[43] Jensen KK, Lassen J, Robinson P, Sandøe P. Lay and expert perceptions of zoonotic risks: understanding conflicting perspectives in the light of moral theory. Int J Food Microbiol. 2005 Apr;99(3):245–55. doi: 10.1016/j.ijfoodmicro.2004.09.004 15808359

[44] Waldman L, Hrynick TA, Benschop J, Cleaveland S, Crump JA, Davis MA, et al. Meat Safety in Northern Tanzania: Inspectors’ and Slaughter Workers’ Risk Perceptions and Management. Front Vet Sci. 2020 Jun 18;7:309. doi: 10.3389/fvets.2020.00309 32626728 PMC7314929

