# Supplementary material for: Prioritization of livestock diseases by pastoralists in Oloitoktok Sub County, Kajiado County, Kenya
Source: PLoS One. 2023 Jul 12;18(7):e0287456. doi: 10.1371/journal.pone.0287456 (PMC10337939; doi:10.1371/journal.pone.0287456)
Supplement: S1 Data — (ZIP) [file pone.0287456.s001.zip › Oloitoktok transciptions/IDI F 9.docx]

**IDI**

How long have you kept livestock?

I was born and raised in a livestock keeping household. I have kept and been near livestock all my life.

Which ones?

Sheep goats and cattle.

Why do you keep these animals?

I sell the animals to buy food and clothes and feed the children.

Grazing areas?

Alongside Amboseli but not inside the park. During the drought we take the animals to Risa and Olgulului which is on the side of Risa.

Do you ever take your animals to Tanzania for pasture?

We don’t go to Tanzania.

What are some of the challenges that you face?

Diseases are one of the major challenges. Sometimes they have ticks. Drought also is a challenge and we move livestock to various parts and then we don’t have enough pasture for the cattle because the animals exhaust all the available pasture and then we have to purchase fodder for the animals.

Common livestock diseases?

Olorobi and eriri and nunuk.

Others?

For goats and sheep kileny and olmillo and olekipei. Those are the ones I know.

Please tell me more about Olorobi?

It is found in cattle and shoats.

What are some of the signs?

The feet are sick, salivating and doesn’t graze and also doesn’t drink water.

What about Nunuk?

The animal is weak and unable to move. There is also no milk let down. Nunuk is for cattle.

Kileny?

This one affects the hind limbs and they become weak and fall when they try to walk.

Any other sign?

None.

What animals suffer from kileny?

It affects sheep and goats.

What about Olmilo?

Only sheep and goats.

Signs?

Makes weird sounds (screams to demonstrate) that is the main sign.

Olekipei?

This disease kills animals. The sign is heavy breathing and then it dies

Any other signs?

None.

Which of these are zoonotic?

Sometimes you find we have running nose, sneezing and coughing and that is olorobi. For olorobi it is transmitted to people through milk.

Treatment for olorobi?

For animals I don’t know the drug they use I just see men injecting. For people we go to the hospital. We also use “oremit” which makes us vomit. We take the roots and the bark then boil them and put sheep fat. After consuming these we vomit and the illness is cured.

Even for kids?

Yes, from toddlers and above.

When do you go to the hospital then?

If the oremit doesn’t work then we go to the hospital.

Any other that can be transmitted to people?

Only olorobi.

How do you identify a sick animal?

“Isuuro” and hair coat changes. And for FMD the animal limps.

Are there any traditional methods that you use to treat animal diseases?

None.

When do you call an animal doctor?

We call them when olorobi is severe and when all the animals are sick.

Any zoonotic diseases that you know?

None.

Are there diseases that are transmitted from wild animals to livestock?

When the graze in areas with wild animals they also get sick. Mainly the wildebeests, when the cattle feed there they get a disease that causes blindness in livestock.

Any other diseases?

I don’t know any other.

Precautions to prevent diseases from wild animals to livestock?

There is nothing we do.

How do you prevent olorobi from livestock to animals?

We boil the milk.

Are there some who take raw milk?

We used to but now we don’t.

Why did you stop taking raw milk?

Because of olorobi.

Are there any who still take raw milk?

Sometimes the young men take raw milk when they go to Risa for pasture.

Diseases from raw milk other than olorobi?

I don’t know any other.

Raw blood consumption?

Nobody takes raw blood.

Assisted parturition with bare hands?

You just help the animal and wash your hands when you are done.

Any risk for disease from this practice?

No risk for disease.

Residing with livestock?

Yes, we do. We do for the young kids for shoats so that they don’t suckle at night and thus we can milk in the morning.

Any risk for disease?

None

Ever heard of brucellosis?

I have heard people who were sick and they said it was brucellosis but I do not know the signs of the disease.

Know anyone who has had the disease?

I have only heard; I don’t know any.

Have you ever heard of a disease called Anthrax?

I once heard about it a long time ago.

Please tell me more about it?

I have only heard about it and not experienced it here in my home or area so I don’t know.

Does brucellosis affect animals?

No, it doesn’t.

Rabies?

I know about it and we kill the rabid dog.

How does it transmit rabies to people?

When a rabid dog bites someone it can transmit rabies. I know it can transmit but I have never seen someone infected.

Would you like more information?

I would like to know but right now I do not see well so even if you train me, I cannot see anything. For the rest of the people, you can call for the meeting and train them so that all the people have the knowledge.

Any question?

I don’t have any question. I just know about cattle the young people are the ones that need to be taught but for me I am very old.

END
