# Supplementary material for: Prioritization of livestock diseases by pastoralists in Oloitoktok Sub County, Kajiado County, Kenya
Source: PLoS One. 2023 Jul 12;18(7):e0287456. doi: 10.1371/journal.pone.0287456 (PMC10337939; doi:10.1371/journal.pone.0287456)
Supplement: S1 Data — (ZIP) [file pone.0287456.s001.zip › Oloitoktok transciptions/IDI F 5.docx]

**IDI**

I: How long have you had livestock?

P: I was born in a family with livestock.

Which animals do you keep?

Sheep and goats and cattle.

Why do you keep these animals?

For milk, meat and for food. And when I have a problem, I sell some livestock to get money.

Where do you graze your animals?

They go far in all directions and sometimes they can go very far.

What about during the drought season?

When it rains, they go around the area but during the drought season they go very far to Chyulu hills and Kisongo in Tanzania and Mt Kilimanjaro and even Namanga.

What challenges do you face as pastoralists?

One is drought and then when the animals eat the short grass mixed with sand and then they drink water they die. There are also diseases like olorobi. Olorobi signs are lameness and salivating and the animal cannot walk or graze and it has wounds in the mouth. We use tetracycline to treat the animal and sometimes ash on the back. Another disease is olodua which gets to the kidney and the kidney enlarges and then bursts. Also, Oltigana; The animal is not able to graze and the kidney enlarges also and is not able to graze and not able to urinate and the animal eventually dies. It is like olorobi.

Any other?

Eng’oroto in which the animal gets weak, the hair coat changes and the tail falls off.

MCF,

Yes, sometimes we see MCF here which happens when the wildebeests give birth and urinate on the grass then the livestock feed on that grass and they get MCF. They then become blind and eventually die and the head also gets swollen.

Any other disease?

Enariri you find the animal gets pox on the skin and there is no drug to treat animal is weak and there is no cure and the animal cannot walk and eventually it dies. Only God can cure that one the whole skin becomes sick. No treatment works even teramycin and penicillin it can get healed or it dies.

Any other?

Olekipei the animal coughs and when slaughtered the lungs are damaged and that is what kills the animal. Olmillo also the animal circles and the animal does not die immediately it can last three years and eventually it is killed by hunger because it is unable to feed and when they slaughter it has water in the head. Eriri is also for shoats and olodua too for shoats and cattle. Those are the major diseases.Sometimes during drought they lack food because the animals have migrated and they lack food so they sell the animals to buy food.

Which of those diseases can be transmitted to people?

Olorobi and enariri and the latter they don’t eat meat or drink the meat thye think the disease is transmitted to people. God has prevented olodua from getting to people.

Eriri always goes to people since kitambo or lately? New knowledge maybe?

Eriri when ypu eat meat from the animal you also get pox and die so they don’t eat the meat

Signs in people?

Eriri used to be there many years ago but not anymore but there is also chicken pox but this one is manageable with herbs and it affects children but manageable

How do they treat chicken pox?

A long time ago if you find that the child has pox and it is com out you take olngosua herb and fat from animal feed the child and the pox dries but if the child the pox is not out you put olngosua and steam the child with it. And then the child swates very much and then once the water has cooled you wash the child with that water and they still do that even today.

Hospital for chicken pox?

No the child gets healed

Olorobi signs in people?

Coughing, body weakness but with no cough and headache and joint pains

Treatment olorobi?

Coughing they take oremit which tey boil mix with sheep fat and milk and it is very bitter and add salt and makes one to diarrhoea and vomit and they are healed after that.

Hospital?

Yes

When hospital?

Whne you take the herbs and you are not well the homa is extending then now you go to the hospital

Are these the only zoonotic?

Yes only these two. There was eriri for people along time ago and God removed it because people were not able to treat it. Chicken pox is different from eriri which we were unable to treat. Eriri was there when they were very young and there were some people who got it and were healed and they still suffere from other diseases like TB.

Signs of eriri then?

It was like olorobi coughing and shivering and then things come out of the skin like spots black spots like sand on the body and coughing. Not pox

Sick animal signs?

“Isuuro” (cannot eat, cannot graze and separates itself and the ears drop not feeding). When an animal does that we inject penicillin, small red tablets tryps medicine (osoirodo) and tetracycline (teramycin).

Herbs to treat animals?

When there is retained placenta they take oiti the bark of the tree boil it mix with magadi and give animal to drink and then after it comes out you inject tetracycline.

Why tetracycline and it has come out?

You remove the afterbirth but there is an infections that made the after birth be retained so the tetracycline helps with thaht. Fro nunuk thye use ash because the animal is weak and legs not moving so they pour ash on the whole body and force the animal to stay in the sun for the ash to penetrate.

Use of livestock officers and when?

Sometimes they call when the disease becomes extreme like olorobi to prevent and treat.

Raw milk consumption?

No we boil

Why boil?

Someimes an animal may look healthy but it has a disease that has not come outso they want to kill the germs of that disease

Any who take raw milk?

Laughs…the boys and those are mad people because those ones take raw milk.

Any disease from raw milk?

When an animal has olorobi or eriri we don’t milk so tha t it does not transfer the disease to us

Raw blood?

Laughs..we don’t take we never do. But the boys do take it. Even in the meat we don’t take raw blood

Why no raw blood?

You find that the diseases are in the blood so you might get the disease so we are avoiding the diseases.

Hnadling anial birth prodcuts?

No we don’t use gloves. We just handle the animal with bare hands we never wear gloves.

Risk for disease?

No disease

Residing with livestock?

There is no problem because we always put bu the person who doent like the animals will not like the smell but there is no disease

Wild animals diseases to livestock?

There is MCF because of the afterbirth from wildebeests have bacteria. Alos en’gororo (tryps) comes from chulu that’s when the animals get it after chyulu. Tryps is caused by some insects which are found in chyulu.

Ever heard of brucellosis?

I have ever gotten it and was injected with 25 injections. They call it ugonjwa wa maziwa and when I got it we did not have milk and the clinicians are telling us it is from milk, how now? When I got it it was drought so I don’t think it is from milk

Signs?

Joint pains, backache, unable to walk, pain all over the body

Is it from raw milk you think?

When we used to take milk during the rainy season we didn’t get it and got it in drauight season when were not taking the milk

Why milk disease?

These doctors lacked another name to call the disease so they called it milk disease but it is not from milk.

Anthrax?

Not sure about it.

Brucellosis treatment for herself? Herbal medicine?

Yes, We take a lot of drugs before like 5 types of herbs and we try all of these and after failing to work you go to hospital then when that is done we take herbs agin. The herbs after injections are to make you better because you don’t feel ok.

Education on zoonotic diseases?

I would like to know what causes them and the treatment

Best way to pass the information?

I would like you to call for a big meeting where men and women sit togetjer

Why big meeting and men and women together?

We don’t want to hear from others we all wan t to hear the information at the same time. If some people don’t get the information they will think that they have been neglected and despised.

Any question?

None becase you are the experts so we need to hear from you

I explain about community education after analysis.

END
