# Supplementary material for: Prioritization of livestock diseases by pastoralists in Oloitoktok Sub County, Kajiado County, Kenya
Source: PLoS One. 2023 Jul 12;18(7):e0287456. doi: 10.1371/journal.pone.0287456 (PMC10337939; doi:10.1371/journal.pone.0287456)
Supplement: S1 Data — (ZIP) [file pone.0287456.s001.zip › Oloitoktok transciptions/KII 1.docx]

**KII I**

I: What is your role in this facility?

P: I am a nurse in charge of this facility and involved in outpatient work, FP and MCH program and we also offer the ARVs as this is a CCC center. I have been here for seven years.

I: What is your role in terms of disease surveillance?

P: We try to do diagnosis by taking the history and also physical diagnosis. We also have a lab and we refer patients there. We treat them here and if we cannot treat, we refer them to the Oloitioktok subcounty.

I: What are the common human diseases?

P: Upper respiratory tract infections, diarrheal diseases, pneumonia, sexually transmitted infections, pneumonia, diarrhea diseases, anthrax sometimes and brucellosis also sometimes and we also have…the most common are the upper resp tract infections.

I: So, the zoonotic diseases are anthrax and brucellosis?

P: Yes, those two.

I: What about rabies?

P: Since I came here, I have never experienced rabies but we have cases of dog bites.

I: Is brucellosis common here?

P: It is periodic, you know when they migrate, we don’t have it. But when they are here, we have a lot of cows here and that is the time we have brucellosis because we have a lot of meat and milk here. That is why it is common. Animals migrate to chyulu hills but when they migrate back like now because of the rains we have a lot of brucellosis and anthrax because the milk is a lot now.

I: When do they migrate?

Like now they are migrating from the end of last month up to the end of this month they will be migrating to chyulu they go and come for water. They go and camp there only coming here for water so it is back and forth. They stay in chyulu until there is rain even 4-6 months. It is the young men who go with cows and when they come cases of B go high. We say anthrax is high here than brucellosis because they are always eating milk and meat and they know the signs in humans because many times with anthrax one gets a wound. They say this is anthrax.

I: Brucellosis do they know?

P: They just present with signs until you diagnose it through the lab. Brucellosis and Anthrax are the main zoonotic diseases here.

I: Do you offer regular education to community?

P: Oh yes every morning we educate them here before we start and we also we do that in barazas. We have a CHA (community health assistant) and CHV who go around in the village and we have divided the village into six areas and we have two CHVs in every area. Every time they have a meeting with the community, they call us to educate the community and the CHA also goes.

I: Please tell me more about CHVs?

P: They are volunteers and we train them so they can do first aid and also treat minor illnesses like headache and ORS for diarrhea. For the CHA they are employed by the government and she has done something to do with the community. She helps with ANC and tracing of defaulters too assisted by the CHVs.

I: How do you train the community?

P: We do the mobilization and call them here or go to their barazas. They always have a slot for us even political rallies and sometimes we organize them ourselves.

I: Do you educate them on zoonotic diseases?

P: Yes, we do. Especially the anthrax because that is what they really fear. Brucellosis they don’t really fear so we just touch on it a bit. They fear anthrax because they say that it recurs. Even an old mama can say they had anthrax along time ago and it is still causing them trouble. They just fear it. For brucellosis they don’t fear it like anthrax. So, we mainly teach on anthrax.

I: For brucellosis what do you tell them?

P: We ask them not to take raw milk or uncooked meat and then in case of any signs they should report to the facility and we discourage them from taking herbs.

I: Has this education led to behavior change?

P: Ah, I may say when they come here, they change but when they go to the bush they don’t, the morans milk and are in a hurry to go look after livestock. They don’t even have time to light the fire. And it is also changing because we are educating them not to take raw milk and not to eat uncooked meat.

I: So, brucellosis is there but when they move back with livestock. How many cases of brucellosis have you seen in the last one year?

P: We have someone on treatment for brucellosis right now and we see like one in a month but for anthrax we see a lot more often maybe weekly. We also have a program on zoonotic diseases that I mentioned about that is helping us educate the public. The brucellosis patient was diagnosed in Oloitoktok.

I: Health seeking behavior of the pastoralists here?

P: I may say that at least 80-85% are coming to the facility and the rest are using herbs. Here there are a lot of herbs and people go around selling them.

I: What about those who go to Chyulu in terms of seeking health care?

P: They use herbs but they also come to the facility and say there has been no improvement. We also take proper history because sometimes the herbs and the conventional medicine may not correspond like anti-malarial and herbs. Sometimes we give IV fluids to deal with the herbs. Men are the ones that use herbs women and children not much. Especially children they bring to the facility.

I: How is brucellosis diagnosed?

P: We can diagnose it here but the patient that I mentioned had been referred to Oloitoktok for another condition and then they tested and found that he had brucellosis. He was not tested here because he had an eye condition and then we referred him and then he was tested for B.

I: Do they buy medication from chemists?

P: Sometimes they buy because sometimes we don’t have all the drugs that we need. They buy like Panadol and brufen but antibiotics they know they should seek a prescription. Women especially do not because we have educated them.

I: Do they come to hospital immediately for anthrax and brucellosis?

P: For anthrax they wait for the boil to be ripe. They wait for some time because they say that if you take the boil to the hospital, they interfere with it and then it is worse so they delay to go to the hospital. For brucellosis they do not delay they could only take the herbs.

I: What about collaboration with vets for zoonotic diseases?

P: That is a challenge because our vets are few, we don’t see them. They only come if there is an outbreak or they have an activity like a vaccination exercise. It is a challenge; I think they are few so they only come when there is an outbreak.

I: Do you have joint meetings with vets?

P: No, we don’t have. We only see the private practitioners but the government ones I have never seen.

I: Is the collaboration necessary?

P: Yes very because they can also educate people and we can also report to them so we tackle these diseases together. We should have a county team for vets and a county team for us plus chief, nyumba kumi in collaboration so all of us together. We would support one another. There is a lot of laxity and shortage of workers especially on the vet side but us we are always here.

I: At what level should the collaboration be?

P: We should do It even here at the local level. This is where the problems are and there is a lot of knowledge gap that is why we need them here.

I: Please tell me more about the forum?

P: Yes we should have one here….a clinician like nurse, clinical officer and a lab person also nyumba kumi and chief and any other partner. Like one of our key partners is Maasai Wilderness Conservation Trust. I am the only county employee the others in the lab and clinical officer and medical officer are all from the organization. The CHA and the nutritionist are from the county govt. The partners have the manpower like transport and they can educate and they do a lot with the community and they have networks in the community so they would be very helpful.

I: Do you think that Zoonotic diseases are prioritized?

No, they are not. Like Brucellosis is not prioritized and we see these diseases on the ground.

I: Why are they not prioritized?

P: I have never heard anyone talking about these diseases but for HIV, STIs and upper respiratory tract infections, ANC, immunization these we talk about all the time. Even all my messages from the bosses in Oloitoktok are not about zoonotic diseases unless the vets wanted to know. For example, weekly we report on malaria cases and that must be done to the county and then to the National level. So, if this one of yours (zoonotic diseases) was the same way then it could be better. For brucellosis we report but I don’t think they even capture in their data. For HIV one has to report each new case, viral load, CD4 count and whether they have started ARVs. But for brucellosis none of that information is needed. It is important and we should have a template for reporting on brucellosis. And the vet should also be informed.

I: Why do you feel the vet should be informed?

P: Because that is the only way that we can educate people and the leaders too to know how we are treating it. When it is not reported then it is forgotten and people will keep saying it is not there. It is the same way that we say that we don’t have yellow fever in Kenya because we are not talking about it. So, it should be reported all the way to the national level because an alarm will be there that it is a problem. People are being affected and they are unable to work and it affects them psychologically if they are not treated comprehensively and on time. So, it is a big problem. However, we have a zoonotic program and we report each day on zoonotic diseases to the program. They are from a university in Kenya and we have 3 tablets and we record each patient we see and the signs then we send them to the lab and the report is also uploaded and then they see if the results were positive or negative. We collect blood and they test and let us know. The program has been here for almost a year now. The program managers used to come almost each month. They are doing a research and they are checking all the cases of zoonotic diseases. They need us to investigate all patients with a temp of 39degrees they test all of them. We used to do it routinely but they are empowering us to be more focused. For us we report anthrax and brucellosis. They chose this facility and some place in Kajiado Central. The program has benefitted us because they give us specimen and lab reagents and machines and the tablets that make out work easier because we do our things electronically even from the clinician to the lab we do all this electronically.

END
