# Supplementary material for: Prioritization of livestock diseases by pastoralists in Oloitoktok Sub County, Kajiado County, Kenya
Source: PLoS One. 2023 Jul 12;18(7):e0287456. doi: 10.1371/journal.pone.0287456 (PMC10337939; doi:10.1371/journal.pone.0287456)
Supplement: S1 Data — (ZIP) [file pone.0287456.s001.zip › Oloitoktok transciptions/IDI F 4.docx]

**IDI**

I: How long have you kept livestock?

P: I grew up in a livestock keeping home.

Why do you keep livestock and what kinds do you own?

I keep cattle (ngishu), sheep and goats (ndare). I sell them and get money for school fees, milk and meat. We also slaughter and eat the meat.

Where do you take your animals for grazing?

We graze them at chyulu hills because it is greener there. It is not that far although they don’t go up to the hills when there is grass here. They go when it is dry here usually in Aug to Oct. They come in Nov when it starts raining.

Are there wild animals here?

In this area there are a lot of wild animals so they graze together with livestock eg zebras, lions, rhinos, elephants, hippos.

Do you ever take livestock to Tanzania for pasture?

No, we don’t go.

What challenges do you face as pastoralists?

Number one is lack of water. During drought there is no water especially in Chyulu so when the animals are there we have to buy and transport water to the animals which is expensive. The lack of adequate pasture during drought is also a big challenge here and when we take the animals to the game reserves we are kicked out by the rangers.

Please tell me more about the wild animals/livestock interaction here and in the game reserves?

We go to Tsavo national park although sometimes we are asked to get out of the park and it is the only place with grass. Another challenge is the hyenas at the hills and lions which eat the animals. We also have diseases like ECF so when they go to the hills there are ticks which transmit ECF (oltigana) and many animals die.

What are the common livestock diseases here?

ECF, Eng’oroto (trypanosomiasis).

Kindly tell me more about ECF?

It is common when the animals go to chyulu hills although it is there all the time and caused by ticks. The signs are the animals struggle to breathe, the cow smells such that when the animal dies you cannot eat the meat and also emaciation.

When is this disease most experienced seasonally?

ECF is common when it rains. When it rains there is also FMD (olorobi) which affects the legs and the mouth of livestock. Also there is olekipei which is CCPP which kills animals a lot more than other diseases. This one is common all the time.

Any other diseases?

For sheep there is olmillo which makes the animals circle. We think it is caused by the dips we use and affects the CNS and the animal goes round and round.

Other diseases?

There is CCPP (orekipei) and also lumpy skin disease as well as sheep and goats disease which we call enariri.

How do you identify a sick animal and what do you do?

“isuuro” that is how we know meaning the animal is dull, doesn’t eat, goes alone and has rough hair coat. We give the animal an antibiotic called tetracycline which we always have around the home.

Do you ever call livestock officers to treat animals?

If the disease is extensive and affecting a lot of animals, we call a doctor but not when an individual animal is sick.

Traditional cures for sick animals?

There is another disease called nunuk (three-day disease). So, when we realize the animal has the three-day disease, we take ash and apply it on the skin and boil the bark from the “oloiti” tree and give the water to the animal to drink. We also use another herb called “osuguroi”.

Why not teramycin for this nunuk disease?

We just use the tetracyclin and the herbal medicines.

Why do you use teramycin for these diseases?

Because it is an animal drug that is very effective.

Do you know any zoonotic diseases?

We have brucellosis “engeya gule” and olorobi too. When animals have FMD we don’t take raw milk because when we take the raw milk, we also get olorobi which is the common cold.

Please tell me more about brucellosis?

I don’t know how it is transmitted but I hear it is from cows’ milk but I am not sure. It is diagnosed when someone goes to the hospital.

Any other Zoonotic diseases you know about?

None other.

Do you boil milk before consumption?

Yes, I boil the milk we don’t take un boiled milk in this homestead.

Do you know any zoonotic diseases which can they be transmitted from wildlife to livestock?

“nguruya olchang’et” MCF which is caused by the buffaloes when they give birth and livestock feed on that contaminated grass. The grass is contaminated from the birth fluids of the buffaloes. But it is not a common disease here.

So, for zoonotic diseases that she knows which one is most severe?

The most severe one is brucellosis because you have to go to the hospital and get a lot of injections but for FMD it is a common cold which one can recover at home. So brucellosis is more severe.

Do you know anything about brucellosis in livestock?

I do not know about it in animals.

Did you say that boiling milk is protective for FMD and brucellosis?

Anything that is heated kills the bacteria so yes.

Any other protective measures against these zoonotic diseases?

We boil the milk and also if the animal is that sick, we slaughter and we don’t throw away the meat so we cook and re cook the meat for a long time so that the bacteria can die. That is done if the animal was sick.

Any zoonotic diseases in your herd?

FMD is very common in cows here.

Health seeking behavior for Brucellosis in particular?

We take the person to the hospital and don’t try to treat it at home. We have the common herbs but for brucellosis we take them to hospital to get the 21 day injections because weknow that is the right treatment.

Do you know any other transmission mechanisms for brucellosis?

I only know it is transmitted through milk.

Ever had brucellosis case in your household?

In the neighborhood but not in my household. I cannot talk about the person who had it I will keep this information confidential. I hear about brucellosis but I don’t know much about it.

Symptoms of brucellosis in humans?

Headache, joint pain, inability to move the legs.

Would you like more information on brucellosis?

Yes, I would like more information.

Best way to reach people here with this information?

You should call for a meeting like in a central venue such as a church and train all of us together.

END
