# Supplementary material for: Prioritization of livestock diseases by pastoralists in Oloitoktok Sub County, Kajiado County, Kenya
Source: PLoS One. 2023 Jul 12;18(7):e0287456. doi: 10.1371/journal.pone.0287456 (PMC10337939; doi:10.1371/journal.pone.0287456)
Supplement: S1 Data — (ZIP) [file pone.0287456.s001.zip › Oloitoktok transciptions/IDI F 8.docx]

**IDI**

I: When did you start keeping livestock?

P: I grew up with livestock. I keep cattle, sheep and goats.

Why do you keep these livestock?

When sick we slaughter and take the fat, also we sell and use the money to take someone to the hospital when sick, when there is no food we milk and drink the milk. We also use the cream from the milk.

Where do they graze?

We go to the forest and come in the evening.

Other areas?

We also go to a place called olteyani in Lengisin ward and stay there during drought. We go there in Jan.

Do you go for pasture in Tanzania?

No, we don’t.

What are some of the challenges you face as livestock keepers?

When there is drought, we take them to Olteyani and they die sometimes when the drought is severe. They also die from diseases caused by ticks which are from wild animals. Another thing is diseases like nunuk. For this disease we apply ash on the back. There is also eriri which is LSD which you administer medication for. In sheep there is olekipei. Then there is olorobi also and the other CNS disease when they go to pasture and it is called olmillo but in cows it is called engeya ologuny.

Kindly tell me about olorobi?

This is a disease of cattle and shoats but mostly cattle because it affects the legs.

What are the signs?

“Isuuro” and salivating and not able to walk.

Transmittable to people?

Yes, one gets headache and you take drugs. When you have FMD you take herbs or take brufen to relief the pain.

Other signs in people?

Only the headache.

What herbs are used?

Olgonyel also rob which you apply on the forehead.

How is it transmitted to humans?

When cows have olorobi you find people have it too mostly kids coz of the milk and then they have things on the mouth. The mouth turns white and so you know it is olorobi.

Nunuk?

Only for cows.

Signs?

Abnormal urination it doesn’t urinate because it is very sick, cow dung is hard and you prevent it from taking water.

Transmittable to people?

No.

Enariri?

It affects both people and animals.

Signs in cows?

Nodules and patches on the coat.

In people?

People get pox or patches on the skin. “masanduku”

Olekipei?

It affects sheep and goats.

Signs?

Coughing and then it dies.

Other signs?

None other.

To people?

No.

Engeya Ologuny..(heartwater disease or MCF)?

The animal becomes blind and it has a lot of challenges. Also, salivation. It is transmitted from wild animals like zebras and affects mainly cattle. And Olmillo is also a CNS disease affecting shoats.

To people?

Olmillo No and Engeya no

Signs olmillo?

The animal isolates itself and moves away on its own and diarrhea.

Any other livestock diseases?

Only those.

Any zoonotic diseases you are aware of?

Yes, Olorobi and eriri.

Which one is more severe?

Eriri is bad.

Why?

Because it kills the children although we have not encountered it in quite a while. But we know it is severe.

Any eriri in animals in the recent past?

Yes we have seen it in animals.

What about olorobi?

This one is always there.

Do people always get it?

Yes, many people get it here.

Treatment options for olorobi?

We use taramycin for animals.

For people?

We use herbs and we also go to the hospital. If the disease is severe we go to the hospital and this is when one has body weakness and is unable to walk.

Eriri treatment?

In children we give the child fat from meat and wait for all the pox to get on the skin before taking the child to the hospital. When a child has the pox, the child has fever and unable to breath and the pox is not out all over the body we don’t take the child to the hospital yet until the pox is all over the body on the skin. If the child is injected before the pox is on the skin the child gets severely ill. So, we have to wait for the pox to come out first. The fat from the sheep meat helps the pox to come out.

Any other herbs used?

None.

Do you take raw milk?

We cook the milk completely before drinking it.

Are there some who take raw milk?

None, because we are afraid of olorobi.

What about consumption of raw blood?

The young men take a lot. They use a bow and drain blood and drink it.

Why young men?

It is a culture we used to have and they have continued with it.

Why have others stopped doing it?

It is sweet and people like it but some have stopped.

Do you drink raw blood?

I don’t drink it but I eat blood mixed with meat but I don’t like raw blood.

Any diseases from raw milk?

Eriri and olorobi.

Are there any diseases that humans can get from taking raw blood?

None.

Are there any risks from assisting with parturition?

None at all!

What about residing with livestock?

There are none.

Which animals do you reside with?

Kids and calves so that they are not rained on.

Why is not good for them to be rained on?

I just love the caves and cannot let them be rained on.

Any diseases from this?

None.

Are there any diseases that can be transmitted from wild animals to livestock?

Only the heartwater disease.

How is it transmitted?

When they graze together, they transmit to each other.

Have you ever heard of brucellosis?

Yes, I have heard of the milk disease even another one called water disease.

Please tell me more?

Some people go to the hospital and they are told they have brucellosis even when there is drought and no milk. I have never encountered someone with the disease though. I just hear from people.

Can it affect livestock?

No.

Have you ever heard of anthrax (Emburuo)?

I associate anthrax with eriri and when the animal dies, we don’t eat the meat and we also don’t take the milk because we say it will transmit to us so we bury the carcass.

Ever heard of rabies?

We know of rabid dogs which bite all other dogs and it can also bite a human.

Do you purchase over the counter medication?

Yes, we do buy like Panadol and brufen.

How do you determine which ones to buy?

We just tell the doctor I need medicine for a headache.

How do you determine a sick animal?

The cow won’t have milk and the hair coat will be dull. We use teramycin because it cures the diseases.

If no recovery?

It dies we never call a doctor.

Why never call a doctor?

We just don’t

Would you like more information about diseases that can be transmitted from animals to humans?

Yes.

What would you like to know?

The diseases that you know.

Anything else you would like to know?

I would like more information on zoonotic diseases.

Best way to give this information?

A group discussion would be best. Because we can be together and all the people would have the information.

Thank you. Any questions?

When will you come for the training?

I clarified about data analysis and how we will come back to give them feedback.

Ok, thank you very much

END
