# Supplementary material for: Prioritization of livestock diseases by pastoralists in Oloitoktok Sub County, Kajiado County, Kenya
Source: PLoS One. 2023 Jul 12;18(7):e0287456. doi: 10.1371/journal.pone.0287456 (PMC10337939; doi:10.1371/journal.pone.0287456)
Supplement: S1 Data — (ZIP) [file pone.0287456.s001.zip › Oloitoktok transciptions/IDI F 3.docx]

**IDI**

I: How long have you kept livestock?

P: I was born in a household keeping livestock.

What kinds of livestock do you keep?

Cattle, Goats, Sheep and chicken. These are helpful because when I need money for school fees, I can sell some and pay school fees. Also, for milk and meat.

Grazing area?

Close to home but when there is drought in July/Aug to Dec they go to chyulu hills.

Who takes them?

Young men and men most of the time.

Do livestock here interact with wild animals?

Yes, a lot even yesterday one of my goats was eaten by a hyena. There are also lions.

Other wild animals?

Elephants, zebras and wolves too which prey on goat and sheep kids.

Do you ever take livestock to Tanzania?

No we dont.

Challenges as livestock keepers?

The wild animals are too many here and they eat our livestock a lot. Also, diseases and drought.

Common livestock diseases?

“Eng’ororo (trypanosomiasis)….eriri (LSD), FMD, MCF

Seasons for these diseases?

Trypanosomiasis is common during drought, Eriri and MCF all the time and FMD when it is raining.

Any diseases common when animals are at chyulu?

Yes, FMD and trypanosomiaisis.

How do you determine that an animal is sick?

For FMD it is excessive salivation and also “isuuro” when an animal doesn’t feed and is dull.

What do you do when you see these signs?

Tetracycline…”teramycin” which is the 10% antibiotic (oxytetracycline).

Do you use that for any suspected disease?

Yes, we do.

Are there any traditional remedies used?

No, there aren’t any.

Do you engage animal doctors for diseases?

Yes, when the animal is very sick we call them.

Do you know any zoonotic diseases?

Yes, olorobi (FMD).

How is it transmitted to people?

When you drink raw milk. Some give raw milk to their children.

Signs of Olorobi in people?

Coughing which is homa. And also, headache and diarrhea. It is common during the wet season around dec and Jan and that is when people have homa.

What remedies do they use to treat olorobi?

We go to the hospital but some buy mara moja or sona moja. For children we buy them paracetamol.

At what point do you visit a do health facility?

When one is coughing and has a headache.

Are there any traditional remedies used?

No, there aren’t.

Any other zoonotic disease? Brucellosis?

Never heard. Actually, I have heard about brucellosis but not much.

What have you heard?

I don’t really know it.

Are there any diseases transmitted from Wild animals to livestock?

I don’t know any.

Tell me more about MCF

I don’t know much about this disease.

Do you use gloves when assisting animals with calving?

No.

Is there any chance of disease transmission from this practice?

I don’t think so.

Residing with livestock?

Yes, we do reside with livestock although it is not good to do so.

Why is it not good?

I don’t know really; I just think it is not ok.

Do people here consume raw blood?

We stopped taking that a long time because Christianity discourages it.

What about the men?

The men take but not here at home. There in the bush when they go to take animals to pasture they don’t boil milk they take it raw.

Why don’t they boil?

They have no time for it.

Are there any other risks from consuming animal products?

Meat can cause problems like diarrhea if the source animal was sick.

Is it because of uncooked meat?

Yes, because some don’t cook the meat well and the person who is sick goes to the hospital.

Do you have any questions?

Why are you people gathering this information and why record the interview?

I explain why we record and why we are doing this research.

Do you delete when you are done with writing the data?

I explain about confidentiality and how we discard the recordings when done.

What are you going to do after this research?

I explain about the research and the feedback mechanism to policy makers and the community.

END
