# Supplementary material for: Prioritization of livestock diseases by pastoralists in Oloitoktok Sub County, Kajiado County, Kenya
Source: PLoS One. 2023 Jul 12;18(7):e0287456. doi: 10.1371/journal.pone.0287456 (PMC10337939; doi:10.1371/journal.pone.0287456)
Supplement: S1 Data — (ZIP) [file pone.0287456.s001.zip › Oloitoktok transciptions/KII 6.docx]

**KII**

I: What you do here as an elder?

P: I am the Chairman of chief advisory committee and elder of “nyumba kumi”. I am chairman of the chief committee and we are five of us. Also, in nyumba kumi I am the chairman heading the location in charge of many villages.

What are some of the challenges you face as pastoralists?

Many diseases, they have increased e.g. some cattle have died from anthrax and FMD also is a big challenge. It has reduced though, also goats “ugonjwa ya kichwa” which has no cure. There is also CCPP…(Olekipei) but that has a cure unless there is a delay in treatment. Now we have water close by especially here in Kimana. Diseases are the biggest challenge. We herd close to the homes because land has been sub-divided. In Sopa there is more herding space.

Where do you graze animals?

Close to homes unless when there is draught, we take to mbirikani group ranch where there is a lot of space. Only during drought but mainly here close to the homes.

About how far from here do people take their animals to graze?

About three km farthest. Most people do not keep a lot of animals here, most of our livestock are in other areas…we keep only the milking ones here. Mine are in Mbirikani where there is adequate space.

Do livestock interact with wild animals here?

Not much here unless in Aug and Sep when we request to take our animals into the sanctuary in Kimana. Sometimes they let us take the livestock. Challenge is ticks there and diseases like East Coast Fever but we still take them. We do this when there is severe draught which can be even after five years. They only allow when there is draught. Ticks are many and they make cows sick. There is no other interaction with wildlife because there is a fence although sometimes, they graze with zebras and antelopes. But these are not as harmful as they do not have ticks.

Do you ever take animals to pasture in Tanzania?

Few rarely. Those who usually do are those in Rombo and entonet but not here in Kimana. In Rombo they mix a lot with animals from Tanzania and they live well together. Here we don’t move a lot because there are farms so sometimes during drought we zero graze the animals and keep even grade cows. There they keep a lot of livestock sometimes even 3000. People are keeping livestock and we love animals and they are our bank. Cows have better returns than banks.

What are the common diseases of livestock?

Mainly FMD each year when it rains sometimes even twice a year. It is not harmful but because we are milking the cows you find that sometimes even humans get “homa” too from the milk. Also, anthrax, Ndigana before we had a dip ndigana was harsh but with dips the ndigana is not a big issue.

You said FMD is common when it rains?

Yes, when it rains.

Anthrax?

It is not a common disease and we don’t know what causes it unless you scientists tell us.

Ndigana?

It is because of ticks.

What diseases are common in sheep and goats?

Sometimes it is Olekipei and Olmillo and also, diarrhea. These are the main ones.

How do you treat sick livestock?

We treat them ourselves. I check the coat and the lymph nodes and treat them myself and they recover. We don’t call doctors; we treat them ourselves. We check the coat, signs like FMD (Olorobi ) we identify from the wounds in the feet and mouth. For anthrax we check the cow dung. The dry one is from anthrax or with mucous so we deny it water and then it recovers. We know what treatment to give for each disease. We use mainly taramycin and penicillin and the good thing with the latter is that it cures many diseases. Even for Olekipei which is very dangerous the penicillin helps although there is a specific treatment for it but sometimes it is unavailable because it needs to be refrigerated. So, you use penicilin first then it might recover. If it doesn’t then you find a doctor to treat it because they don’t often agree to sell us the Olekipei medicine. The ones with grade cows especially call doctors and these are mainly the Kikuyu. They are the ones that call the doctors or also a Maasai like I have treated a number of such cows myself. We have lived with livestock for a long time so we know what to do like the Kikuyu do with farming. Sometimes we seek a doctor if the animals do not recover.

Do you know about zoonotic diseases?

I don’t know (pause) but when cattle have Olorobi then even us humans we get ill. The “homa” in humans manifests as coughing and when you check it is when the cattle are sick even when the bushes are many people get malaria because it comes from mosquitoes. So, when there is FMD people have homa.

Any other zoonotic disease you know about?

None other.

What about Anthrax?

I don’t really know but we check the animal dung and also the animal has water in the fat areas. It is transmuted to people and it is causes a sore. We know that from the doctor. It is a boil and you are told not to burst it and then when it is ready you burst it. It comes from eating the meat from that animal. When you eat meat from a carcass that was infected with anthrax.

What about brucellosis in animals and people?

It is in people. And it is the same way you hear people being told they have “ugonjwa wa maji”. But we don’t know it in livestock. A lot of people these days are told they have “ugonjwa wa maziwa”. So, we ask how can it be from milk and we are boiling milk? These days we all boil milk even in the remote areas. We don’t take raw milk maybe morans in Nov who take livestock away and they milk and drink but it is not often.

Do you know the signs of brucellosis in people?

I don’t know. I have heard about it but I don’t know much about it. We hear people saying the doctor told then it is “milk disease” thye are suffering from but we don’t know. We call it “emoyian ne gule” meaning milk disease.

Rabies?

That we know and sometimes it happens in some occasions. We can get but there is some early treatment and if one is bitten, we rush them to the hospital for the injection so people rarely get sick but we don’t know what causes it. Sometimes they vaccinate the dogs and it has been a while maybe in the last three years and the county says there is no money to do that. We call it “olianalaligua”…”mbwa wa kichaa”. Once someone is bitten, we rush them to the hospital.

Any other zoonotic diseases?

None.

What is the treatment for brucellosis in humans?

When sick we go to the hospital and then they have 21 injections to cure the disease. Many go to the hospital.

What causes abortions in livestock?

Cows abort when they lack water and the sun is very hot. The sun causes abortions. And also, diseases like FMD so there are diseases that cause animals to abort like when water is far and it is very hot. Also, when a cow is infected with two diseases eg olorobi and anthrax at the same time a cow can abort.

And what causes mass abortions?

Yes, it happens sometimes that many cows in one locality abort at the same time and we do not know why that happens.

Have you asked the doctors?

No, we don’t ask.

How would you prioritize these zoonoses in terms of frequency and severity?

We would say it is anthrax and some people get it when we eat meat from butcheries also from milk so we don’t know how we got the boil.

Anthrax is most serious?

Yes. The other one is any disease that causes abortions.

Are there any diseases which can be transmitted from wild animals to livestock?

Yes, it happens when livestock goes to the sanctuaries. When ticks bite cows then they get sick. That is how diseases are transmitted. Also, there was a disease that is caused by livestock and wild animals grazing together. This happens when they share water sources which are contaminated with excreta from wild animals. It is “Ugonjwa wa kichwa” but I don’t know it well. Even goats are getting sick now from the same disease.

What are the signs of this disease?

They become blind and have an eye infection and there is no other issue. This happens when wild animals and livestock graze together especially in the remote areas where water is not readily available.

So please clarify again how you would prioritize these diseases you have already mentioned?

It is anthrax, FMD, the head disease, “the liver one”, Otigana which affects the tonsils and liver…this is otikana. And also “ile ya mapafu” CCPP. The one of liver is when animals graze in swamps also from tse tse flies. Ndigana causes liver issues.

What are some of the ways of preventing these zoonotic diseases?

Yes, by injecting the livestock. That is the only way. There is no way to prevent. Wild animals and livestock have to mix during drought. We take them to the sanctuaries in Aug for a short while only because that is how long they allow it for.

Do people know about zoonotic diseases?

They know though not well. We call doctors for CCPP and FMD when these are severe the rest, we treat them ourselves. Sometimes the doctors treat and they abort when they give the vaccines for these diseases. What can you do? They inject and sometimes they abort and we don’t follow up to know why the animals aborted. We think it is an unnecessary expense to follow up. We don’t even get bitter about it, we let it go. We love animals and keep them as our bank and cows are very profitable.

Do you have any questions?

Why are you doing this research? Can we get treatment for these diseases?

I explain the purpose of this study in detail. And feedback to them later on and how it will involve policy makers.

And brucellosis many people are getting it. Why?

I explain it in livestock, wild animals and humans.

Oh, I see. Yes we interact very closely with animals; sit on the floor on manure, the animals lick us so now I see how we can become infected.

END
