# Supplementary material for: Prioritization of livestock diseases by pastoralists in Oloitoktok Sub County, Kajiado County, Kenya
Source: PLoS One. 2023 Jul 12;18(7):e0287456. doi: 10.1371/journal.pone.0287456 (PMC10337939; doi:10.1371/journal.pone.0287456)
Supplement: S1 Data — (ZIP) [file pone.0287456.s001.zip › Oloitoktok transciptions/IDI F 10.docx]

**IDI**

I: How long have you kept livestock?

P: I have been in a livestock keeping home since I was born.

What livestock are you keeping currently?

Right now I keep cattle and also do farming. I have cattle and sheep because goats destroy the crops; maize and beans and trees. I also keep some chicken

What crops do you grow?

Maize and beans.

Why do you keep livestock?

Livestock are our bank; they are our source of income.

Tell me more about this?

When we need school fees for our children, we sell cattle. Even to farm we sell livestock to buy seeds for farming.

Where do you take the animals for grazing?

Mine are few so they feed in my farm. Even during drought, I zero graze the animals with maize stalks. Those with many animals take them to different areas in the bush and they keep looking for pasture in different areas.

Challenges as a livestock keeper?

I keep only dairy cattle and the challenge is to dip, inject and give drugs. Like now ticks are a big problem as well as FMD. Even people have FMD. Ticks cause a lot of wounds and we have to spray the animals every few days.

Have the ticks always been a challenge or is this a recent problem?

It is seasonal like when it rained recently, they increased even in the farms. The ticks cause FMD, right now that is the issue.

FMD signs?

Wounds in the mouth and the animal doesn’t feed and salivating also. That is how we know the animals have “olorobi” (homa) and we don’t have a good treatment for this disease so we just use penicillin and teramycine. We don’t have any treatment for the sores.

Any other signs of olorobi?

Only the wounds…we call it “homa”. Also, we call it olorobi.

Can this disease be transmitted to people?

Yes, even I are just recovering from olorobi. Every time cattle are sick even people are sick.

What were the signs of olorobi you experienced ?

Joint pains, tightness of the chest and panting so I knew it is homa and I also had no appetite too. We call it homa in people.

Treatment?

I went to the community health center and they gave me some medicine mainly brufen and other small white ones for the homa and I didn’t feel ok so I went to a private hospital where they gave me four injections and after that I felt well.

Did you use any herbs?

No, I didn’t use those.

Other livestock diseases?

Only FMD which makes it hard for the animals to walk. FMD is the main challenge. It comes like wind and comes before the short rains.

Zoonotic diseases?

Yes, I heard that FMD comes from wild animals and because they graze in the same area that is how they get it and then they transmit the disease to us. When they drink water from the same place that is how they get the disease. That is how we too also get homa.

Any other disease from livestock to humans?

None other only FMD. Also, eye diseases because there are flies and those flies come to our eyes and so we also get the same eye disease from the cattle and teary eyes. It is transmitted through flies. We call it allergy but it is eye disease from cattle. We call it eye disease. So, we say that it comes from flies. It is a big challenge

Treatment?

In hospital they give us eye drops but we don’t get very reliable treatment.

Do you boil milk?

Yes, we take boiled milk.

Why?

Because we have heard that there is a disease from milk. Even that “homa” I just told you about; boiling kills the germs. Olorobi is from taking milk and just being around livestock.

Are there those who take raw milk?

Yes, because we the Maasai …. some do because like us we know the importance of hygiene but some people here are not clean. Like us here they don’t consider us as Maasai because we don’t keep a lot of livestock and we do farm too which is not so common among the Maasai. So, these diseases are from lack of hygiene like water and soap for handwashing and washing the milk containers very thoroughly to ward off flies which bring diseases.

Where did you get this knowledge from?

It is God who has given it to me.

So nobody takes raw milk in this household, that is what you said?

None at all, we give babies colostrum but we ensure it is from cows that are not sick with olorobi. If a cow has olorobi even the calf will be ill and so you will know that the cow is sick.

Other signs of disease?

Weakness and lethargy and inability to walk so I know it is sick. Another sign is the rough hair coat and “issuro” so I quarantine the animal because the illness could be severe. I keep it alone and treat it tied on a tree and I also pour ash on the animal so that it gets a little warm and to protect it from cold.

Can an animal be sick and have no such signs?

When an animal is sick it is evident and it cannot move.

Do people consume raw blood?

We are saved so we don’t use it. We pour the blood.

Do you reside with livestock?

No, we don’t.

Is it a risk to humans for disease transmission when they reside with livestock?

Yes, the dung causes allergy and sneezing. When you reside with livestock the house becomes very warm and you sneeze a lot.

Any other disease?

Pneumonia and allergy.

Do you assist in parturition with bare hands?

Yes, bare hands.

Is there a risk for disease from this practice?

No, God takes care of us. We assist but with no gloves.

Do you call livestock doctors to treat animals?

No, we never call them unless they are in the area for some other work. I don’t know why we never call them. We treat and sometimes they recover and other times they die. We don’t eat the carcasses though we just discard the carcass. Some of the Maassi though eat the meat from the carcasses. We feed the carcass to the dogs but not to people. We treat livestock ourselves we don’t even look for the doctors we have never looked for them we don’t even know where they are we just purchase medicine from the shops and both my husband and I can administer the injections.

Do you use any herbs for livestock?

Teramycin, dawa nyeupe (penicillin) ,alamycin spray and warm ash on the coat of the animal for warmth when it is sick with FMD.

Do wild animals and livestock in the grazing areas?

No, they don’t for grazing but for water they are watered in the same water holes that wild animals use.

Have you ever heard of brucellosis?

Yes, I have heard just heard about it the same way I have hear of “water disease”. It has been around for a while and many people are getting it.

Why is it called milk disease?

We don’t know why it is called milk disease maybe it is from milk but we don’t know. We know water disease (typhoid) and this one is because we drink dirty stagnant water but brucellosis we don’t really know.

Signs of brucellosis?

Weakness and joint pains and headache. It makes it hard to even hold things.

Is it in animals too?

No not in animals and I don’t know why it is called milk disease. Is it from raw milk? And the clinician tells the patient not to drink milk, meat, beans and tea leaves. They ask the person to take drinking chocolate instead.

Have you ever heard of anthrax?

This one is there in animals. There is oozing of blood from the mouth, anus and nose.

Can it be transmitted to people?

It is common where animals are many and is not transmitted to people.

Rabies?

Yes, that one it is God who protects us. If we see a rabid dog; if it barks a funny way we know it is rabid. This disease is there.

Would you like to know about zoonotic diseases?

Yes, I would like to know about zoonotic diseases, I would like to know this olorobi how are we getting it and it is often very severe in us humans.

Best way to reach you?

A sit down so that many people can come and be trained and we will tell each other, also through the TV and radio. Not all have TV and Radio and not all people can understand the message if it is passed through the radio. But in a sit down it is workable because many people will receive the information.

Should we offer this education to men and women together?

Yes together.

Any question?

Point of the research?

I explain our study in detail.

END
