# Supplementary material for: Prioritization of livestock diseases by pastoralists in Oloitoktok Sub County, Kajiado County, Kenya
Source: PLoS One. 2023 Jul 12;18(7):e0287456. doi: 10.1371/journal.pone.0287456 (PMC10337939; doi:10.1371/journal.pone.0287456)
Supplement: S1 Data — (ZIP) [file pone.0287456.s001.zip › Oloitoktok transciptions/FGD W 3.docx]

**FGD**

I: Common livestock diseases?

P: Olekipei

Olmillo in shoats similar to engeya ologuny which is in cattle

Enariri

Engeya ologuny in cattle

Enarogua

Eng’oroto

Oltigana

Olorobi

Enado monyet

Enado gula

Nunuk

Olodua

Kububwo

Engeya enarogua

Olchanget

Erarasharash in cows

Kileny in shoats similar erarasharash in cattle

Olekipei?

It is in Shoats and cattle too.

Signs?

Coughing, heavy breathing, and then the animal will be emaciated until it dies. If we are able to treat, they get better.

Which medication do you use?

We use penicillin

Seasons it is common?

During the rainy season when the rainy seasons are ending

When is the rainy season?

It ends in April/May all the way to June.

Can it be transmitted to people?

No

Olmillo?

Shoats but mainly goats not cattle.

Signs?

The animals start running and circling and screaming and more so when you hold the ears and then it collapses. It also runs far away.

How do you treat it?

This one has no cure, we use teramycine but it doesn’t work. No medicine works for this disease. When it persists we just slaughter the animal. We try different strategies such as sugar water, cutting one ear but it still doesn’t work even tobacco. The bleeding from the ear we believe will make it recover and when we slaughter, we don’t eat the head because it has worms so we eat everything else and feed the head to the dogs. These are the strategies that we keep trying (laughter). If all these don’t work, we slaughter the animal.

Can it be transmitted to people?

No

Why don’t you eat the head though?

The disease is in the head so we avoid the head which has worms and also fluid. Even the joints have fluid and also God takes care of us we don’t get sick. The meat from that animal is very delicious though.

Seasons it is common?

All the time esp. when it is raining it gets into all the bomas. It has no cure so we have to slaughter the animal.

Engeya ologuny?

Only in cattle

Signs?

This one the animal becomes blind and keeps bumping into things so we give the animal teramycine and it doesn’t work so we sell it the animal eventually.

Seasons the disease is common?

This disease is MCF also called nguruya olchanget so when the wildebeests give birth and then they take water and urinate in the water then domestic animals take the same water so they get it. That is how it is transmitted to the cattle from the wildebeests. Chaget means wild animals.

Is it transmitted to people?

This is seasonal like now from May to June. When the wildebeests have given birth after that it is common.

Eriri?

Also called enariri is in shoats and cattle

Signs?

Rashes on the coat and the rashes persist then the animal becomes very sick so we don’t even eat the meat. It is a very bad disease and humans can get it. We throw the carcass to the dogs, we don’t eat meat from such a carcass.

Seasons it is seen?

It is not a common disease.

Is it transmitted to people?

Yes, we don’t milk a cow with eriri because it is transmitted to people.

Signs in people?

The same; rashes so we avoid milking it until it gets better and we don’t eat the meat

Eng’ororo?

This one is in cattle only

Signs?

The hair on the tail comes off so it has to be injected to get ok and then the animal becomes very emaciated. Also, the animal gets “mashilingi” on the coat. This one is found only in cattle

To people?

No but there is no milk let down from the animal

Seasons?

There are places when the animals go esp chyulu and they come with the disease. It is very common once the animals go to chyulu. We don’t know what happens there. Sometimes people say it is the leaves they eat there but we don’t really know.

How to treat?

There is a specific medicine for this disease a red one that we use to treat. Once treated they get better

Drought season here?

From Aug to Oct they come back here from Nov. Although even now the disease is there

Oltigana?

This one only in cattle.

Signs

This one comes when they take animals to a swamp where it is a wet place and when the animal comes home, we notice excessive salivation and the dung is insect infested too. After it rains during the drought season, we take the animals to the farms and that is where they get oltigana.

Seasons?

Not all the time but when we take animals there then after it rains we start noticing the disease.

To people?

No even if we eat the meat or drink the milk

Olorobi

Shoats and cattle

Signs?

Cracks on the mouth and hooves, not able to eat, doesn’t leave the home.

Seasons?

This one is common during the rainy season

To people?

After the animals recover people get olorobi but there is a difference between a common flu and the one from animals.

How to differentiate between the two?

When cows have olorobi and you have olorobi then you know it is from animals but if you get when the cows don’t have it then it is not from cows. When the animals recover then people in the area get ill from olorobi so we associate it with animal olorobi.

Signs in people?

Running nose, shivering, eyes ache, body weakness, headache

Do you call it olorobi when it is in people too?

Also, olorobi

Mode of transmission?

From milk.

And if you boil the milk?

Even when we boil the milk people still get it.

Treatment for olorobi?

We purchase medicine from chemist such as centrizin, Panadol, celestamine. We also use herbs like oremit, olng’osua, suguroi, enararua and enatatua for kids. We boil some while others we just put into water. Enatatua is for very young children and it is very bitter the child has to be forced to take it.

Please explain to me how you decide if to use conventional drugs or herbs?

We start with herbs to induce vomiting (olodua (nyongo)) and sometimes this works and one doesn’t need to go to the hospital. However, if I don’t get the herbs or the disease is severe then I go to the hospital. If I take the medicine and I don’t feel well sometimes I will buy cetrizin especially if I am sneezing.

Do you ever use both at the same time?

No, we don’t mix herbs and conventional medicines. We use one and if we don’t get well, we go to the other option. For example, after using hospital medicine then I take “oremit” so I either start with herbs or hospital medicine and if no recovery I go to the other option. Sometimes “oremit” works and sometimes the hospital medicine also cure the illness.

Enadomonyet?

Shoats only

Signs?

Bloody diarrhea

Treatment?

Teramycine

To people?

No

Enadogulak?

This one is the same as enadomonyet only in cattle. Bloody urine in cattle but bloody diarrhea in shoats. Eladogurum is in calves which is bloody diarrhea so we apply kerosene on the anus or a hot iron and burn the tail and it recovers. We burn because that is the right medicine.

Treatment?

Teramycine and the animal usually recovers.

To people?

No

Nunuk?

Cattle only

Signs?

The cow gets it and it is like olorobi then you put ash on the back and then inject and it recovers. When an animal is sick with this disease we don’t let the animal drink water and the animal doesn’t urinate or move. The ash helps it to get better it can stay for three days without walking. The ash helps to get the urine out and the sun burns it and the it is able to urinate.

Seasons?

This one is when it is cold

To people?

No, it doesn’t

Olodua

Shoats and cattle

Signs?

(Long discussion) You only know once you slaughter not before when you see the bile is too much. Olodua is bile then once you see you treat the other animals. But sometimes the animal will have had black diarrhea.

Treatment

This one has a specific medicine for it which the govt has brought. Sometimes we also call the doctor to come treat the animal.

Seasons it is seen mainly?

It is periodic.

To people?

No

Kububo?

Cattle only

Signs

The legs become weak so this one has no cure. The animal dies unless you sell the animal or slaughter it and eat. Most of the animals if sick or die from an illness we slaughter, skin and eat the meat. It is only in the case of enariri that we don’t eat the meat and for olmillo the head is thrown away.

To people?

No

Enarogua?

Only in goats

Signs?

It is like pneumonia and the big ones are the ones that get sick because the fat in the chest causes the congestion. This one has no treatment so you slaughter and eat instead of waiting for it to die and there is no treatment anyway.

Seasons?

During the rainy season because that is when animals are fat.

To people?

No

Ororashrash?

Cattle

Signs?

This one is olorobi because when the animal is not able to walk that is this disease…(long discussion). There is a leaf called ororashrash and when animals step on the leaf then we say it has that disease and only happens during the rainy season so that is when animals step on it and are unable to walk.

Kileny?

This one is for kids.

Signs?

The hind limbs become weak because of a back problem.

Seasons?

All the time these days. In the past it was not common.

Treatment?

Has no cure so we slaughter

To people?

No

So the only zoonotic diseases are olorobi and eriri?

Yes

Which one worse?

Eriri is way worse because you don’t eat the meat or the milk and you have to throw the animal away. There is also a condition whereby the milk becomes bloody after the animals have crossed over certain ants.

Eriri in people?

Rashes and people recover. It can be transmitted to people from animals if you drink the milk

Eriri treatment?

Hospital because it is bad and there is no treatment at home.

Of the diseases you have mentioned which ones are the worst?

Olmillo and olekipei because animals don’t recover in the case of olmillo and for olekipei the animal becomes very sick. These diseases are also very common.

Nguruya olchaget?

This one is from wild animals and has no cure that we know of.

Any other disease from wild animals?

None

Please tell me about the consumption of raw milk?

We no longer take raw milk but the boys drink when they take animals to pasture.

Why did you stop taking raw milk?

There are many diseases these days from milk.

Like which ones?

Eriri, olorobi and another one called brucellosis

Is Brucellosis (engeya gule) in animals too?

No. Only in people.

How is transmitted to people then?

The name “ugonjwa was maziwa” is just a name the disease has been given it has nothing to do with milk. It is like “ugonjwa wa maji” (typhoid) or “ugonjwa wa sukari” (diabetes). It is just a name and has nothing to do with milk but also when we go to the hospital, they tell us it is from milk. When you are on treatment the doctors ask you to stop taking milk even in tea. So maybe it is from milk that is why they ask us not to take milk alongside the drugs (animated discussion). Brucellosis is caused by milk and that is why we boil the milk.

Raw blood?

Sometimes in the past it used to happen even now some do take but not many people do. Older men and warriors (warani) do take it when they go to the forest to celebrate. They drink raw blood but here at home we don’t.

Why not here at home?

We now go to church and the church tells us not to. Those who are not saved take blood but the bible tells us not to but we would still take if the church allowed us (laughter) it is very tasty we still would drink it. It is very tasty fresh and warm after slaughter.

Why do the men still take raw blood?

They don’t pay attention to things like what the church says or diseases.

Any disease from raw blood??

No, it makes us very strong and keeps you warm you don’t even feel cold on a rainy day! We obey what the church has said though. We used to put it in meat though but not now because of the church we don’t not because we are afraid of a specific disease. When a woman gives birth, an animal is shot and the lady is given the blood to drink or after circumcision to replenish lost blood and to regain health.

Assisting in parturition, tell me how you do it?

We use our bare hands

Disease from this practice?

None at all

Residing with livestock?

Yes we let the kids sleep in the room next to us and there is no problem we do it so that the kid doesn’t suckle at night and we can milk in the morning.

Any health issues?

None other than bed bugs and sneezing from allergy but we still keep them so I can milk in the morning. When able to I build a shed and if they are many but if a few I keep them in the house with me.

Any other zoonotic disease?

None other than olorobi and eriri.

And Brucellosis?

Yes

Has anyone here ever had brucellosis?

I have

What were the signs?

Tiredness, swelling, not able to walk, headache, no appetitie , don’t want noise, joint pains. When I went to the hospital, they said it was brucellosis and I got 21 injections. I used the medication and recovered completely. So after the drugs I am now convinced but at first I had not believed because I don’t have cows so I don’t have milk so I concluded they had lacked a name so they called it milk disease. I dont have milk I even went to several hospitals and they all said I had B so now I settled and got the treatment but I still don’t belie it is from milk, that is just a name.

Did you take any herbs?

None I just went to the hospital

Would you like more information?

Yes, we would like to know more like what causes brucellosis and olmillo and oltigana we want to know all the diseases and how to treat them.

Best way to teach?

Group sessions are best because some don’t know how to read and we can explain better to each other when together in the training session. And everybody will have knowledge and we can tell others.

Any question?

None

END
