# Supplementary material for: Prioritization of livestock diseases by pastoralists in Oloitoktok Sub County, Kajiado County, Kenya
Source: PLoS One. 2023 Jul 12;18(7):e0287456. doi: 10.1371/journal.pone.0287456 (PMC10337939; doi:10.1371/journal.pone.0287456)
Supplement: S1 Data — (ZIP) [file pone.0287456.s001.zip › Oloitoktok transciptions/FGD W 2.docx]

**FGD**

I: Common livestock diseases?

P: Olorobi

Eng’ororo

Nunuk

Olekipei

Oltigana

Engeya Geri

Engeya nerogua

Engeya orogony’

Olorobi signs?

Affects shoats and cattle. At fIrst you see “isuuro” (dullness and doesn’t eat) and then you see salivation and lameness and wounds in the hooves.

Any other signs?

Pause. When you inject you notice it was FMD because the animal becomes more severe after tetracycline.

When rainy or dry season?

When it is raining. During the rainy season because of the rain and the mud but during the dry season there is no mud so they are not affected.

Which months are rainy?

Oct-Dec and also Feb to April

Can it be transmitted to people?

A resounding yes when animals have olorobi people also have olorobi

Signs in people?

Shivering, fever, vomiting, headache. We call it olorobi even in people.

Any other signs in people?

Chapped lips and diarrhea. And these signs come when animals are sick

Eng’ororo?

In cattle only

Signs?

Cattle bleeds from the ears and there is no milk let down and it takes time before you know it is sick. The milk reduces though so that is how you know. Also the color changes meaning the animal has rough hair coat eg from brown to very brown.

Is it transmitted from animals to people?

No

Nunuk?

Cattle are unable to walk, “isuuro”. Only in cattle. We leave it in the sun and pour ash on its back because we believe it cures it. It stands in the sun until evening and after the ash treatment and it urinates, we believe it has recovered. We also spray it with the “dip medicine” and it recovers.

To people?

No although we are not sure because sometimes our legs hurt so we don’t know if it is nunuk (laugher). You feel backache and stomachache just like the animal.

Oltigana?

This is in cattle only

Signs?

A foul smell when you go to milk the animals, it excretes a lot of cow dung and diarrhea. Also, the meat turns yellow and it is very delicious meat all the same when slaughtered (laughter). Those animals that have died from ECF we don’t eat anymore but when we used to, they were very tasty. We don’t eat carcasses because we know they can transmit disease.

To people?

Yes, you get diarrhea and hyperacidity. The acidity is high.

Enariri?

Cattle, shoats and also people

Signs in animals?

Rashes on the coat, that is the only sign. Rashes `and it will be lethargic. We just wash with dip until it recovers. It takes time to recover.

To people?

Yes, it is transmitted to people and we call it eriri.

Signs?

When the animal recovers people get it. And it happens when you eat meat from the animal so we don’t eat the meat. When you get the disease, you drink fat from mutton which makes the rashes come out on the skin and then the disease clears. In children it also comes with fever, chest pains and the rashes. In children they get chest congestion and also in some adults. In children they also have thick mucus. We fear the disease a lot because it gets even to the eyes.

Engeya oluguny?

Mainly shoats but also cattle.

Signs?

The animal just runs, is restless and can run to chyulu hills and is relentless in its run. We don’t have medicine for this disease. As the disease starts the animal faces down and shakes its head for like an hour and then you know the disease is in and also circling so you tie it down and it “cries” a lot and the only solution is to slaughter it.

Has this disease always been here?

It is fairly recent…. the five to last seven years while the other diseases have been here for a while. And it has no cure so you sell it to the butchers

To people?

No and we are praying it doesn’t and it generally infects healthy and fat animals.

Olekipei?

All but shoats mainly and more so goats.

Signs?

A lot of mucous, coughing, it is like pneumonia because the animal coughs a lot and is in pain and it doesn’t eat or lie down. There is a lot of panting and it drinks a lot of water. It takes time and the animal loses a lot of weight and has diarrhoea. It kills goats fast and in large numbers.

To people?

Yes…(doubtfully). It is pneumonia in people. People cough and it is not olekipei but people get pneumonia.

Will it be from animals?

Maybe it can but sometimes you can find that you have similar signs to the goat (laughter)

Engeya Enarogua?

An animal just dies suddenly with no other signs the previous night…”enterotoximeia” and it affects fat animals. Mainly shoats and you’d have seen no signs previously.

To people?

There is sudden death in people but not associated with this disease.

Any other livestock diseases?

Olodua in goats (it is PPR)

Signs of Olodua?

The animal dies and “nyongo ni kubwa” and also there are no signs before it dies.

To people?

No

Which ones dry seaseon?

Eng’ororo (tryps) it is always there but mainly when they go to the mountains and interact with other animals. It is only in cattle.

Signs?

It is caused by a big blue fly not a regular fly.

To people?

No

Do livestock interact with wild animals?

Yes. A lot with elephants, zebras, antelopes, hyenas, buffaloes, wildebeest, giraffe

Any danger to health?

Yes livestock are preyed on by the wild animals and also transmit diseases like olchang’et . (MCF) which is caused by wildebeests maybe through water or when wildebeests give birth ….we are not sure.

Signs in livestock?

Patches on the body. We don’t really know. It happens when animals go to the Chyulu hills and we women don’t go there so were not sure.

How do diseases move from wildlife to livestock?

We know a disease that causes patches in animals but we don’t know how it is transmitted.

Any diseases which can be transmitted through un boiled milk?

Yes, olorobi and eriri when you take unboiled milk you get the pox (eriri). You can also vomit and have diarrhea from raw milk as well as worms.

Who mainly takes raw milk?

Men do mainly.

Why men?

They don’t believe it causes disease and they feel that it is too much work to boil the milk. They drink and they don’t die from it. They don’t get sick because they don’t believe it will be harmful. Children are given boiled milk but colostrum which the children take is not boiled.

Why are they given colostrum?

It makes them have diarrhea and thus eliminates disease. Colostrum also makes the child strong and healthy. And the child cannot get pneumonia. Adults don’t take it because they don’t need it as much as kids do but in the past adults would drink it. Some people also don’t like boiled milk so they take raw milk so we divide the milk into two and boil some and leave some raw. There are people who like raw milk which they put in a container which gives the milk a certain pleasant smell and they take that.

Other than olorobi and diarrhoea and olodua any other disease from raw milk?

No

Is raw blood consumed?

Some still do take it. There is meat that people mix with blood and they eat. There are people who eat raw blood or meat mixed with raw blood but those who go to church do not. They believe that as Christians they shouldn’t

Any possible diseases from this practice?

No, none. The raw blood makes one very strong. In the past they mixed porridge with blood or even fermented milk with blood. This is not very common nowadays though.

Can assisting with parturition lead to any disease?

No, disease. One just does it and then washes their hands and sometimes you don’t wash hands. Sometimes you even give the kid mouth to mouth resuscitation if the kid is weak and we have not experienced any disease. No point in wearing gloves but if you want to you could. We only wear when helping women to deliver. Human blood is different from that of animals meaning that one can get disease from a person but not an animal.

Residing with livestock?

Yes, happens a lot. The kids are kept in the houses.

Why?

Because of cold and rain, we don’t want the kids to be rained on. They sleep right next to us

Any disease?

No none. They sneeze right next to us but the only issue is bed bugs. And also there is sneezing, those who sneeze are the ones that don’t love animals (laughter). There is no disease, all of us sleep with livestock.

Meat from livestock any disease risk?

(A long discussion) …just allergies nothing else you cannot get sick from eating meat.

Treatment seeking behavior for olorobi?

You take “oremit”, “orengurue”, and go to the hospital. We also take the roots of the “osoit” tree boil then take. That one causes you to have diarrhea and the disease is eliminated. For kids you give them “anararua” and “enemelue” those are for kids.

How do you decide if to use herbs or go to a hospital?

You can start with either and if not well you take the other.

What determines that?

You just decide, all of us do that. It also depends on what is accessible eg herbs because the hospital is far. Kids also can take herbs first.

When do you take kids to hospital?

When a child is severely ill; very feverish, lethargic that is when we take the child to the hospital but sometimes, they recover with herbs.

Buying medicines from the chemist?

Yes, we do. Drugs such as brufen, paracetamol, piriton and amozyl as well as sona moja.

Why buy medicine from the chemist and not go to hospital?

There are no good facilities or doctors here so sometimes we prefer to buy from the chemist. Sometimes there is a doctor but no medication. We go to the hospital when severely ill which is when I cannot get up and lethargic and unable to eat too so if I can still function then I take herbs and I am ok. We believe that the herbs are effective. We don’t like hospitals a lot we believe herbs help us. Some even take time before going to the hospital they just use herbs.

For olorobi and olodua which one worst?

Olorobi is worse

Why?

Makes you have a headache, joint pains and no appetite

Brucellosis, ever heard?

We have heard of it and some have even received the injections. Two said they have had brucellosis and given 21 injections each.

Signs of Brucellosis?

I couldn’t do anything on my own because my legs were weak, for me it was the same and in hospital they tested because I was hurting everywhere. We don’t know what causes it but in hospital they tell us not to take the milk and meat.

Why do they ask you not to take meat and milk?

For the 21 days of treatment ones shouldn’t take meat and milk. Doctors believe it comes from milk which may be true but we don’t think so. Maybe the milk weakens the drugs so they tell us to avoid milk so that the drugs can be effective.

Why is it called the “disease of milk”?

We just hear it is from milk but we don’t believe it. We cannot stop taking milk so we cannot believe it is from milk.

Is brucellosis found in livestock?

No, it doesn’t infect animals and it is only in people

Have you ever heard of a disease called anthrax?

Yes, we have and we burn the carcass

In people?

It can affect people that is why we burn the carcass

Rabies?

We know about it. We have seen rabid dogs and the rabid dogs bite kids who are taken for treatment.

Would you like to know more about zoonotic diseases?

Yes, we would like to know how toy are transmitted especially brucellosis so we know how to prevent it and how it is from animals.

Best way to educate you?

A big meeting should be held and then we are taught. It can be done in churches but not all people go to church so a meeting in the village would be good.

Any questions?

Yes. Why have you come to ask us these questions?

I explain on the goal of this research and the sensitization mechanism planned for zoonotic diseases in this area.

**END**
