# Supplementary material for: Prioritization of livestock diseases by pastoralists in Oloitoktok Sub County, Kajiado County, Kenya
Source: PLoS One. 2023 Jul 12;18(7):e0287456. doi: 10.1371/journal.pone.0287456 (PMC10337939; doi:10.1371/journal.pone.0287456)
Supplement: S1 Data — (ZIP) [file pone.0287456.s001.zip › Oloitoktok transciptions/IDI F 11.docx]

**IDI**

I: How long have you kept livestock?

P: Since I was born; I have grown up in a livestock keeping family.

What livestock do you keep now?

Sheep and cattle and chicken also.

Why do you keep livestock?

They help me in life because when I give birth, I eat meat and I also sell the livestock to pay school fees and to buy food. I also keep livestock for milk and so I must always have the livestock.

Where do you graze your animals?

During drought I take them to the fields after people have harvested their produce. When it is not drought time the animals go to the wild areas “porini”.

Do you ever take them to Amboseli National Park?

No, we don’t. The graze in the areas surrounding the park.

Do livestock interact with wild animals?

Yes such as wildebeest, giraffe, antelope.

Are livestock taken to Tanzania for pasture?

No.

What are some of the challenges you face as livestock keepers?

There are diseases like Olekipei and enariri and olmillo.

Olekipei enariri and olmillo, any other diseases?

Nunuk also. Only those ones.

Please tell me about Olekipei?

This disease affects Shoats and the signs are coughing and then the lungs are so congested until it cannot cough anymore but once we treat with teramycin it gets well.

Can it be transmitted to people?

No.

What about Eriri?

It affects cattle.

What are the Signs?

The skin of the animal will have some nodules. That is the sign.

What treatment do you use for eriri?

Teramycin and penicillin.

Is it a big challenge?

Sometimes they die but sometimes they recover.

Can it be transmitted to people?

Yes, there is chicken pox which affects children and we call it “masanduku” and it is from animals.

Does it affect only children?

Yes.

How is it transmitted?

Through milk.

Please tell me more about Olmillo?

Olmillo is a disease of Sheep and Goats but mainly goats.

What are the Signs?

The animal circles and “goes mad”. It doesn’t recover so it can only be slaughtered.

Is it transmissible to people?

No, it cannot be transmitted to people.

What about Nunuk, which animals does it affect?

It is only in cattle.

What are its signs?

The animal is unable to walk so it is stationary.

Any other sign?

None but recovery is common with this disease. We use teramycin and apply ash on the back because that is the medicine. The animal can walk once you put the ash on its back.

Is it transmissible to people?

No.

Any other livestock disease?

None.

Can wild animals transmit disease to livestock?

Yes, for wildebeests they transmit “nguruya olchang’et” (MCF) and you find that where they have urinated if the livestock eat that grass they get the disease.

MCF Signs?

The animal is sick in the whole body.

Which of the diseases you mentioned are the biggest challenge?

Olmillo is the worst because the naimals never respond to treatment and always die. Followed by MCF because when it rains the cows get the disease because it coincides with the calving season for many wild animals. We use teramycin to try and treat the disease and it is not always successful.

Any other?

Those two.

Do you know any other zoonotic diseases?

No, I don’t.

Do you take raw or boiled milk?

We use boiled milk to avoid olmillo and MCF and nunuk. Nunuk makes a cow not even urinate so it is a bad disease. We boil milk to just avoid any possible disease but we don’t know which diseases.

Are there some who don’t boil their milk?

I think nowadays all the people boil their milk before drinking it.

Is raw blood consumed?

In the past people would drink but these days no unless when men go to the wild areas they drink and I don’t know why they do.

Why have [people stopped?

I don’t know maybe it is because they now go to the church and it is a prohibited practice.

Any disease from raw blood consumption?

I have never seen or heard.

Assisting parturition with bare hands, can it lead to any illness?

We do it all the time, assist the animal and then wash our hands.

Any risk for disease from this?

None, it is ok to do that.

Residing with animals?

Doesn’t happen but when you keep the kids in the house you sneeze because of the smell so we avoid it. We build sheds outside.

Any health challenge or disease from this?

None.

What about milking is there any risk for disease from this practice?

None, only the consumption of raw milk.

Ever heard of brucellosis?

Yes, some people go to the hospital and are told they have brucellosis. I have never gotten it but I know people are told they have it and receive 21 injections.

Why is it called milk disease?

I don’t know maybe we drink the milk and then the cow is sick (has homa) so we get it.

Is it in livestock?

I don’t know

Anthrax?

I have never heard

Rabies?

That one is there.

Tell me more?

You see a rabid dog and when it starts barking and biting someone they are taken to hospital because the dog has rabies.

How to prevent it?

We kill the dog and go to the hospital.

No other zoonoses?

None.

How do you identify a sick animal?

It doesn’t graze and there is no milk let down so I know it is sick.

What do you do?

We treat it.

Do you call doctor?

No, we don’t unless they just come. We just inject with the drugs we buy in the shops.

Would you like more education on zoonotic diseases? What would you like to know?

How to treat these animals when they have these diseases and treatment for Olmillo. I would like to know the treatment for these diseases. I want to know if there are other medication because I only use teramycin and penicillin.

What would you like to know about zoonotic diseases?

The transmission pathway from animals to humans.

Best ways to pass this information?

I would like this done in a group so that all of us know what causes these diseases and how to prevent them.

Do you have any questions?

None

END
