# Supplementary material for: Prioritization of livestock diseases by pastoralists in Oloitoktok Sub County, Kajiado County, Kenya
Source: PLoS One. 2023 Jul 12;18(7):e0287456. doi: 10.1371/journal.pone.0287456 (PMC10337939; doi:10.1371/journal.pone.0287456)
Supplement: S1 Data — (ZIP) [file pone.0287456.s001.zip › Oloitoktok transciptions/KII 7.docx]

**KII**

What is your role here as an elder?

I am a farmer of maize and keep livestock as well. I am also an elder in the office of the chief.

What are the challenges you face as pastoralists?

Diseases and attacks from wild animals because we live close to the Amboseli park. This area is right next to the park and livestock and wild animals graze in this area.

Which wild animals are a challenge?

Lions and hyenas and the latter know they are not being killed so they come to the homesteads but lions not as much. Lions are rarer but hyenas are very common.

Where do you take your animals for grazing?

We go to the wild areas and during drought we also zero graze the animals with maize stalks When there is no drought, we take animals to the wild areas and that includes sheep and goats.

Do herders get attacked by wild animals or only the livestock?

Mainly the wild animals during herding don’t attack livestock but they attack when the herdsmen are not nearby. Sometimes when an animal wants to eat a livestock they hide and don’t do it when herders are close.

Do you take animals to Tanzania for pasture?

It is rare and only when there is severe draught.

Please tell me the common livestock diseases?

FMD which is “homa ya ngombe” or “olorobi”

Signs of FMD?

Swollen tongue, salivating and swellings in the hooves.

How common is it?

FMD is very common and in the past, it was only in the wet season but now it is presents even in the dry season. Even recently in Jan and Feb it was there. Affects even shoats.

Any other?

There also is nunuk where the animal becomes weak and cannot walk. It just lies down. In cattle we call it nunuk but it is also pneumonia in humans.

Is it zoonotic?

It is common when the vegetation is a lot not all the time.

Signs of nunuk?

The animal coughs and walks slowly. Affects only cattle.

Any other diseases?

Enariri…iriri…”ugonjwa ya Ngozi”. People used to have an injection which they believed would prevent this disease. Affects all livestock.

Kindly tell me which seasons specific diseases are common?

Diseases are there all the time and, in the past, they would quarantine the sick animals. There was a time like eriri was common but it isn’t now due to vaccinations.

Any other disease?

“Enguruya olchaget” (MCF). This one occurs when wildebeests are giving birth in June and causes cattle to be blind. They start to give birth in April and the animals are taking water in the same areas so they get contaminated from all the birth materials in the pasture and the water and signs of the disease in livestock are seen from June.

Signs?

Rough hair coat, dry mouth, a wailing sound from the cattle and teary eyes and they don’t respond to the injections we use.

Steps when an animal is sick?

We first restrict the movement of the animal and use common drugs like teramycin and keep it home and not let it go to pasture because it may be unable to come back home.

If no recovery?

We sell the animal.

Do you know about zoonotic diseases?

I don’t know any. I could be sick and it comes from meat and milk but I don’t know.

Do you know about Anthrax?

It is not common here.

What about Rabies?

I have heard of this one.

Tell me more about it?

It is there and once someone has been bitten by a dog whether rabid or not, we rush the person to the hospital.

Have you ever heard of brucellosis?

I have heard of it but I don’t know if it is from milk or it is just the name “milk disease” it has been given.

Can the consumption of meat and milk and close proximity to livestock cause any disease to humans from those animals?

I don’t think that being with animals can cause disease. In the past we would take raw meat and raw milk but now we don’t.

Why are you now boiling milk and taking well cooked meat?

Because of interaction with other communities who demand that they want boiled milk and well-cooked meat so even I adapted to this way of life but not because of fear of disease. I have just adapted because in the past we would consume these things and there was no disease.

Are the livestock diseases you mentioned earlier zoonotic? FMD, Nunuk, Iriri and MCF?

Right now there is “homa ya ngombe” and in that time people have homa too. Also, Eriri you find some people have it but I don’t know if it was transmitted from livestock.

Tell me more?

For us we say olorobi in both livestock and humans and Eriri is when livestock are sick with rashes and skin disease for people is also called iriri.

Olorobi signs in people?

Coughing, shivering and chest pain.

Kindly tell me about the treatment seeking behavior here?

In the past people used herbs but nowadays most go to the hospital.

Please tell me why hospitals are now more utilised?

In the past hospitals were far but now they are close and the doctors know what medication to give the patient.

Please explain how Nunuk in animals is associated to pneumonia in humans?

For nunuk the animal shivers and for humans it is the same; the person will be cold and shivering and that is pneumonia. The person will be looking for warmth.

Do you have any questions?

I would like to know if there is a possibility for vaccination for all these diseases?

I respond on goals of this research and prioritization. And the steps forward.

*Elder takes us around the village. Homes, watering areas, grazing areas.*

END
