# Supplementary material for: Prioritization of livestock diseases by pastoralists in Oloitoktok Sub County, Kajiado County, Kenya
Source: PLoS One. 2023 Jul 12;18(7):e0287456. doi: 10.1371/journal.pone.0287456 (PMC10337939; doi:10.1371/journal.pone.0287456)
Supplement: S1 Data — (ZIP) [file pone.0287456.s001.zip › Oloitoktok transciptions/IDI F 2.docx]

**IDI**

I: How long have you kept livestock?

P: I was born in a home with livestock and I keep cattle sheep and goats. I also keep chicken. Goats and cows are the majority.

Why livestock?

For us the Maasai livestock are our bank because to get food we sell livestock and we also educate our kids through the money we get from livestock. We have just started farming; livestock have been our bank and lately I grow some maize and beans on a small scale.

Which areas do you take your animals for grazing?

We graze in the group ranch where we have not grown crops so we take them to Amboseli and they graze together with wild animals. We don’t take them anywhere else but during the drought season we zero graze the animals by feeding them residues from maize . We sometimes take them to chyulu ,mombassa and taveta. Most of the cows go to those areas but some remain at home like five or six ;those are the ones that we zero graze.

When is drought season here?

Drought is in Sep-Nov. It rains in Jan.

Are you allowed into Amboseli?

Yes

Which wild animals do livestock interact with?

Lions and elephants and many other animals. But these are the dangerous ones as elephants kill herders and lions prey on livestock. It is a problem but we don’t have a choice. We just evade the elephants as much as we can.

What other wild animals?

Yes, buffaloes and zebras and others.

Do you take livestock to Tanzania?

Yes, we once did but then our animals were apprehended in Tanzania and so we don’t go anymore. We are now afraid to go.

Challenges as pastoralists?

Wild animals kill livestock and also animals mixing in transit moving to other areas and this causes them to infect each other with diseases so we just manage and treat as much as we can but sometimes, they die. This happens in the grazing lands and there are diseases like rashes which kids also get it when they drink milk from diseased animals.

Other challenges?

Yes, drought is a problem and animals become emaciated and then the price in the market is very poor when animals are thin. Also, wild animals killing herders.

Common livestock diseases?

We have CCPP which affects the lungs and it kills animals very fast. It affects goats and is highly contagious. Then rashes and also a cow just appearing ill so we pour ash on its body.

Signs of CCPP?

It is a coughing disease and when the animal dies and is skinned the lungs are joined together and it has no cure and is very contagious within the locality.

Please tell me about Eriri?

This one is contagious in different homesteads too and we don’t consume the meat. We bury the carcass even dogs cannot eat the meat because they die. Signs are shivering and salivating and then the following day it has rashes all over the body so we pour ash on the back and don’t give the animal water for three days and inject teramycycin.

Then in goats we have olmillo which is when an animal circles and screams and we slaughter because it has no cure so we slaughter and we eat the meat. You just slaughter. It mainly affects shoats.. This is a recent disease because in the past it was not there.

Other diseases?

None other than these and nunuk where the animal looks like a wilted crop and shivers so you pour ash on the back and if it dies it can be eaten.

Any other?

None

FMD?

An animal salivates and has mucus and is hot inside the mouth and also the hooves come out so it is unable to walk.

Can FMD be transmitted to people?

Yes, because when you milk a cow with FMD and people drink the milk or tea then they get FMD.

Signs in humans?

The child gets a fever and sweats a lot but when you take the child to hospital, they get medication and they are well again. Even adults get it so we don’t milk a cow with FMD. In people we call the disease olorobi.

Kindly tell me more about Nunuk?

This one is not in people.

Olekipei?

This one is in people because many people have TB and with CCPP the lungs “zimeshikana” like someone in TB whose lungs are sick so it is the same disease.

Eriri?

This one is dangerous so when the animal dies, we discard the carcass by burying it. Even dogs should not eat the meat because they also die. This one can be transmitted to people

So CCPP, FMD and eriri are the ones that can be transmitted to people?

Yes

Signs of eriri in people?

In people a child has fever and coughing so we take the thorns from the olng’otua and “marbait” tree and we put in a pot and cover and let it boil then we let the child stay under the hot steam and they shower in the same water and the pox comes out all over the body by the next day. Then we take the child to hospital for the injection and they recover but if we take them to the hospital before the herbal treatment and they are given the injection then the child dies. The pox if it doesn’t come out to the skin manifests in the inner organs and that is what kills the child. In animals eriri manifests with pox

Any other zoonoses?

Pause. Only CCPP, FMD and eriri not nunuk.

Which ones are a major Priority?

Eriri and CCPP because these ones kill. Olorobi doesn’t.

What treatment do you use for adults with eriri?

We do the same thing that I described for children and they steam under it in the evening and shower with that same water and after the pox comes out then they go to the hospital and they receive the injections and they recover.

Why do you say CCPP is a big priority?

Because it is TB and the person is not strong again after contracting it and people die also.

FMD?

FMD is fever and easily treatable

Any other Zoonotic diseases?

None other.

Brucellosis?

I don’t know about this one.

Do you consume your milk raw milk or boiled?

These days we boil it for a while even for use in tea we always boil because there are many diseases. In the past children would follow us in the kraal with cups and we would give them the raw milk as soon as we milked but now, we boil because of the many diseases like FMD, eriri and CBPP. People now know so it is not like in the past.

Are there any who take raw milk?

None

Herders?

These ones take raw milk they have no time to boil milk. But us here at home we always boil.

Raw blood consumption?

In the past we would even mix with meat or take it after it has curdled and we would eat it but nowadays this doesn’t happen. People know it is not safe. Even the young men are not eating blood.

Assisting in parturition, do you wear gloves?

We don’t wear gloves only if we are assisting women in childbirth.

Are there any risks for disease from this practice?

I don’t know but these days animals have become like people when a kid is born it comes covered in a thin film which one must tear open. And sometimes they are born hind legs first or sometimes we have to tear and suture. So it is different from how it used to be in the past. Sometimes the births are difficult so we have to cut the calf in pieces to remove it dead. We wear gloves when assisting women to deliver.

Why for people?

There are many diseases and we have to protect ourselves. These days we don’t help women to deliver without gloves, we borrow them from clinicians then we keep them at home. And use them as need arises. But for livestock we don’t use gloves. I don’t think there is any issue, I don’t know any disease from that.

Residing with livestock?

Yes, in the past we would do that. The kids and calves we would keep with us in the house. However, these days people want to be clean we don’t want dung and flies in their houses. In the past we lived with them even in the kitchen but now we don’t want that dirt. Dirt causes disease like flies which transmit diseases.

Wild and domestic animals interacting, what are the risks?

Yes, MCF is from buffaloes and when they scratch on trees and then livestock do the same, they catch this disease called (inkuruya olchaget) and it has no cure.

Signs?

The signs are a sore spot on the animal. So, we put some cow dung on the spot regularly and eventually because it has no cure a wound eventually comes out. So that is just a home remedy that we try out. Sometimes it works.

Any other diseases from this interaction?

None only that one.

Do you use herbs before going to the hospital?

We start with herbs and if not well then, we go to the hospital because if you go to the hospital first then you cannot use herbs. We do this for all even children, there are some signs I see in my child and then I just use some herbs.

How do you decide it is time to go to the hospital?

Because I know the signs of the different diseases.

Would like further information on zoonotic diseases? What exactly?

I would like to know more so that I know how to protect myself.

For zoonotic diseases?

For eriri and FMD I know how to protect myself but not for CCPP.

Best way to educate the community here?

Churches would be good.

Even for men?

Yes, even men come to church.

Any questions?

No, none

END
