# Supplementary material for: Prioritization of livestock diseases by pastoralists in Oloitoktok Sub County, Kajiado County, Kenya
Source: PLoS One. 2023 Jul 12;18(7):e0287456. doi: 10.1371/journal.pone.0287456 (PMC10337939; doi:10.1371/journal.pone.0287456)
Supplement: S1 Data — (ZIP) [file pone.0287456.s001.zip › Oloitoktok transciptions/IDI F 12.docx]

**IDI**

I: How long have you kept livestock?

P: I was born in a livestock keeping home.

Which animals do you keep?

I keep goats, sheep, cattle and poultry.

Why do you keep these animals?

They are very beneficial because when my children need school fees, I sell some and take them to school. For poultry we eat the meat and eggs and also sell them.

What are the grazing areas for your animals?

We go to the area where is no park; the other side of the park because we border the park. During the drought season we go to Risa and Marba on the other side of the park and namelok.

Why Risa?

It rains a lot in Risa and it is in Entonet ward.

Do you ever take animals to Tanzania for pasture?

We don’t go to Tanzania but we get to the border but we stay on the Kenyan side.

When is it drought season here?

Aug until it rains. In Sep the signs of rain start and it rains in Nov.

What are some of the challenges you face as pastoralists?

We have drought and diseases. Diseases like MCF and olorobi. Like now many animals have it and it affects their hooves and mouth. Also, eriri and enkororo and olmogo where the animal gets a big wound that does not heal and the wound increases in size until it heals on its own. Olmogo you don’t see the signs until it drinks water and dies. So when it has elmogo you deny it water. Olmillo is for shoats also, Kileny. With Kileny (neuromaicroplaragia) the hind limbs become weak and it mostly affects the kids. It is among the kids. Also, elodomonyet but we don’t have it here.

What are the signs of olorobi?

It affects the hooves and the mouth.

What seasons do you most encounter olorobi?

During the rainy season.

Can it be transmitted to people?

People get it and it doesn’t have to be from cows but sometime s people say it is from the milk from the sick cows.

What are the signs of olorobi in animals?

Shivering, fever, sometimes some go to hospital and others take oremit which is a herb.

Hpw d they decide?

If you go to hosp and not getting well you stip the hospi medi and take herbs.

What about olmogo, is it transmitted to people?

Not to people but we sometimes associate it with skin disease in people.

Signs od olmogo in animals?

A wound that is oozing blood .

Any other symptoms?

Olmogo is just a round wound oozing blood so in the morning we take fresh cow dung apply on the wound, the animal scratches the wound and it heals.

Why cow dung?

You use cow dung to prevent birds from licking the wound. It is a covering then in the evening we apply salt or the powder from “capsules”. These are the capsules one gets after the tetanus injection. We keep the capsule to use for this condition.

Enkororo?

Does not affect people but we don’t eat the meat especially If the animal had been sick for a long time.

What are the signs?

You can know when an animal drinks water then it comes home and dies. You see all the legs are swollen so if they see that they prevent it from taking water but if they don’t see then it drinks water and dies.

Olmillo?

This one is only for shoats. “Nguruya nchang’et” is in cattle which is MCF when an animal drinks stagnant water that wild animals also use there is lacrimation, salivation and circling like heartwater disease for the cows. The animal starts circling for olmillo and it isolates itself so we don’t allow it to go far. Circles and goes far. We also have olmillo in people where you feel dizzy but it is not from animals. Olmillo means dizziness.

Kileny?

Affects the hind limbs.

Is it transmitted to people?

No.

Other livestock diseases?

None.

Which of these diseases are common during the rainy season?

olorobi, Olmogo, Olmilo and Engororo are there all the time, Kileny is very common here.

Can MCF be transmitted to people?

No.

Any other zoonotic diseases?

None. Olorobi you find kids get sick so people say it is olorobi from cows.

Are there risks for disease when wild animals and livestock interact?

Yes, children are attacked by elephants and I know also that MCF which is transmitted by wildebeests.

Do people take raw milk?

Laughs…we don’t take un boiled milk but sometimes we give the raw milk to the little children because sometimes they are so hungry and cry asking for milk. We also give colostrum to the children.

Any diseases from raw milk?

None.

Why do you boil the milk?

To kills germs.

Do you take raw blood?

No, we don’t take directly but we mix with the soup and take.

Any risk from raw blood?

There is no problem but the educated people are proud and refuse to take it but there is no problem really.

Do you assist in parturition with bare hands??

I don’t know how gloves look like! We don’t use gloves.

Is there any risk for disease from this practice?

(Laughter) We help the animal to give birth then wash hands with the birth fluids and then go to other duties. Then you wash before eating. It is not a problem.

Residing with livestock?

We always keep them with us in the houses. We put the kids because it is very cold outside.

Ever heard of brucellosis?

I have heard and seen people who complain of joint pains who on testing are told they are suffering from brucellosis. They are injected with drugs for a long time and it is a bad disease. I know a child who was recently given this treatment regimen for brucellosis but it is not from milk. Calling it the milk disease is just a name.

Was there any other treatment for the child?

Only hospital treatment.

Any herbal cures used?

None.

Have you ever heard of Anthrax?

We used to hear about it a long time ago but I don’t know the signs.

Have you ever heard of Rabies?

I have heard of a rabid dog which is just killed.

Would you like more information about zoonotic diseases?

I would like to know the cause of brucellosis.

Best way to pass the information?

When there is a group, everyone will hear and be enlightened.

Any question?

Is there any treatment for these diseases and what are the appropriate medication.? For us we have only ever called a doctor once when a cow had a prolapsed placenta.

Why don’t you call a doctor routinely?

We just don’t; we use teramcycin and penicillin. If it is olorobi we use the dip medicine on the wounds. Amboseli park which is nearby also has soda ash and when the animals step on the soda ash they recover from olorobi.

END
