# Supplementary material for: Prioritization of livestock diseases by pastoralists in Oloitoktok Sub County, Kajiado County, Kenya
Source: PLoS One. 2023 Jul 12;18(7):e0287456. doi: 10.1371/journal.pone.0287456 (PMC10337939; doi:10.1371/journal.pone.0287456)
Supplement: S1 Data — (ZIP) [file pone.0287456.s001.zip › Oloitoktok transciptions/KII 4.docx]

**KII**

I: Please tell me a little more about your role here in this Sub County?

P: I am involved in the management of public health issues. This includes preventive medicine, school health, sanitation, vector control as well as water and food quality. Also, some outbreak control and disease surveillance.

In terms of disease surveillance what do you do?

I monitor the transmission of data because I have a disease surveillance coordinator who is under me. I supervise the activities and respond to disease outbreaks. Whenever they are responding to those diseases, I look for ways to support them in terms of technical support.

Which diseases have you been looking at in the last one year?

We have looked at a number; the latest was outbreaks of fever of unknown origin we thought it was COVID-19 but it was not. We have also handled outbreak of dysentery in a school about two weeks ago and previously we have had diseases like cholera although that has been in the past. We have also handled cases of brucellosis.

What do you do in addressing brucellosis?

We have not done much…. it is mainly health education. Ah…because we live with our animals and transmission is through animals to man therefore, we have had to just inform our people. We have also had cases of Rift Valley Fever and we took the initiative to educate our people through barazas. Others are rabies which we have investigated. So, the most we can do is giving education but we also have vaccines for rabies. For brucellosis it is mainly education. Mainly health education to inform them how to handle the aborted fetuses and milk …. all those channels of transmission.

Please tell me a little bit more on what you do regarding training the community?

We have chief barazas and any other forums that we can take advantage of and also at our health facilities we also have our continuous medical education (CMEs). We give health education to our clients…. in each facility they give health education. And depending on the magnitude sometimes we use churches and the pastors give the information to their congregants. We also use posters too which the community health volunteers (CHVs) give to the churches and schools. We have many avenues for health education.

Are there any other common zoonotic diseases other than rabies and brucellosis?

Rabies is common and we also have brucellosis which is common. We had a study and it found these two diseases to be prevalent in this locality. We also have RVF but it is seasonal. We also have snake bites as well as other diseases ….

What about Anthrax?

It has gone down but it used to be there. I don’t know if it is the kind of animal husbandry that is practiced here. We don’t see many cases.

Please tell me about these diseases prevalence in terms of either the rainy or dry season?

Brucellosis and rabies are there all the time. RVF is seasonal. The challenge that we have with RVF is that now that it comes once in a while we can misdiagnose and so unless the person is affluent enough and they are transferred to a bigger hospital like Mater hospital in Nairobi that is when we realize the patient had RVF. So, there can be misdiagnosis unless the patient goes for specialized diagnosis. Then we realize someone had the condition and we didn’t know. We see them as any other condition unless a better diagnosis is done. For brucellosis our labs can do diagnosis but we don’t track it so much, (how the prevalence is) because the cases are few unless it is an epidemic. The hospitals can test for brucellosis. There could be other diseases. You know one of the problems we are having is that some of these diseases are not very clear in our DHIS reporting tools so we lap them together as fevers because the diagnosis was not done well or they are not there in the reporting system. Because if you look at our reporting system there are diseases which could be there but are not specifically put in the system so we don’t know the prevalence and incidence so some of these diseases we could be missing out the opportunity to diagnose them but the most prevalent are like brucellosis and rabies.

What has been the community response to this education and sensitization?

The community is very receptive especially for human diseases. And I also forgot to talk about worms because some worms can be transmitted from animals; but these diseases are there. The community having had animals for a long time makes them understand what we are teaching them very fast and they relate to the experiences they have had and seen. The community is very receptive and when we explain they get it and they take the information.

Please describe their health seeking behavior related to zoonotic diseases?

Generally, women come to the facility, they seek health care faster. Men take time because they assume they know what it the disease is that they are suffering from. Thankfully, we have many facilities in the sub county and that accessibility makes them come. So that anybody is willing to go to the facility and get checked but the lab facilities are few so diagnosis is poor. People have to travel to the district hospital to be diagnosed so that could still be a challenge in terms of people getting the correct diagnosis.

Do they use herbal treatment and over the counter medicine?

People are changing but most people would go for herbal medicine first. But people are changing to conventional medicine and the rate at which herbal medicine is being sold in our markets is going down showing that people are changing their habits now. But generally, we still have herbal medicine and even for COVID -19 people are taking herbal medicine.

Kindly explain to me the kinds of collaboration you have with the animal health professionals?

We work as partners and we join when we share data but rarely do, we have time to share data because each department is doing its work until there is an outbreak of some kind and then someone will say “let us inform this other department of what is happening”. But we rarely share data because we don’t have those forums for sharing data at the sub county level. We don’t have those forums, if we had those forums, we would share how many cases of disease we all have seen. Sometimes even vaccination of animals is being done and the Ministry of Health (MOH) doesn’t know that it is happening. It is often done supported by an organization and nothing is said to MOH so that collaboration is low because we are lacking those forums. We meet once in a while when there is need. Such as when there is an outbreak then we share information and meet. When there are no outbreaks, each Ministry focuses on its own work.

Do you think such a forum would be useful for for vets, medics and environment officers?

Yes, it is long overdue and needed so that we are able to share data quarterly so that everybody knows what is being done or planned and the shortfalls and challenges. It would be very important now that we live with animals. We also have chicken rearing which can predispose people to different conditions so when we are able to share data especially quarterly to know what data we have on different diseases then we can organize our control activities better. So, the forum would help for data sharing, planning and coming up with interventions because if you work on only one side and the other side is not worked on you cannot achieve much. We therefore need these forums. We will know which areas are most important and what messages to pass to the community.

Please tell me if zoonotic diseases are prioritizated here?

Mmmmh….out of all that we have spoken you can say the priority is not there right now because if we did, we would be having forums and data sharing too for the same diseases and interventions put. But as at now we handle them like any other conditions and maybe less.

Why is this the case?

The problem is there is no forum for sharing and thus with no forum you won’t see the magnitude and also diagnosis is a challenge. We are not able to specifically diagnose to tell what is on the ground. This means with no data there is a lack of interest.

What about in the medical sector…are these diseases prioritized?

We handle them like…. of course, there are other more…(pause) challenging conditions other than zoonotic diseases. So, their priority is like sanitation related diseases, so if something is done to reenergize the staff on the zoonoses maybe they would be given more attention. Something will be done because as at now we have so many diseases to focus on so the ones on the DHIS like respiratory diseases, diarrheal diseases, diseases of the eye so your attention will go straight to these but like brucellosis is a mountain but a smaller one thus not prioritized. It is a lower mountain…(laughter) but so long as the data is there people will focus on them too.

How would you prioritize these zoonotic diseases (rabies, RVF, brucellosis and anthrax) in terms of frequency and severity?

I would start with brucellosis which is more prevalent and there was a survey that was done and it was high. Then I would go to rabies then anthrax then RVF as it goes down. RVF is seasonal occurring once in a while and anthrax happens once in a while too so you cannot talk about these and they are not there but brucellosis we know it is there. RVF, you talk about it when there is an outbreak. RVF affected intilal area, the vet guy can tell us why. I don’t know. Anthrax is not in a specific place and cases are very few. Brucellosis and rabies are cutting across but sample taking with rabies is a challenge because proper diagnosis is needed by taking the samples to Nairobi. To confirm it is rabies samples have to be taken to Nairobi and that has cost implications so we just treat for dog bite but we don’t confirm that the patient has rabies.

Thank you for your time and the insights. Do you have any questions?

You are welcome. I don’t have any questions.

END
