# Supplementary material for: Prioritization of livestock diseases by pastoralists in Oloitoktok Sub County, Kajiado County, Kenya
Source: PLoS One. 2023 Jul 12;18(7):e0287456. doi: 10.1371/journal.pone.0287456 (PMC10337939; doi:10.1371/journal.pone.0287456)
Supplement: S1 Data — (ZIP) [file pone.0287456.s001.zip › Oloitoktok transciptions/IDI F 1.docx]

**IDI**

I: How long have you kept livestock?

P: I have grown up in a household keeping livestock.

Which livestock do you keep?

Cattle, goats, sheep and donkeys

Why do you keep these animals?

We keep animals and take care of them because we sell them to buy food and to pay school fees for our children. We also gain milk and meat from them as well as fat which we extract from the milk and use for cooking.

Grazing areas?

Around the oloile village

What about during the drought season?

We go where there is rain like Risa and Olgulului which are in Kajiado south (lengisin entonet ward).

Do livestock here interact with wild animals?

Yes, they do. We do farming here so when the wild animals come here we chase them away but when the livestock go to Olgulului they interact with wild animals.

Which wild animals?

Giraffes, zebras, antelopes, wildebeests, impalas and many others.

Do you ever take livestock to Tanzania?

Yes, we do. When there is drought, we go to Tanzania.

When is there drought?

Extreme drought is in oct-nov and that is when we go to Tanzania

Challenges you face here?

There are many problems like FMD and it is a challenge because we lack vaccines. And also drought because many animals die. And we depend on livestock. The problem is mainly lack of treatment for the animals and drought.

Common livestock diseases?

Olorobi, Olekipei, MCF and also ticks which cause diseases ;these are for cows. For shoats there are FMD which affects their legs, enariri (SNG), CNS disease (engeya ologuny’) and enterotoximia.

FMD symptoms?

The animal doesn’t graze and some are bloated because the animal cannot move. Also, treatment helps so we use teramycin for olorobi.

Can FMD be transmitted to people?

Yes, because the milk comes from the cows and at times you can have olorobi and the cow has not yet exhibited the symptoms and then you know the cow has FMD and so you don’t milk it again.

Signs of FMD in people?

It gets into the whole body, headache, joint pains and comes from taking the milk.

Are there seasons when olorobi is most common?

When it is cold that is when we see olorobi mainly during the months of July up to mid Aug.

Please tell me the treatment you seek for olorobi?

We go to the hospital.

Herbal medicine for olorobi?

It depends, I don’t take herbs but some here take herbs.

Why don’t you take herbs?

The herbs are very bitter so I cannot take but the ones that are not bitter I can take.

Ever suffered from olorobi?

Yes, it causes tiredness, body fells not normal so I go to the hospital. I don’t buy over the counter medicine I just go to the hospital because I cannot take drugs and I don’t know what disease I am suffering from. But sometimes I buy brufen for headache especially if the hospital is far.

Is Olekipei transmitted to people?

I don’t know

Signs of Olekipei in goats and sheep?

It damages the lungs. Coughing and the animal doesn’t graze.

Is Enterotoximia transmitted to people?

No

Is CNS transmitted to people?

No

Is MCF transmitted to people?

No

Signs of Enterotoximia in animals?

This disease affects the fat animals because the animal is so big so the fat goes to the heart and blocks it and the animal dies. It affects the lungs and heart. It affects shoats only.

CNS affects which animals?

Sheep and goats.

Signs

It sneaks to the bushes alone and has diarrhea

MCF Signs?

Salivating and it won’t be grazing and it is not grazing because the animal becomes partially blind. It affects only cattle.

How do you identify a sick animal?

I look at the eyes and the hair coat and also check the stomach for signs of hunger.

How do you treat a sick animal?

Teramycin and then wash in the dip.

Why teramycin?

Most of the time the animal recovers when we use teramycine but if we inject and the animal stays the same then we look for other means or look for a doctor. We also use de-wormers and a drench taken through the mouth.

When do you call a doctor?

When I see an animal is sick and I inject teramycine but after a day or two if animal is still sick then I call the doctor.

Do you ever use traditional remedies for sick livestock?

We don’t but whenever there is a cow with retained placenta then we give herbal drugs called “ong’aboni” and we give it like water so it urinates and gets out the afterbirth.

Eriri signs?

Pox on the skin of the animal. It looks like patches. The animal doesn’t go grazing because it is sick. It affects cattle and shoats.

Is this disease transmitted to humans?

We had a human pox a long time ago but not anymore.

Is there a possibility of humans getting sick from taking raw milk?

I don’t know because we no longer take un boiled milk.

Why do you boil?

If an animal is sick we will not get the disease ourselves because the milk is safe.

Do all boil milk even young men?

All of us boil milk

Raw blood consumption?

We don’t take raw blood just herbal drugs.

Assisting in parturition? Do you wear gloves?

I do it without because it can be an emergency in the bush and I have no access to gloves.

Risk for disease from this practice?

I don’t think so because all we do is wash hands.

Residing with livestock?

Yes, it can cause disease because the animal smells so animals and humans do need to live separately. Can cause sneezing from the smell but no other disease. Yes, we keep animals like kids for shoats and calves too.

Wildlife and livestock interaction? Any risks?

I don’t know because it is men who look after livestock but maybe they do because even wild animals harbor diseases but I don’t know which ones.

Zoonotic diseases?

Enariri is a pox but not from animals only FMD is zoonotic.

Brucellosis?

I have heard about “engeya gule” and it affects the joints but I don’t know it well or how it is transmitted. Please tell me more about the disease and how I can prevent it.

Ever heard anyone with B in your neighborhood?

I have just heard.

Engeya gule meaning?

It means milk disease.

Anthrax? (Emburuo)?

I have heard but don’t know it well.

Rabies?

I have heard about it.

Tell me more?

When you see a rabid dog and it bites someone you need to take the person to hospital because the person can get the rabies

What would you like to know about zoonotic diseases?

First, I would like to know what causes brucellosis, is it milk? Or meat? And I would like to know how people suffer from it. I would also like to know about olmillo and enariri…we would like to know how to prevent and which drugs to use.

Best way to pass this information?

I would like to know if you are coming for a month or a day so that I can mobilize my group so groups would be most effective.

Why groups?

Because we all are pastoralists and we have a pastoralists group, a business group and a cattle group so if you train us, we can call the group and all of us will be helped.

Groups are women only or mixed gender?

The pastoralists groups are combined but they also have women only.

Do you have any questions?

Tell me more about brucellosis?

I explain at length on brucellosis.

So how will you help us?

I explain about community engagement and stopping risky behaviors by the community.

**END**
