# Supplementary material for: Prioritization of livestock diseases by pastoralists in Oloitoktok Sub County, Kajiado County, Kenya
Source: PLoS One. 2023 Jul 12;18(7):e0287456. doi: 10.1371/journal.pone.0287456 (PMC10337939; doi:10.1371/journal.pone.0287456)
Supplement: S1 Data — (ZIP) [file pone.0287456.s001.zip › Oloitoktok transciptions/FGD W 1.docx]

**FGD**

I: What are the common livestock diseases in this area?

P:Nunuk

Olekipei

Olmillo

Eng’ororo

Engoroti

Engeya enaerogua

Eriri

Olorobi

Enadomonyer

Olodua

Kububo

Engeya ologonya

Oltigana

Osingiri

Olmogo

Orung’onyek

Kileny

Nunuk?

Affects cattle only.

Signs?

(Pause). It cannot walk, isuuro cannot graze and we use ash to put on the back of the animal. And also salivating. It stays three days without water then now you inject with teramycine and it gets well.

To people?

No

When seen mostly?

During rainy season.

Olekipei?

This one is for shoats.

Signs?

There are two kinds. Salivating, there is another one that has heavy breathing and it dies fast. It becomes thin and coughs like a human being and then when you slaughter the lungs are joined together. The lungs are joined and rotten.

Seasons?

All the time

To people?

No it doesn’t

Olmillo?

Shoats

Signs?

Circling, emaciation, makes noise, doesn’t die quickly, doesn’t feed. Very dangerous because it has no cure.

Seasons?

All the time

To people?

No

Eng’ororo?

Only cattle. When a cow has FMD and it takes water it gets this disease so you don’t let it take water. It starts as FMD and if it drinks water, it just dies.

Seasons?

Mainly during the rainy season

To people?

No

Eriri?

Shoats and cattle. And can go to people. When there is eriri we don’t eat the meat or take milk we don’t even let dogs eat the meat because they get sick. We bury the carcass.

Signs in livestock?

Rashes on the coat and very ill especially before the rashes and it kills the animal

Seasons?

All the time

To people?

Yes

How is it transmitted to people?

Through milk and meat. Even dogs don’t eat the meat we burn the carcass or bury it.

Olorobi?

Shoats, cattle and people

Signs in livestock?

Mucous, salivating, unable to walk, legs hurt

To people and what is the name?

Olorobi and we also call it homa in Kiswahili

Signs in people?

Sneezing, shivering, coughing, fever, stretching, headache, no appetite

How from livestock to people?

Through milk and meat. Even when there is no milk, we still get olorobi through the contaminated air.

Seasons?

Mainly when it is windy and there is drought…pause….during the rainy season. In animals when it is raining in people all the time.

Olorobi from animals goes to humans?

No because we get it all the time. Some say that it is from animals to people not the other way around.

Enadomonyer?

Only in goats and sheep

Signs?

Bloody and mucous diarrhea. And then it kills fast. It is very lethal.

Any other signs?

None

Seasons?

All the time but when it is most dangerous is when there is drought when the animals consume the grass that is laden with dust.

To people?

No

Olodua?

Shoats and cattle

Seasons?

Drought season…pause…when it is raining…pause and discussion…rainy season

To people?

No

Kububo?

Mainly cattle. It affects the joints and the animal is unable to walk like it goes to graze and unable to walk back home sometimes you cannot see signs in the morning until animal the is unable to walk back home.

Season?

All the time mainly when animals are walking to another place to look for pasture

To people?

No

Engeya ologonya?

Shoats. NB: Cancel that because it is the same as olmillo.

Oltigana?

Cattle only. We see it when it is raining.

Signs?

Drought season when they go to look for pasture elsewhere because the animals don’t get satisfied so they get sick fast. The meat turns yellow and the meat has a bad odor and then you slaughter it. It also has “Isuuro” also before you slaughter it (It becomes weak).

Why during drought?

We don’t know the cause and don’t associate it with anything during drought

Osingiri?

This one affects shoats and cattle and affects the liver.

Signs?

A cow becomes emaciated and the tail loses the hair and when slaughtered the liver looks diseased…”*imechanika”.*

To people?

No

Olgomonyek?

The shoats become blind. To treat you burn it. You cut near the head put some oil then burn again on the place you have applied the oil so that it can see.

To people?

No

Seasons?

Not common

Olmogo?

Pause. It gets a wound that oozes blood and then the wound continues to be large.

To people?

No

Kileny?

This one the animal does not recover. The hind limbs become weak. Affects shoats only. “*Neuromicroporosis”*

Eng’ororo” (Tryps)

Only in cattle

Signs?

They don’t have hair on the tail, weakening, changes the hair coat eg if it is black it gets a different color

Seasons?

All the time

So the only zoonotic diseases are olorobi and eriri only? Will eating an animal with eriri affect you?

Yes

Which one worst between the two?

Olorobi…others say eriri. Eriri is like olekipei because you cannot eat the meat and olekipei we don’t get treatment and we think people are getting it too because a lot of people have TB. People also become emaciated and cough just like the shoats.

So, among olekipei, eriri and olorobi which one is the worst?

(Discussion)….Eriri because it is very contagious among animals and also people don’t eat the meat because they think if they eat they die. Olekipei follows because it is highly contagious in the area. Many in the area get sick.

Olekipei in people?

It is TB. (Some laugh….)

TB is a challenge here?

Not as much now. We associate olekipei with TB but TB is no longer there but olekipei is still there and it is affecting us very much.

Olorobi?

Olorobi is very common.

So Eriri then olekipei then olorobi?

Yes

How to treat olorobi?

Sona moja and kaluma ya kumeza. When you don’t have those drugs you warm water with ginger, garlic and lemon and take and that works faster than the conventional drugs. We also take herbs like oremit, olgonyek we use these.

Hospital too?

When we are very sick, we go to the hospital. When one cannot work because of lethargy, you go to the hospital because the olorobi is severe and also due to the loss of appetite.

Are children given herbs too?

Some are given to kids but some aren’t

Men and women take herbs?

Yes they take similar herbs

Eriri signs in people?

You get the same pox and affects kids the most with fever, chapped lips, red eyes, mucous and then the pox comes out onto the skin and affects kids the most

Treatment for kids?

You use chicken soup and olng’osua which is a herb. You boil the olng’ousa and steam the child using the herb after it has boiled and mixed with goat or sheep dung. You mix and cover boil then pick a blanket and steam the child in that fluid then after the steam the child cries and cries and then you remove the child and the pox comes out onto the skin and the child recovers. Then you apply oil from goat meat and put in the tea of the child and the child recovers. If a child is injected in hospital the pox doesn’t come out so the child is sick for a long time and the pox gets into the chest and the child gets sick often. It remains in the body and so the child is always sick.

At what point do you go to the hospital?

You remove the pox at home then you take the child to the hospital. It is always inside so the herbs remove it but with injections in the hospital it doesn’t come out.

Olekipei signs in humans?

We have never seen or experienced it. In hospital someone is told they have TB but we don’t know anyone here. This one is a bad disease.

Do people here take raw milk?

We don’t take raw milk anymore that time passed. Here we don’t take raw milk but the boys take raw milk when they move cattle to other areas because they don’t have time to boil the milk.

Why boil milk?

Because of all these diseases which have come

Any diseases from milk?

Sometimes you treat the animal with injections so we are trying to eliminate the drugs from the milk

Diseases?

None. We don’t know because we love milk. We want to know because in hospital they tell us you have “the disease of milk”…brucellosis

What do you know about brucellosis? Why is it called “milk disease?”

When you have HIV does it have meaning like ukimwi does it have a meaning? It is the same with brucellosis which they call the “disease of milk”. It is just a name so there are people who take strong tea and they get brucellosis

Raw blood?

We still consume raw blood even meat mixed with raw blood.

Any disease from that?

We have stopped because of Christianity but there is no disease from it.

Assisting in parturition? Gloves?

No we use our bare hands

Any disease?

We even suck the birth fluids from the kid or calf and there is no problem. You can see we are all ok (laughter) and we all do that

Residing with livestock?

Yes, we do, with the kids “mbalelo”.

Why kids?

So that we can milk in the morning and also due to the cold to protect them

Any health issues?

Only allergies so you sneeze but no disease

Skin or hide used?

We use it after drying it and then we put ashes and use bones to clean it and then we oil it and then we use it as a mattress. These days we sell the hide before preparation. It Is not always used at home.

Do some use them?

Yes some are still using them as mats to sit outside.

Women are the ones that prepare it?

Yes, men bring it to us after slaughter

Any risk for any disease?

None

Do wild animals and livestock interact here?

Yes; Zebras, elephants, giraffes, lions and antelopes graze in the same areas as our livestock.

Diseases from wild animals?

There is MCF from wildebeests when they drink water from the same area as the livestock. It comes from the contaminated water.

Any other transmission routes?

None other

Is it a common disease?

Yes, but not common. Mainly during drought when livestock are taken to wild animal infested areas like Amboseli in search of water.

Livestock diseases prioritization?

Eriri, Olmillo and Olekipei and Olorobi

Which one most priority?

Olmillo because it has no cure. Then eriri because we cannot eat the meat and it has no cure and highly contagious. Then olekipei because it is highly contagious and can kill all the animals in the home. Followed by olorobi because ….but this one is better because once you inject the animal it recovers. Nunuk is treatable too. Oltigana can be treated too.

What do you do when an animal is sick?

We treat it ourselves

Who treats men or women?

Both and we use teramycine and penicillin

Why those medicines?

We go to the agro vets and they advise us. This is the only medication that works.

Do you ever call animal health professionals to come treat the animals?

When an animal is so severely ill we call the doctor eg when an animal has olmogo and if the disease recurs.

Are there any home remedies for sick animals?

Only ash in the case of an animal sick from nunuk in which case we pour warm ash on the back of the animal and let it stay in the sun all day with no water to drink. For olmogo we use fat from an animal and use a hot iron to apply the fat on the animal and it recovers.

Would you like further information on zoonotic diseases? What specific information?

For all the diseases we would like information including non-zoonotic diseases

Best way to reach out to you?

We would like you to come physically and teach us because some of us cannot read and we would like it done in a group.

Any more questions?

(Pause). For Eriri how to prevent?

Elena answered.

END
