# Supplementary material for: Prioritization of livestock diseases by pastoralists in Oloitoktok Sub County, Kajiado County, Kenya
Source: PLoS One. 2023 Jul 12;18(7):e0287456. doi: 10.1371/journal.pone.0287456 (PMC10337939; doi:10.1371/journal.pone.0287456)
Supplement: S1 Data — (ZIP) [file pone.0287456.s001.zip › Oloitoktok transciptions/KII 5.docx]

**KII**

Please tell me a little more about your role in this Sub County?

I have been here for less than a year. I basically oversee disease control activities, meat inspection and offer advice to our Livestock extension colleagues and organizations on how to cater to animal health.

Please tell me about extension services?

We offer to the community education and advice on proper animal husbandry, disease control and proper use of drugs. It can be formal or informal as sometimes the farmer will call me for advice. Even tick control. Sometimes we also get them in groups and go teach them.

What about disease surveillance?

It is often either active or passive. We mainly do passive whereby the farmers call us to report a disease outbreak so we go there and take samples. So, we do this on a need basis and enter this information in our register. We also go to the market and check for active cases and use chiefs as key informants so if there is something going on they call and let us know.

Do you have any scheduled trainings?

At the moment we don’t have because we don’t have facilitation like fuel and vehicles. We do it when facilitated by NGOs because they have the resources so we collaborate. From the sub county level, we don’t do any scheduled trainings therefore.

What are the other challenges you face in conducting your duties?

This Sub County is vast and the road network is poor which makes it hard for farmers to get professional services and we are also understaffed. The terrain, vastness of the area, also drought because it is a vicious cycle. Also, diseases because there is no planned disease control strategy. Veterinary officers are few because even for private practitioners it is not a lucrative area.

On drought, please tell me more?

It happens always. Areas close to the mountain not too affected by draught compared to areas away from the mountain (Mt Kilimanjaro).

What are the common livestock diseases here?

Lumpy Skin disease, CCPP and malignant catarrhal fever ….in the areas around Amboseli and Chyulu hills and FMD which is an epidemic twice a year. Two years ago, we had RVF close to Amboseli and Trypanosomiasis along the chyulu belt. Those are the major ones and PPR and enterotoxemia in shoats and majority of these are notifiable. Anthrax also and blackwater but rarer.

What would you say are the top 3 priority?

CCPP, Lumpy skin and FMD.

How do pastoralists identify sick animals and how do they treat these animals?

Most of these pastoralists have lived with these animals for a long time so they know how to identify a sick animal and even tell you the disease the animal is suffering from so you just go and confirm. They identify disease by checking the behavioral changes in the animal because when an animal is sick it does not feed, water, lags behind and has a rough hair coat and in some like FMD the animal limps. Most have antibiotics in the houses so they treat and call us when they have already tried and it has failed. Most of them have oxytetracycline in their homes and that is more than a requirement. They buy several antibiotics at the market. They get advice from those selling these drugs and they also believe that all diseases are cured by oxytetracycline and by sheer luck many animals recover. It is not true but the animals are hardy and resistant to diseases but they always conclude that the recovery is from the medications they administered.

What are the common zoonotic diseases?

I told you two years ago we had an outbreak of RVF along the Amboseli belt. Also, brucellosis and rabies is a big challenge. And also, anthrax once in a while. RVF mostly occurs due to excessive rainfall. Brucellosis has been there but according to surveillance we did last year with UON we did not find many active cases so we could say it has dropped. We collected samples in the whole cunty and results showed no active cases but we cannot rule it out as the surveillance was not everywhere and it is timebound and we still get cases of animals aborting so it could be there.

What about rabies?

It does occur and the frequency varies. Last month I got information on a rabid donkey but we did not confirm. To confirm you have to take a sample and confirm. But clinically it could have been rabid.

How would you prioritize these zoonotic diseases in terms of severity and frequency?

Rabies is the most severe and frequent. Once signs appear there is no survival. Post exposure vaccines have helped though. Brucellosis is tough because of their pastoral nature but they no longer take raw milk but they still assist in parturition. Then anthrax and then RVF. The former occurs more frequent than RVF but once it occurs it could be Number 2 as a priority but it is not a commonly occurring disease.

Please tell me about the knowledge by pastoralists on zoonoses?

They know but they do not have adequate information. They have knowledge for anthrax they know even rabies but there is misunderstanding. They have a very good understanding of zoonotic diseases.

Do you think they know enough about brucellosis?

They do but not adequately especially on the modes of transmission. You hear them say that for brucellosis they are treated with a long antibiotic regimen and they insist that they did not get the disease from animals so they don’t have sufficient information.

What about for RVF?

It is not common so they do not know but they just complain of massive abortions. Unless it is severe, they don’t see any signs.

Kindly explain to me the county efforts for controlling zoonotic diseases?

In collaboration with other partners, we do vaccinations. We did for RVF last year and also the year it was reported. Rabies we have a vaccination program with other partners. Anthrax also has a vaccine but farmers also do it with private practitioners. They buy from vaccines and ask private practitioners to vaccinate their dogs. In the case of brucellosis, it is mainly educating the farmers because once an animal is affected by brucellosis it should be destroyed and we have not had active cases but it can be controlled through breeding. When you find a male…. the male spread to other animals so if you get rid of the male it actually clears the disease. It requires a lot of antibiotics to treat. The problem with brucellosis is that it is intracellular so clearing it requires a lot of antibiotics and so bearing in mind the issue of antimicrobial resistance (AMR) and drug residues in the animal products it becomes a challenge.

Please tell me the kind of collaboration if any that you have with public health officials here in the sub county?

We do have but not officially through the one health approach. Back in Kajiado we do have a one health team but due to COVID-19 we have not met. I was in Kajiado and we had a one health team. It was working well there because when an animal disease is reported we informed public health officers then they would be on the lookout for the disease in humans. We have a team here but it has not been constituted due to COVID-19.

Are zoonotic diseases prioritized here for control?

I cannot say that they are prioritized. All diseases are prioritized depending on their occurrence. The diseases of importance here like FMD which we do vaccinations though not conclusive due to inadequate resources. When a disease is reported we act whether zoonotic or not.

Do you think the One health committee here would be worthwhile?

Yes, it helps the medics to know when these diseases are around to avoid misdiagnosis on their side (humans). It can help them investigate e.g., anthrax skin rashes. This means that they can do better decision making.

Which professionals are in the Kajiado One health team?

Public health, clinical medicine, environment and also people from the veterinary department. And also, from water as part of the environment. It was led by the county director of medical services and veterinary director as a joint team and that is the same way it should be done here at the Sub County.

Was it working well in Kajiado?

There were not many challenges because there is no logistics involved. We had a simulation exercise one time with the East African Community that brought all of us together on how to manage zoonotic diseases and the channel of reporting and it was very helpful.

Thank you for your time. Do you have any questions?

You are welcome. I don’t have any questions.

END
