# Supplementary material for: Prioritization of livestock diseases by pastoralists in Oloitoktok Sub County, Kajiado County, Kenya
Source: PLoS One. 2023 Jul 12;18(7):e0287456. doi: 10.1371/journal.pone.0287456 (PMC10337939; doi:10.1371/journal.pone.0287456)
Supplement: S1 Data — (ZIP) [file pone.0287456.s001.zip › Oloitoktok transciptions/IDI F 13.docx]

**IDI**

I: How long have you kept livestock?

P: I grew up in a household keeping livestock.

Which livestock

Cattle, goats, donkeys, sheep and chicken. They are my farm and my account. When in any problem I can sell and use for my needs like educating children.

Grazing area?

I take to the wild areas. When it rains there is a place set aside where no one grazes and we use this area for the periods of drought. It is us in the village who set a place aside. We will graze there and then when dry we take them to chyulu from July until it starts to rain in Dec. We take to different place depending on what we have agreed but some place is set aside for grazing until it starts raining again.

Do livestock interact with wild animals?

Yes, they meet a lot like the place set aside is deep in the forest where there are a lot of animals so even herders have to be careful.

Which animals?

Lions, hyenas and leopards.

Others?

Yes, a lot of animals like giraffes and zebras. They meet all the time because there are many animals so the herders are very careful.

Which animals are the biggest problem?

Lions and hyenas mostly but biggest trouble is hyenas because they come to the boma at night especially when it raining and they kill a goat. Lions don’t come to the boma a lot they prey on the animals when they are grazing,

What about zebras etc?

Those are ok because we don’t farm here. Elephants are harmful but they are generally harmless. Buffaloes are very dangerous and we avoid them because they harm people. Giraffe are not harmful too.

Do you take livestock to graze in Tanzania?

We don’t take since the time they were apprehended in Tanzania.

What are some of the challenges you face here?

One is our animals getting attacked by wild animals when they are out in the pasture. Some are killed and others are hurt.

Other challenges?

Diseases like another one that affects the head that has no treatment. It hurts the animal until the animal dies. It is called “engeya ologuny” we slaughter the animal and find the head is filled with water. The animal never recovers even after injections. It is in shoats and cattle.

What are some of the signs?

Turning the head and inability to stand.

Any other diseases?

Olekipei in goats and it is dangerous. An animal is unable to stand. If the animal dies and is slaughtered the lungs are joined and it is a very dangerous disease because it kills animals fast. We use many medications by injecting and one might recover so you use the same medication for all of the other goats to prevent it. So, we try different medicines for this disease.

Others?

Those two…also olorobi in cattle. All the cows become sick but this is ok because teramycin works for this disease. Another one is nunuk which is the cow becomes sick and can stand on one spot all day and then we use teramycin. It is our main medicine here and also, we pour ash on the cow and it gets well. Nunuk is three-day disease. The cow stays in the sun and it gets well after a few days.

Please tell me which diseases you notice depending on the seasons?

Yes, when it rains there is olorobi and it happens from area to area. The cattle cannot walk far and it is common when it rains but nunuk is there all the time. CCPP is all the time.

How do you know an animal is sick?

The animal looks weak and not ok, rough hair coat also salivating too.

What treatment do you often use?

The first thing is teramycin and then we use “veriben” (which is the medication for trypanosomiasis) and then we also use penicillin. We also use herbs like “osoit” when other medication don’t work. We just keep trying until the animal recovers.

Do you ever call a vet doctor?

We just use teramycin. We don’t call the doctors because some inject and the animal dies so we don’t like to call them. There are many doctors who are not reliable some of them the kinds of medication they use once they inject into the animal and the animal dies you cannot eat that meat. That is the challenge. Then it is a loss to us because you cannot even eat the meat but with us we just use teramycin and if the animal dies we can still eat the meat. But we have ever called the doctor once to inject the animals when they had “eriri” (LSD).

Why did you call the doctor in that case?

Because many animals were sick at the same time with LSD and some recovered but we still used the medications we are used to. We call them when we see that the neighbor has called the doctor who treats the animals and they recover why will I also not call him? I will call him because I also want my animals to recover.

Any zoonotic diseases you know?

Yes, like olorobi. When the animals are sick even many people are sick because of the milk that we are drinking from those animals.

Signs?

It is homa ya kawaida. Running nose, chest pain and body aches and we say that we are sick because the animals are sick. Because even if we boil the milk we still get iti think we get it because we are the ones that milk the animals and have to be close to them so we say that it is olorobi from cattle.

Any other? Brucellosis?

I don’t know any other.

Have you ever heard of brucellosis?

I have heard about it, does it come from cows?

Please tell me what you know about it?

I know that once you go to hospital and they say that you have this disease then you don’t consume milk anymore.

How do people get it?

I don’t know if it comes from animals, I have just heard about it.

Raw milk risks?

Stomachache although these days we don’t take raw milk because sometimes it hurts the tummy.

Why do you boil?

We have been educated. A long time ago we would give raw milk to the children but these days we boil.

Raw blood?

We don’t take it anymore.

Even young men?

Nobody does.

Residing with livestock?

There is a bad smell from the animals and it can cause “homa”.

Assisting in parturition?

That one there is no problem.

Is there any risk from livestock interacting with wild animals?

Yes, there are challenges like ticks which bite wild animals and when they bite livestock the livestock become sick.

Any diseases you know from wild animals to livestock?

None that I know of.

How can you prevent zoonotic diseases eg olorobi?

Nothing other than going to hospital and taking medication when sick. Unless taking herbal medicines, these ones can help. We get them from the forest and boil and take. I go to the hospital when I don’t feel better.

Do you purchase medicine from the chemist?

Yes, we do especially Panadol, cetrizin and if I don’t feel ok then I go to the hospital. In hospital they test and give me the right medication.

More information you need on these diseases?

How to avoid contracting these diseases especially brucellosis because how can brucellosis be a disease of milk? I want to know how can milk cause such a disease.

Best way to reach you?

The best is a seminar or a workshop. The posters to inform us about the meeting. Most people don’t know about brucellosis…we just hear that it is a disease of milk.

Are children also given herbs?

No, we don’t give herbs to kids, we take them to hospital because the herbs are bitter and the children refuse to consume them.

Any questions?

Yes. Are you going to bring us this education?

I explain about our community sensitization plans

Any medications for our animals like for “olmillo” and “olekipei”? Please tell them to find medication for these ones because once the animal is sick, they die.

END
