# Supplementary material for: Prioritization of livestock diseases by pastoralists in Oloitoktok Sub County, Kajiado County, Kenya
Source: PLoS One. 2023 Jul 12;18(7):e0287456. doi: 10.1371/journal.pone.0287456 (PMC10337939; doi:10.1371/journal.pone.0287456)
Supplement: S1 Data — (ZIP) [file pone.0287456.s001.zip › Oloitoktok transciptions/IDI F 14.docx]

**IDI**

I: How long have you kept livestock?

P: I was born in a household keeping livestock. I keep shoats and cattle and chicken too.

Why do you keep livestock?

It is my bank. They help me educate the children and it is food for us too because I can sell and buy food or we eat meat and take milk.

Where do you graze the animals?

We take them to the area close Amboseli park but during the drought time we take them to Mbirigani and Namelok. As well as Entonet, in the hills.

When is there drought here?

Aug- Oct that is when the drought is very severe.

Who takes them there?

The herder or even I myself can go. If I have no herder then I can go. I need to go only when the drought is very severe like every three years. We also gather the maize stalks and also buy feed for the cattle that remain at home. The herders can be family members or hired workers.

Do you take animals to graze in the park?

There are wild animals there and they are preyed on but sometimes we do take them so we avoid going to the park but even in the areas we go to graze there are wild animals but fewer.

Do you ever take your animals for pasture in Tanzania?

Not much unless there is severe draught here.

What are some of the challenges you face as livestock keepers?

Diseases, drought and wild animals which prey on livestock mainly hyena and lions but hyenas mostly. They eat all species of livestock.

What about diseases?

Olmillo, Olekipei, Engeya nerogua.

Please tell me more about Olmillo?

It affects shoats and is fatal in most of the cases. The sign is “madness” in the animal. It doesn’t eat, cannot see, gets lost and dies in the wild or is attacked by wild animals and killed.

What are the signs of olmilo?

The animal bellows a lot and circling and no feeding. So, we slaughter and eat. This is a more recent disease, we started noticing it like 6 years ago. A lot of animals die like 10 in one homestead and the animal never recovers. We try to treat with teramycin and penicillin but it doesn’t recover.

When do you do when many animals are sick with this disease in the homestead?

We sell to butchers now after we ate the meat and realized it is not harmful to people. Before then we were afraid to sell because we didn’t know if the meat was safe for people.

Tell me about Olekipei?

It can be treated or sometimes the medications don’t work. It makes the lungs sick. The animal coughs and it is in shoats and sometimes they never recover. We use teramycin and penicillin and there is a specific medication for it too. There is a vaccine for it too. Sometimes they recover and sometimes we find the doctor to give the vaccine.

Please tell me about Engeya enarogua (enterotoximia)?

This one we don’t know about it so well. You just find the goat is dead and we say “inagandisha damu”. Engeya enarogua means “hot disease”. It often does not show signs beforehand. It affects shoats.

Is it a recent or old disease?

This has always been there.

Is Olmillo transmitted to people?

I have never heard of that.

What about Olekipei?

No.

And Engeya enarogua?

Not in people.

Please tell me about Eriri?

In cattle it is called onkoro and in shoats is eriri.

Eriri signs?

“Upele” on the body and no other signs

Treatment for eriri?

We use teramycin and other medications.

Can Eriri be transmitted to people?

I hear that it can. Actually when an animal sick with this disease dies we bury the carcass so that people don’t eat the meat. We don’t eat the meat. And it is highly contagious, some animals live and some die.

Why do you not eat the meat from an animal that has died of eriri?

The meat is dangerous and since a long time ago people do not eat the meat.

Kindly tell me about Enkororo?

It is in cattle and causes heavy breathing. And if the cow takes water it dies. It is unable to breath and also salivation. And we don’t give it water and this one recovers with teramycin.

Can it be transmitted to people?

No.

Any other diseases?

None.

What about Olorobi?

Oh, yes that one is there also. It causes foul breath and cracked hooves. And also wounds on the hooves. It affects cattle and shoats.

Can it be transmitted to people?

Yes because of taking milk. We call it homa in people.

What are the signs of the homa in people?

When the mouth is cracked then the animal may be having olorobi and when we take the milk we get sick. In humans it is a running nose and coughing.

What are the diseases you encounter during the drought?

All diseases occur during drought time.

Does that include oloribi?

Olorobi is common in Nov-Jan and April-May during the rainy season.

What is the treatment for olorobi in people?

Herbs like “lokonyel” and “ndanosiru “and if the person doesn’t recover, we take the person to the hospital. For kids we don’t give them herbs because the medicine is too powerful, we take them to the hospital. When we don’t recover after herbs, we go to hospital. Sometimes we buy over the counter drugs like piriton and paracetamol.

How do you determine when to go to the hospital or use herbs?

If it is not severe, I buy Panadol or other over the counter drugs. If severe I go to the hospital.

Do you know any zoonotic diseases?

I have never heard of any.

How do you identify a sick animal?

Rough hair coat and salivating and Isuuro” (dullness and doesn’t eat) and we use teramycin and penicillin.

Do you ever call a vet doctor to treat sick animals?

These days we are calling the doctor because sometimes we inject and the animal does not recover so we call the doctor.

Any other reason to call doctor?

Yes, there is another disease called namagait and we call the doctor.

What are the signs of “namagait”?

Rough hair coat and emaciation. We call the doctor for this because there is no treatment that we know of.

Diseases from wild animals to livestock?

None but animals meet. Oh, but namagait comes from wild animals through water when they drink water from the same source. Here the livestock interact with wild animals.

Any other disease from wild animals to livestock?

None.

Do people take raw milk?

No, we always boil the milk to avoid diseases like eriri so we have to boil. All people boil milk these days.

Do people take raw blood?

We do take but only from a healthy cow.

Who takes raw milk?

All the people in a household.

Is there any risk for disease from raw blood to humans?

None, we have not encountered any.

Do you assist in parturition and do your use gloves?

Yes, we do but without gloves.

Is there any risk for disease from this?

I have never heard and we do it and we are ok.

Do you reside with livestock in the house?

Yes, we do and the only challenge is allergy so we try to avoid it because it causes sneezing.

Any other disease from this?

Only allergy I don’t know any other.

You said that it is only in the case of eriri that you don’t eat meat from the carcass?

Yes, we don’t but in the case of other diseases we eat the meat.

Have you ever heard of a disease called brucellosis?

Yes, I have heard that it is brought by consuming unboiled milk. I don’t know anything else about it. I know people who have gotten it and that is why we boil milk but I have never encountered it. I just hear about it so I don’t know well about it.

Have you ever heard about Anthax?

I don’t know about this.

Have you ever heard about Rabies?

I know about rabies and it is from dogs which interact with wild animals. It is brought when a dog eats the carcasses of animals in the forests.

How do people get it?

When a rabid dog bite someone the person. So, if that happens, we rush the person to the hospital because the person will die or be rabid.

What would you like to know about zoonotic diseases?

Yes, I would like to know more.

What is the best way to educate you about these diseases?

Even now you could teach or call for a meeting and teach or go teaching from house to house.

Do you have any questions?

Yes, please tell me about brucellosis?

I explain about brucellosis at length.

**END**
