# Supplementary material for: Prioritization of livestock diseases by pastoralists in Oloitoktok Sub County, Kajiado County, Kenya
Source: PLoS One. 2023 Jul 12;18(7):e0287456. doi: 10.1371/journal.pone.0287456 (PMC10337939; doi:10.1371/journal.pone.0287456)
Supplement: S1 Data — (ZIP) [file pone.0287456.s001.zip › Oloitoktok transciptions/KII 2.docx]

**KII**

I: What are your responsibilities in this village?

P: I am a pastor and chairman. Mine is Chyulu and then we are livestock keepers and, in this forest, we make sure that we have trees so that we protect the environment. We farm and keep livestock and I also farm. This is my work in this ranch. They elect the committee for the group ranch and also the grazing committee to organize where the animals graze. I have been here for a long time. I was born in Kuku and have been here in this home for 15 years but in this general area for 30 years. This ranch was started by members and these members are the ones that belong here from 1974. So, we are members and only those who are members can live here but for farming someone can lease land and farm.

I: What challenges do pastoralists face in this area?

P: Drought because it can be severe and we lack water and so we have to take animals to chyulu and it is far. It is about 27 km from here and the animals have to come back here for water. There is no water there so animals lose weight. And also, ticks are a problem because they are lot and the cows struggle to walk as they are always sick so we buy water and transport with vehicles and boda boda and donkeys especially for those which are very sick. They come to water every three days because it is far. Diseases are also a challenge like the one from ticks. Goats do better. Sometimes there are fires in chyulu so we had no grass for our livestock. Like last year so we took animals to Tsavo which is not allowed. The fire was from miraa farmers. Tsavo encroachment is a problem too. We graze within when there is no drought. Draught is mainly from July and onwards that is when livestock move to chyulu with the young men. All the livestock graze together but shoats have a lot of diseases especially Olekipei and animals should not stay even a day without water. When they have olekipei they have to drink water.

I: What are the signs of Olekipei?

P: It is like pneumonia. So, when they lack water, they die.

I: Please tell me the interaction livestock have with wild animals?

P:They come even to the compound. The other day a hyena came in here and caught a sheep. Another time a lion ate a cow and the govt does not compensate. It happens a lot. Wild animals are many here and they attack humans and livestock. If a human kills a wild animal like an elephant, it is a big deal but the other way around no one bothers. Compensation takes a long time and the Govt is not too concerned. Animals come from Tsavo like giraffes, antelope and zebras and they come with livestock. They come these sides because we don’t take our livestock to Tsavo.

I: Do you take livestock to graze in Tanzania?

P: Not us on these sides but those in Rombo do. Magufuli confiscated our animals and auctioned them.

I: What about to Amboseli?

P: That is far as well. They come from there and go to Chyulu those from entonet ward they come to chyulu. Many cattle all over Kajiado go to chuylu during the drought season.

I: Kindly tell me about livestock diseases in this locality?

P: There is Ndorobo, Olekipei, Lipis, Eriri

I: Lipis?

P: A cow swells on the neck.

P: Eriri…the animal has sores on the coat. Another one is “ya kichwa” and doctors have been unable to cure it. This one is when worms get into the head and when they start going around, they cause the goat to start going round and wailing and it has no cure. The goat has to be slaughtered and it causes no harm in humans. They told us to bury the head to avoid transmission and it is a bad disease. It has no cure but the other ones have a cure. Another recent one for goats and doctors came to cure it is enterotoxaemia this one they brought medication and animals are ok like my goats recovered. All the other diseases have a cure.

I: How do you treat animals?

P: We treat them ourselves. We look at an animal and we know which disease it is suffering from. We call the doctors when the disease is very severe. Like the Enterotoxaemia one I called the doctor and he came and treated.

I: Please tell me about zoonotic diseases?

P: I know Olekipei because here we say it causes TB in people when there is a lot of cases of Olekipei then humans cough a lot too. Another one is Olorobi. This one also is transmitted to people through the drinking of raw milk. These days we are educating people to boil milk and people understand the importance of doing this. Ndorobo is not transmitted to people but Olorobi and Olekepei is transmitted to people. People cough a lot.

I: Lipis?

P: Affects the neck but not much.

P: Iriri…skin disease

I: What are the signs in humans for olorobi?

P: Malaria…olorobi is malaria. It happens mainly from Jan/Feb. Olekipei is all the time and lipis is during the rainy season. We use teramycin, ndorobo is when they go to chyulu and ticks bite them. Iriri is not common.

I: Treatment seeking behavior?

P: In chyulu there are many herbs which we harvest and boil. You mix three, some you use with tea and we have experts and also older people like us we have the medicine. These prevent disease but some cure even malaria. Some are for stomach ache because we don’t have a hospital. We like these herbs but the educated people have to go to the hospital. As the older people we prefer herbs and they help. We slaughter a goat and take soup with herbs. Younger people don’t take herbs but us older people we like them.

I: Malaria/Homa signs?

P: Malaria we know and are telling them to boil milk. Our problem is that we eat meat from diseased animals and this can cause diseases. Olekipei is a big issue in humans from goats to humans.

I: Anthrax?

P: Anthrax is bad when you eat the meat. It is transmitted to people and it is rare here and we are not eating the meat. In the past it was a bigger problem now we avoid eating that meat.

I: So transmission is from milk and meat?

P: Yes

I: Any other routes of transmission?

P: Vets teach us so that we know what is going on so that we can prevent disease. People are boiling milk unless in the remote locations where some may not. The doctors are educating us though.

I: Priority FMD or CCPP?

P: CCPP because it is there all the time and it is in shoats. We eat meat from CCPP animals the goat coughs and is emaciated just like a TB patient. If someone gets it we say it is TB. Then FMD. In humans it is mainly through sneezing. It is “homa”. Lipis no and iriri not much in humans.

I: Rabies?

P: Doctors vaccinate and it is not a common disease.

I: Brucellosis?

P: Yes, a lot. I wonder though because I have a friend in Mtito who was told that he was suffering from this disease and yet he does not consume milk at all even milk in tea. So, this is just a name they have given this disease it has nothing to do with milk. I don’t know how it is transmitted. It is very common here and people are injected 21 injections and there is also *ugonjwa wa maji.* I don’t think it is from milk because even those who don’t take milk get it. We have investigated and we have concluded it is not from milk.

I: Preventing FMD and CCPP in humans?

P: Yes, FMD the challenge is abortions in cows and I don’t know why. Even women abort when they get Olorobi. We have a vaccine and when we vaccinate then we prevent and for women if they are pregnant, they don’t take the milk or meat from a diseased animal with FMD. Women know because it is common knowledge and so they don’t consume these. Others eat but the woman refuses. Older women advice the younger ones. For CCPP we don’t know because it is there all the time. This one we don’t know how to prevent. It is a disease that is there all the time so we drink the milk and meat we say after all the animals have been vaccinated. That is what the doctors tell us. FMD has a vaccine but only by the vets but CCPP we can vaccinate ourselves. For FMD we use taramycin.

I: Are there any health risks from handling aborted materials?

P: Yes, FMD.

I: Is there any way to reduce that risk?

P: No there isn’t just to inject the animals when they are sick.

I: Where to buy medication?

P: In the chemists nearby. We just buy.

I: Any question?

P: Are you students?

I: I explain about HORN and us being students at UON.

P: What actions will you take after this research?

I: I explain about the goals of this research and the follow up community engagement.

P: Are you gonna bring medication especially for the diseases that are affecting our livestock the most?

I: I explain our role and the role of others including the Sub County officer for livestock.

**END**
