# Supplementary material for: Prioritization of livestock diseases by pastoralists in Oloitoktok Sub County, Kajiado County, Kenya
Source: PLoS One. 2023 Jul 12;18(7):e0287456. doi: 10.1371/journal.pone.0287456 (PMC10337939; doi:10.1371/journal.pone.0287456)
Supplement: S1 Data — (ZIP) [file pone.0287456.s001.zip › Oloitoktok transciptions/IDI F 7.docx]

**IDI**

I: When did you start keeping livestock?

P: From childhood.

What livestock do you keep?

Sheep, goats and cattle and donkeys.

Why do you keep livestock?

I keep livestock so that I can sell and have money to educate my children and I also keep them for food.

Please tell me where you graze livestock during the rainy and drought seasons?

We graze them around here but when it rains we take them to the mountains that is chyulu hills. They go to Chyulu from Aug until when the rain starts in Dec.

Do livestock interact with wild animals?

They interact. There are lions, elephants and hyenas also.

Do you ever take livestock for pasture in Tanzania?

We don’t.

What are some of the challenges you face as livestock keepers?

There are diseases which kill all the livestock like CCPP, the CNS disease (olmillo for sheep and goats). For cows it is olorobi and lipis. Lipis affects the animal for two days and then it dies. We know an animal is sick when it has “Isuuro” which means the animal is dull and doesn’t graze and the haircoat is not normal. There is also nunuk (the three day disease called bovine ephemeral fever). These are the diseases and also wild animals which prey on our animals.

Any other challenges other than diseases?

FMD also affects people. The wild animals kill our livestock.

What are the signs of Olorobi?

The animal is unable to walk, body is weak and struggles to stand up especially in the morning when it is cold.

What about Olekipei?

The animal coughs.

What about the CNS disease?

Circling and animal don’t respond to treatment.

And Nunuk?

For nunuk it means the animal “inajikuja”…that is what we call nunuk (which is 3 day disease or bovine ephemeral fever).

Any other diseases?

None.

How is olorobi transmitted to people?

It affects your legs and you are unable to move and you have a headache and backache and general body malaise.

Kindly tell me about the seasonality of these diseases?

FMD is during the rainy season, nunuk is all the time, CCPP is always there when there is rain.

Are there any diseases that common when animals go to the chyulu?

Engeya enerogua which is in sheep and it is enterotoximea and ECF also.

When is it dry/rainy here?

It rains from Dec to March and it is dry in Aug to Nov.

Prioritization of diseases?

FMD then CCPP and also the CNS disease.

How do you identify a sick animal?

“Isuuro” the cow is dull, doesn’t graze and the rough hair coat. “Isuuro” is a broad term.

What do you do when you notice the “isuuro”?

We use teramycin and tie it down so that it doesn’t go to graze with others. We also use veriben which is indicated for trypanosomiasis.

How do you use veriben?

We mix it with tetracycline and inject into the animals.

How do you know how much medicine to administer?

We just check the ccs and inject depending on the size of the animal.

Are there any traditional curative methods you employ for livestock?

We also use some herbs which we boil and give to the animals. For example, we use “Omomwenye” for an animal that has aborted.

How do you determine if to use traditional or conventional medicine?

If the teramycin hasn’t worked in three or so days we use herbs.

Do you call livestock field officers to treat animals?

Yes, we call the doctors when the animals have FMD. That is often about once in a year.

Why not for the other diseases?

Sometimes FMD is very severe and we are unable to deal with it.

Do you know how zoonotic diseases are transmitted to people?

When the cow is sick and you take the milk then you get olorobi.

Raw or boiled milk?

When the milk is raw.

Any other zoonotic disease that you know?

For enerogua (enterotoximeia) if you eat meat from the animal you get some pox called engoroto.

What are the signs of the pox?

It is a wound that develops in one area. I don’t know any other.

Between FMD and enterotoximeia which is the priority?

FMD.

Have you ever heard of brucellosis?

No.

What about anthrax?

No.

Rabies?

Yes, I have heard about it and we call it “orairugua”.

How is it transmitted to people?

When a rabid animal bites you, you get rabies.

Diseases from wildlife to livestock?

Yes, there are.

Which ones?

“Engea nerogua” (enterotoximia), the cows get it and the animal dies immediately; before treatment.

How is it transmitted from wild animals to livestock?

When they interact in the forest.

Diseases from raw milk to humans?

FMD only.

In humans is it also olorobi?

Yes.

Raw blood consumption?

Nobody takes raw blood.

Is there any risk for disease from assisting animals in parturition?

No, there is no risk for disease.

Do you reside with livestock in the same house?

Yes, we do.

Which animals and why?

It is mainly the kids because we want to milk the shoats in the morning so we don’t want them to suckle at night and there is no risk for any disease from this practice.

Have you ever had an episode of zoonotic diseases in your household?

Yes, I have and we went to the hospital. Sometimes we use herbs to treat the person. We use the root of the Osoit tree and boil and then when it has cooled, we drink it. We start with the herbs and then if not, effective we go to the hospital and vice versa.

What are the symptoms that make you go to hospital?

When very sick like with a headache, backache and weak legs.

Do you give herbs to children also?

Sometimes we give them and sometimes we take them to hospital. When they are feverish and coughing then we take the kids to the hospital.

What are some of the ways of preventing olorobi in humans?

We make sure that we don’t take un boiled milk and also if the cow meat was from olorobi we cook it in a special way. We boil the meat, pour the water and fry again and make it dry.

Why do you do that?

So that the germs that killed the animal die and thus cannot harm us.

What about if an animal died from enterotoximia?

We do the same thing.

Do you always boil milk?

Yes, we don’t take raw milk.

What about the young men who move with livestock to the chyulu hills do they also boil?

No those ones take it raw as they don’t have time to boil the milk.

Would you want more education on zoonoses?

Yes, I would like some more information.

What exactly would you like to know?

How olorobi is associated to olorobi in humans and also about enterotoximia.

Best way to pass this information?

Call a meeting and also use the schools to train us.

Any question?

Why are you here? Do you want to help us?

I explain what the goal of this study is and especially on community education.

End
