# Supplementary material for: Prioritization of livestock diseases by pastoralists in Oloitoktok Sub County, Kajiado County, Kenya
Source: PLoS One. 2023 Jul 12;18(7):e0287456. doi: 10.1371/journal.pone.0287456 (PMC10337939; doi:10.1371/journal.pone.0287456)
Supplement: S1 Data — (ZIP) [file pone.0287456.s001.zip › Oloitoktok transciptions/IDI F 6.docx]

**IDI**

I: How long have you kept livestock?

P: Since I was young, I have grown up in a household that keeps livestock. I keep shoats and cattle and chicken.

Why livestock?

They are beneficial to me for example I get milk and meat and also as a source of income.

Grazing area for your livestock?

During drought I buy maize stalks and feed the animals but in when it is not drought time they go to the wild areas “porini”. Or they move to another place during drought. We move most of the animals but we remain with some animals at home for milking which we feed maize stalks. Some of the areas they go to during the drought time are Risa in Lengisin ward. We move even the goats and sheep.

Who moves the animals?

Young men do it and because the animals are there for a long time, they keep exchanging; some stay home and other go. However, these days it is often a hired hand because many young men are now going to school.

Do you take your livestock to Chyulu hills?

No, we haven’t in the recent past.

What about to Tanzania for pasture?

It has been a long time since we went to Tanzania for pasture. We only go when there is no rain in this whole area, but we start within the areas I mentioned earlier.

Do livestock interact with wild animals when grazing?

Yes, especially hyenas and they attack livestock at night.

Other wild animals?

Not much. But in Risa which is close to Amboseli there are a lot of wild animals including lions and buffaloes.

What are some of the challenges you face in livestock keeping?

There are diseases like Olorobi and Eriri and Olekipei in shoats. Drought is also a big problem. Wild animals too prey on the livestock when they go to the Risa area.

What are the other common livestock diseases beside olorobi and olekipei?

Eriri also but not as common as olorobi and olekipei which are worse. As well as worms which also kill sometimes and nunuk. After rains; when the rains have just stopped there is bovine ephemeral fever (nunuk).

Which animals are affected by Olorobi?

It affects cattle and shoats.

What are the signs?

Wounds on the mouth and hooves and smell from the mouth. Pus from the hooves also and it can be unable to walk home from the grazing area and has no milk letdown.

Seasons when olorobi common?

A long time ago it was during rains but now it is all the time even during the drought season.

Can Olorobi be transmitted to people?

Yes, when one takes milk, you can get olorobi even if you have boiled the milk. When the animal have olorobi even people get it and vice versa.

What are the symptoms of olorobi in people?

Sore throat, when you have sore throat, you say it is from the cattle. And sometimes you get the same signs but not transmitted from cattle.

What are the signs of olekipei?

It is only in shoats and the signs are coughing and diarrhoea.

Is this disease transmittable to people?

No, it is not.

Are there seasons when olekipei is more common?

It is there during all the seasons and it is highly contagious.

What about eriri?

This disease affects cattle and shoats.

What are the signs?

The animal has kind of a pox with nodules on the skin and we don’t eat the meat because it has a foul smell.

Can eriri be transmitted to people?

We don’t take milk from that cow. But it cannot be transmitted to people.

Please tell me about nunuk?

It is only in cattle and cannot be transmitted to people.

What are the signs?

Shivering and the animal is unable to walk and thus the animal is stationary.

Are there seasons when this disease is more common?

When it is hot just after the rains.

Do you know any other zoonoses?

No, I don’t.

Have you ever heard of brucellosis?

Yes, I have ever heard.

What do you know about this disease?

It causes backache and inability to walk and you are told to rest and not take milk but we don’t think it comes from milk because even those who don’t take milk get it. So, we don’t believe it comes from milk.

Is brucellosis a disease of animals too?

No, it is not.

Have you ever heard of anthrax?

I have not heard.

What about rabies?

I have heard of rabies and there are rabid dogs. The rabid dog bites people and we take the person to the hospital for injections.

How do you tell an animal is sick?

When an animal is shivering and there is no milk let down.

What do they do when you notice this in your animal?

We buy drugs and inject the animal; we use penicillin and we also call a doctor.

How do you know when to call a doctor?

We call the doctor after trying for three days to treat the animal unsuccessfully.

Are there any traditional methods that you use?

For an animal that has not expelled the after birth we use a herb called the “Olmumunyi” which we boil and then after it cools we feed the animal and the placenta comes out.

Any other methods that you use?

“Endebe” which we use by giving to the shoats for removing the after birth and sometimes for cattle too.

Do you take raw milk?

We always boil milk but the very young children are given colostrum which is not boiled.

Why do you boil the milk?

To kill the germs.

Are there any diseases that come from raw milk consumption?

No disease I know of but we take boiled milk.

Do you consume raw blood?

We don’t consume it but men take it when they slaughter an animal. We take meat that has been cooked but mixed with raw blood though.

Diseases from raw blood consumption?

No diseases.

Do you wear gloves when you assist animals to give birth?

None, we do it with bare hands.

Is there any disease that can arise from this practice?

None.

Do you keep livestock in your house?

Yes, we do for the kids so that they are not affected by the cold outside when young.

Any risk for disease?

None.

Are there any diseases that can be transmitted from wild animals to livestock?

Yes, there are.

Which ones?

“Enguruya nchaget” (MCF) especially when the animals move to chyulu hills. We hear that it occurs when animals are moved to that area. I have ever seen a cow with MCF when my cows went to Chyulu. The joints were very weak and it was blind and people said it was MCF.

How is transmitted to livestock from wild animals?

It is transmitted when wildlife give birth and then they go to the sources of water and urinate in the water and when livestock drink the water they get MCF.

Any other from wild animals?

No, none other.

Would you like to learn about zoonotic diseases?

Yes, I would

What would you like to know?

I am very interested in brucellosis how is related to milk and how is it transmitted. I have also head of meat disease and would like to know more.

What are the signs of meat disease?

I have only heard about it like milk disease (brucellosis).

What is the best way to pass this information to you?

The best is to call for a group and do a training.

Why a group?

Everybody will get the answers to the questions they have and everybody will get knowledge of the disease.

I explain about brucellosis.

**END**
