# Supplementary material for: Prioritization of livestock diseases by pastoralists in Oloitoktok Sub County, Kajiado County, Kenya
Source: PLoS One. 2023 Jul 12;18(7):e0287456. doi: 10.1371/journal.pone.0287456 (PMC10337939; doi:10.1371/journal.pone.0287456)
Supplement: S1 Data — (ZIP) [file pone.0287456.s001.zip › Oloitoktok transciptions/KII 3.docx]

**KII**

I: About how many villages are in the ward?

P: Around 20.

How long have you worked in this area?

I have worked in this office for 2 years but since I completed school, I have done this work for five years. Before I started working in this office I was in private practice. In the office our work entails issuing transport permits, no objection letters for transporting animals from outside the county to here. Also, if we are under quarantine, we don’t issue these letters. We allow the animals. We issue permits for transportation from here. We do vaccinations and simple treatments. If there is LSD or FMD we don’t allow animal movement. We offer extension services like educating farmers on controlling the diseases.

Please tell me about the community education services that you offer?

There are many NGOs here like “water is life”. With their facilitation we teach farmers how to do livestock as a business not just a lifestyle, we teach them how to fatten the animals and how to vaccinate them too. We also teach them how to treat animals without mixing the drugs and also how to follow the right treatment procedure. This is because they often either under or over dose animals so that is why we need to educate them.

Kindly tell me how you do disease surveillance?

When there is an outbreak, the farmers report to us e.g., a case of FMD or sometimes we notice when we are in the field. We alert the farmers and the officer in charge so that there is vaccination. So, they come here to report or they call us. We work in partnership with other NGOS, the “water is life” organization works with famers in entonet ward. Now they know us as the officers and they involve us. In the past they didn’t. This NGO showed them how the government can help them.

What are the main diseases in this area?

FMD, Lumpy skin disease, heartwater, PPR, enteretoximia…it’s a lot here….(pause). There are abortions too but we fail to understand if they are due to brucellosis or RVF or any other cause. There has been no investigation but there are mass abortions. And also, MCF because of the many wildebeests.

Of these which are the notifiable diseases?

Foot and Mouth Disease, Lumpy Skin Disease, Malignant Catarrhal Fever and rabies.

Which zoonotic diseases you have encountered last six months?

There is brucellosis because it is there in humans and people tell us so I can tell that the animals are sick. So, brucellosis is around but it has not been investigated. Also, rabies we are trying to fight it but the farmers don’t turn up for vaccination of their dogs as they don’t look after dogs. They don’t value the dogs.

Is Rabies in humans common?

Yes, there are cases. The thing is that people are ignorant. Many times, they don’t take those who have been bitten to the hospital and some kids who were bitten have died. For cattle they come for vaccination even sheep and goats but not dogs. They don’t value their dogs that much.

Please tell me about Anthrax?

There are no cases, even for TB. Many of the cases are either rabies or brucellosis.

What control strategies are in place for brucellosis?

None. The pastoralists don’t believe they get brucellosis from contaminated milk so they don’t take precautions. There is nothing we are doing about brucellosis in animals. We are not doing anything as we are not facilitated. When we go to the field, we tell them not to take uncooked milk but we are just trying to tell them but we are not sure that they are practicing this. Contact with wild animals also still happens. There is no money for such things like controlling brucellosis.

What challenges are pastoralists facing here?

Predators for cattle when they go to pasture as well as disease outbreaks like FMD. If we don’t provide free vaccination, they cannot afford to vaccinate all their animals because some have even 500 cattle so they cannot afford to vaccinate all these animals. If the Govt doesn’t provide free vaccines they don’t vaccinate their animals. No other challenges because the markets are there but they don’t sell animals when drought comes, they all die and they start all over again. This is because cattle are a source of identity and a lifestyle. There is also drought which can last a long time so they just keep moving their animals. A lot of animals die.

Is farming practiced here?

Not really but in the upper part of the Sub County yes, but not in the lower parts. There is no water in areas like olulului and Amboseli but in the upper parts they plant because it rains and the temperature is lower.

Kindly tell me about diagnosis and treatment of livestock by the pastralists?

They just use guesswork because they come to the market and buy teramycin, penicillin and dewormers. For dewormers, I can say they are doing better because they deworm but I don’t know about dosage and we have taught them the cycles of deworming which are; a month before the rains, when it rains and 3 months after the rains and this, they do 3 times a year. We have taught them the dosages also by estimating the weight of the animals because they have been killing the animals through over dosing. For treatment they don’t differentiate between teramycin and penicillin they can inject the former in the morning and the latter in the evening. So, there is drug interaction and the animal dies so we have educated them to use either for three days not interchanging. They also inject in the morning and then they leave it and don’t inject again so they underdose like they do the injection once and don’t do it again. Then they wait for it to die and then they slaughter. The aim is to keep the animal so they don’t slaughter until it is too sick and then dies. They consume the meat at home. They slaughter once in a while but nowadays because their kids are going to school, they are selling animals for school fees.

So, you said the main zoonoses here are brucellosis and rabies?

Yes.

Please tell me about diseases more common during either the rainy or dry season?

Most of these diseases are common during the rainy season because most animals calve during this period and there is milk so disease is higher in humans due to milk consumption. Also, for the dogs it is also mating time and they bite each other as they mate and fight for females. This happens in Aug/Sep and so the cases of rabies are higher even in humans.

Do you vaccinate dogs based on this?

(Laughter). One farmer can ask me to do it so I buy the vaccine and go vaccinate but here from the office we wait for facilitators from elsewhere to come so we have the funds to organize a community vaccination exercise and that is when we vaccinate because they come with the vaccines themselves. The farmers are not willing to pay and when the facilitators come, they do so for free. But some will pay and I will vaccinate but others decline. The last community wide exercise was in Nov 2019 and it was facilitated by an organization in Narok.

What are the control measures for brucellosis?

None.

But you said you offer them education?

Yes, we only advise them not to take raw milk.

Which is the severest and most a priority among the zoonotic disease?

Brucellosis is more of a priority because it is common here in humans. And people here consume livestock products and milk products and these animals are sick. Animals show signs through abortions and still births and the pastoralists handle the dead fetuses, they handle the placenta so there is a lot of transmission. I have gotten many cases of people being diagnosed with brucellosis coz of animal handling and consumption of animal products.

Do they know about brucellosis?

No, they don’t know this disease is there in animals. They call it “ugonjwa wa maziwa” (milk disease) but they don’t relate the disease to animals. They don’t trace it back to animals or associate abortions with brucellosis. They milk cows and goats mainly and sometimes sheep. Rabies is second because of stray dogs which are many around here. And also, hyenas are many, animals are preyed on by hyenas and they are bitten by hyenas and people eat the meat from those animals and that is another way that rabies is transmitted to people.

Do livestock routinely interact with wild animals?

Entonet is close to Amboseli and bomas are around the park so they graze animals in the park. So yes, they do.

So, you say they graze together?

Yes, they graze together with wildebeests, antelopes and zebras so cases of MCF are high due to the contact with wildebeests.

Do you sensitize the community about the risks for diseases from this interaction?

They have no choice because that is where they live. We tell them not to graze with wild animals due to Q Fever from antelopes and MCF but they have no choice because they graze together routinely, they have to graze together.

Do they also meet in the watering points?

The place is dry but there is piped water and they have troughs. These are used by wild animals too as the wild animals too are.

Game parks are part of the environment?

Yes, and for the pastoralists they look at game areas as pasture.

Kindly tell me about animal movement?

When it rains then all stay in their area, not far from home because pasture is readily available within. But sometimes they move around in search of pasture. Sometimes they move to Mombassa, Taita Taveta, Kajiado to look for pasture so the animals survive, the animals are moved by young boys because while some go to school others stay home. The girls can go to school but for the boys the father decides who goes to school and who stays. As a result of many boys going to school the ones who move the cattle are boys from Tanzania who don’t often go to school. Right now, elders and chiefs insist that all kids go to school not like in Tanzania. So, these ones are hired because Tanzania is not far from here.

Do the pastoralists move their livestock to Tanzania for pasture?

Not much like it used to happen in the past. After our animals were auctioned in Tanzania because of encroachment people are afraid to take their animals there. They used to cross over but the leadership in Tanzania became more vigilant. In terms of culture, they are all Maasai in both countries but the Maasai in Tanzania are more traditional and conservative because many are not going to school.

Do pastoralists know about brucellosis and rabies so they can report these cases to you?

Rabies they do but not brucellosis. They don’t know about brucellosis; they only know about it when they go to the facility and are told. One farmer came and said many goats were aborting and I told him it was brucellosis. He asked if we had any brucellosis vaccine and I told him there was none. I told him to use penicillin and an antibiotic for his animals. He reported because of the mass abortions in his 300-animal herd. They suspect trauma when they see abortions, like an animal being hit but they don’t think of brucellosis. Rabies they know but not brucellosis.

What about those in remote areas reporting about diseases?

They report to elders and the chief who inform our office.

Please tell me about any collaboration with officers from public health department?

We do because when a rabies case is reported here in the hospital, they will ask us to vaccinate the dogs in the area where the child comes from. So, we are alert but we have no vaccine so we ask the people to be careful. We often don’t have the vaccine. For brucellosis they don’t tell us. Rabies is lethal and is feared a lot more than brucellosis because you will have the 21 injections and you recover. But they don’t know the economic part of it in terms of loss of livestock and work for the sick person. The human productivity loss.

Are there meetings between you and the public health and wildlife officers?

(laughter) No we don’t. The wildlife officer calls me when they want me to follow up and fill the compensation form and for us to write the approximate amount for the animal that is being compensated. We don’t talk about diseases.

Do you feel it is necessary to have regular meetings?

Very necessary because we need to communicate on such things e.g. wildlife officers know if there are any diseases in the park e.g. wildebeest so that we can inform the pastoralists. The nurses need to tell us about brucellosis so that we educate the farmers on brucellosis risk factors from animals.

Would you say that zoonoses are prioritized?

They are not prioritized because I have not seen them as a priority in controlling them. There is no any awareness program that can educate the farmers on TB, Worms and brucellosis. Many people are not aware.

Why are they not aware?

FMD is prioritized because it is the common disease here and affects mobility and productivity of the animal. This is a major problem. For this we have vaccines which are provided by the government and farmers with few livestock they can afford to pay and call us to vaccinate the animals. Then LSD comes second. No one talks about brucellosis and rabies.

Are there any unique challenges in Entonet ward that are not in other wards?

In interaction with wild animals there is a park; Amboseli so a lot of interaction between livestock and wild animals occurs in this ward compared to wards like Kimana. Kimana is in the center so not many wild animals. In Entonet the interaction with wild animals is way higher.

Do you have a schedule for community engagement and sensitization?

We can say (pause). It is random even in the market. I have not seen barazas, I saw them in Ngong but not here. NGOs come and take us to the villages and then we teach all day because the NGO is providing everything. The other times we have no facilitation even all the vehicles are faulty. When there is vaccination the govt facilitates everything but the rest of the time they don’t. So we do it erratically when there is an opportunity and facilitation.

Do you have any question?

What is the aim of this study?

I explained the study and prioritization of diseases at the national level and now we want to know what is going on at the ground. And how to prioritize these diseases and engage local stakeholders.
