# Supplementary material for: Prioritization of livestock diseases by pastoralists in Oloitoktok Sub County, Kajiado County, Kenya
Source: PLoS One. 2023 Jul 12;18(7):e0287456. doi: 10.1371/journal.pone.0287456 (PMC10337939; doi:10.1371/journal.pone.0287456)
Supplement: S1 Data — (ZIP) [file pone.0287456.s001.zip › Oloitoktok transciptions/IDI F 15.docx]

**IDI**

How long have you kept livestock?

I have grown up in a household keeping animals. I keep goats, sheep, cattle and chicken.

Why do you keep livestock?

They help me because they are my source of income. I sell them and buy food for my family. And we keep them for meat and milk.

Where do you graze your livestock?

We graze them in the wild areas. During rainy season they are close home but when it is dry here, we move them to chyulu. The dry season is June to Sep. From Nov-Dec it starts raining then again in March to May. When it rains, they are here grazing in the areas within the homestead.

Who goes to chyulu with the animals?

It is often the young men.

Do livestock interact with wild animals in those grazing areas?

Yes, there are leopards and elephants and lions.

Any harm in their interacting?

The lions prey on the livestock.

Do you ever take livestock to pasture in Tanzania?

No, we don’t.

What are the challenges you face as livestock keepers?

Mainly the animals being eaten by leopards and lions. There are also diseases like olekipei and engeya. Olmillo is very common now and olodua. As well as eriri. (Olodua is like PPR but in cows). And also, oltigana.

What are the signs of oltigana (ECF)?

In cattle is no milk let down and emaciation too, they are not able to walk and also when you milk there is no milk that is what we say it is sick because of milk.

Are there diseases you encounter in the rainy vs dry season?

Diseases are there all the time because they meet in the grazing areas so they transmit diseases to each other. When it is muddy it makes the animal sick and unable to walk so sometimes those ones stay at home.

How do you treat sick animals?

We use teramycin. We only inject them. We mix teramycine with another one drug which I don’t know the name. Some diseases are treated with “hii ta pakiti”. Teramycin is for any disease. We use penicillin for olekipei.

Do you call a doctor to treat animals?

Maybe once or twice and we call them.

When do you call them?

In chyulu hills animals meet from all over so many of the animals get sick and when many animals are sick that is when they call the doctor.

Do you know any zoonotic diseases?

(Pause…Long pause)…I don’t know any.

Are there any risks associated with raw milk consumption?

Only worms no other diseases.

Do people consume raw blood?

People still drink it mainly the men. The blood and the meat laden with blood are consumed by those who don’t go to church but there is no harm.

What about residing with livestock?

We do and the only issue is bedbugs.

Do you assist animals with parturition and do you use gloves?

Yes, we do and there is no risk of disease from this.

Have you heard of brucellosis?

I have heard and people go to hospital, they get the injections and recover.

What causes brucellosis?

I don’t know what causes this disease. I know it in humans but not in animals or what causes it.

Would you like more information on zoonotic diseases?

I would like to know what causes brucellosis…”engeya gule” is milk disease. I would like to know why this disease is associated with milk.

What is the best way to pass this information?

Call for a meeting with the community and we all be educated.

Do you have any question?

What causes the circling disease…the animal takes long before it dies and it has no cure. When we ask the vets, they also say they don’t know they say that it is a worm inside the head.

Do you receive any medication from the vet?

None we just slaughter the goat and eat the meat. This is “engeya ologuny”.

I explain how we will pass this information forward.

I have another question, what causes brucellosis?

I explain about brucellosis at length.

END
