# Supplementary material for: Prioritization of livestock diseases by pastoralists in Oloitoktok Sub County, Kajiado County, Kenya
Source: PLoS One. 2023 Jul 12;18(7):e0287456. doi: 10.1371/journal.pone.0287456 (PMC10337939; doi:10.1371/journal.pone.0287456)
Supplement: S1 Data — (ZIP) [file pone.0287456.s001.zip › Oloitoktok transciptions/Transcripts Oloitoktok H/FGD M 4.docx]

FOCUS GROUP DISCUSSION

Thank you for giving us your time. We would like to have a discussion that will take between 30-45 minutes.

Q. I would like to request you to list diseases that are a problem in this area and as they list I will ask you to write them down here.

A. Okay.

Q. You can list all of them even in the local language.

A. Are we listing only those that attack cattle?

Q. No. Could they list all diseases that attack livestock in general?

A. Oloirobi, engororo, ngutuyanjanget, erriri, kileny, yanerogua, ormilo, orkipei, erisisi, enjoka, engororo, kububo, oltikana, nadomanyet and nunuk .

Q. Is it okay to continue and we will list more as we proceed?

A. Yes

Q. Would you please explain a little further how you determine that your animal is suffering from any of these diseases? What are their symptoms?

A. For engororo the cow produces hard black dung instead of green and has a dry mouth, it attack from the front limbs meaning there will be swelling around there but when it spreads all over and the cow dies it will have blood clot in the veins. When it dies that is when you are certain from the clotting that it was suffering from engororo.

Q. Anyone want to add to this?

A. That is all for engororo.

Q. What are they saying?

A. The nunuk disease shows similar symptoms to engororo for blood clotting though the difference between the two is that with nunuk there is no swelling on any part of the body of the affected cow.

Engororo is dangerous because eating the meat from such an animal can make a human being sick. It just weakens the limbs of the cow. So when a cow dies it is buried deep in the ground.

Q. What about the next disease?

A. Ngutuyanjanget is seasonal around June because it is transmitted by an animal called ngatata that is the wildebeest. Where ngatata delivers, the grass around that area where the placenta falls mostly delivering around February is breeding ground for transmission. So from around May that grass will have grown and the disease will affect around June-July is consumed.

The disease in the grass when consumed attacks the eyes and brain making the cow blind and mad but other parts of the body remains healthy. This blindness may lead to loss of cattle when they cannot find their way back to the home. The prices also go down, when the disease affects many animals the traders know and they will quote very low prices for big cows because they know you will have nowhere to take them.

Q. Anything additional?

A. Yes, during periods of outbreaks, the cattle traders visit villages and purchase even very healthy cattle at low prices because they know the farmer will not want to risk keeping them taking advantage of our problem. We go at a loss especially when you do not have medication for them.

Q. What of the third one?

A. Ormogo which is identified by wounds on the animal’s body. It starts anywhere on the body of the cattle. This disease since old times is believed to come from wildlife especially the rhino. The auspeck type of bird transmits this disease when it eats from the rhino wound then pecks on a cow. But these days we do not believe that because our cattle get attacked by ormogo yet there are no rhinos. There is no medication for it so as farmers we just apply cow dung to the wound or inject the cow with terramycin.

Q. What happens after the cow gets the wounds?

A. It is a wound that does not heal. It leaks continuously like a type of cancer.

Q. The next one?

A. Orkipei

Q. This one attacks goats?

A. Yes. It manifests in the lungs making it difficult for the goat to breathe. It is very contagious. The goat coughs and grants like a human being just like TB. The mucus is so thick that the goat may need surgery to open up the airway.

Another symptom is the goat diarrheas much. When you buy a goat that is infected without knowing it can cause loss to the farmer. This disease is one we manage to control by vaccination and injection. Some respond positively but some die from it.A sick goat will infect others faster if it urinates in the shed compared to infecting others while grazing. Infection occurs mostly at night in the shed.in the grazing field unless they mate infection is not common.

It sounds more like Corona because infection rates are high in crowded places as opposed to then there is social distance.

Q. Any other symptom?

A. The skin looks dry and the goat becomes weak.

Q. What about this eriri?

A. Eriri is a skin disease as well that makes the livestock have rashes all over the body like a snake crawled on it. It is as dangerous as engororo and the carcass is not consumed. It attacks even the eyeballs so once it dies the carcass is buried.

Q. Anything else to add or we move to the next one?

A. Nothing. The next one is kileny. This one starts around the spinal area of the livestock making it weak and bent causing paralysis to the back limbs. The affected animal just drags its hind limbs and since long time ago, there is no cure for it.

Q. What is the next one?

A. Yanerogua. The animal’s blood clots but a farmer will only know once it is slaughtered. During the rainy season when the goat eats the grass, it pumps a lot of blood. A goat may look fat and healthy but is ailing. It is a sudden disease which one minute your goat looks fine and the next it drops dead.

It is advised that when the goats return from grazing, the shed should be let to air out and the goat to get some fresh air before going into the shed to avoid high temperatures which may contribute to this attack. It does not take time to manifest itself, this disease attacks suddenly.

It is a seasonal disease from browsing on ndigiri plant that blooms during the rainy season. There is no medication for it. Some farmers will hastily cut off the ears from the goat once it collapses to allow for blood flow and this saves the goat sometimes.

Q. Have you ever done that and does it work?

A. Yes, but the window is immediate else it dies.

Q. The next disease?

A. Oltikana attacks cows mostly. The grazes on curd but diarrheas, urine has blood, the cow tears in the eyes, weakness and there is swelling on the joints. It develops over time. By the time the cow dies and is slaughtered, the meat is yellow in colour especially areas where there are fat traces.

Q. Do you eat meat from such a cow?

A. Yes but it has to be cooked well by boiling not roasting. Boiling reduces the yellow colour.

Q. Can we discuss the next disease?

A. Nadomanyet is seasonal and attacks goat though mostly sheep with a common symptom of diarrhea. It comes as a result of the animals eating soil in place of salts.

There is a medicine to help stop the diarrhea. When slaughtered, the intestines are swollen and filled with gas and the diarrhea is bloody.

Q. Our next disease?

A. Nunuk attacks around May when it is hot. It attacks cattle. As a cure we inject terramycin and pour ash on the cow as a first aid measure which helps to “cleanse” the cow. When a cow is suffering from this disease it cannot stand when it is cold so we pour the ash on it and expose it in the sun for this to work.

For the farmer to know that the administered treatment is working, the cow urinates. It attacks during hot season and in areas with dust such as Amboseli.

The sick cow should not be given water at all if you want it to heal and terramycin also helps cure it as well as penicillin.

Q. Which other disease?

A. Olerahera is foot and mouth. It affects the hooves making them grow and when they step on a stone it is painful from swelling.

Making the cows in the area with this disease walk on the tarmac road helps harden the hooves making it difficult to contract the disease.

Q. What else can we mention?

A. Engoroto is also caused by a type of fly. The cow loses fur on the tail then the skin looks tight and burnt. The big type of fly is found in Kyullu area or when they graze in the swampy areas like Kimana and Amboseli. It is curable.

Q. What of kububo?

A. this one looks like nunuk because it attacks the joints and skin gets rashes. A cow while grazing just sits and doesn’t get up. It can feed for as long as grass and water is brought to where it is without it walking.

In this area it is not much but it affects some areas brought by ticks.

Q. What of oloirobi?

A. This is a combination of diseases to a point the farmer will not know what to treat. The animal becomes weak, swells up or oozes water-like substances and blood from the mouth, joint pains making the animal not walk. When treated generally the animal could get well. When it attacks, the hooves crack becoming painful for that cow to walk. The mouth also cracks exposing meat and this makes it difficult for the cow to eat.

The cows rarely die from this oloirobi. With the mouth ulcers and poor feeding, they recover best during the green rainy season as opposed to during dry seasons which prolong healing. Terramycin helps in healing but it takes time.

Q. As we conclude this portion of discussion….

A. We have one more called olodua. This one affects the liver making it enlarge and become yellowish and watery on the inside. It is said that when the cattle feed on green leaves, these leaves produce acids that cause this enlarged liver.

Q. This means unless you slaughter the cow one cannot tell the cow is sick from olodua?

A. Yes. No other symptoms show and even after slaughtering it, the only organ affected is the liver and bile. When one dies from olodua, the farmer will go and buy drugs to vaccinate the others as a preventive measure.

And finally there is ormilo which affects the brain and spine of a goat making it loose its senses. Fluid fills these areas it affects and only after it is operated on does one know it is sick from this disease.

Q. As we carry on, from these diseases you have listed, could you please tell me the ones that can be transmitted to human beings from the livestock? Please put a star along the ones the group lists.

A. Oloirobi, engoroto, orkipei when you drink the raw blood of an infected goat and eriri.

Q. How do you think these diseases are transmitted to human beings?

A. By drinking raw milk from an infected animal for example in children we see watery mucus just like when watery fluids from the infected cow then we know they consumed raw milk from it. It is a disease that affects us much because our lifestyle as Masaai is we eat meat and drink milk all the time. It is only spread through consumption of raw milk.

Q. What about engororo?

A. From consuming meat and meat and milk and coming into contact with the sick animal’s blood. We even avoid feeding the carcass to our dogs because they will die. It is a deadly disease. There is no cure for this one for the cows even for humans. It is like a cancer.

Q. What of orkipei?

A. It is transmitted from interacting closely with the infected goats because it is flu-like.

Q. Lastly, what about eriri?

A. These rashes all over the animal are contagious and it can be transmitted through consuming produce from the infected animal. It is deadly as well.

Q. Do you think the close contact of humans and animals contributes to transmission of livestock to diseases to humans?

A. Yes. People react differently to close interaction with animals. Some cough or sneeze just from entering the shed or even may be allergic to the smell of the animal urine.

Q. From the diseases we listed, are there any which livestock contract from interacting with wildlife?

A. Ngutuyantanget from wildebeest and ermogo from the rhino through perching birds.

Q. Of the diseases that human beings can get from livestock, which one needs most attention and assistance?

A. I will say oloirobi because it is there annually and infects many cattle as opposed to the other diseases which are seasonal; I will go with engororo because you go at a loss completely as the farmer cannot even eat the carcass; eriri is a total loss to the farmer as well.

Q. So, do we agree that the one to be given priority is oloirobi?

A. Yes because it affects large numbers of the herds and is common in the area not transmitted from migration.

The second one is orkipei which affects the goats in masses and forces isolation during the outbreak.

Q. Do we all agree on the diseases and modes of transmission from livestock to human beings as discussed?

A. Yes we do.

Q. At any time do you assist your livestock to deliver their young ones?

A. Yes we always do.

Q. Do you think you could contract a disease from an animal through assisting birth?

A. No we don’t.

Q. Who amongst the community easily get these diseases for example young ones, women, Morans or it could be anyone?

A. Anybody can get these diseases.

Q. What is the first thing you would do if someone got a disease like oloirobi for instance?

A. We use traditional medicine called oremiti in cases where the patient cannot get to hospital. This herb makes one cleanse their system making them feel better.

We also give a concoction of soda ash, warm water and lemon for flu-like diseases.

Q. Which would you do first?

A. It depends with an individual. I would go to hospital if someone has oloirobi. For children we rush to hospital but older ones opt for herbal treatment using the bitter oremiti. But if symptoms persist, we take them to hospital.

Q. As we come to conclusion, what preventive measures do you take as pastoralists to protect your livestock from diseases?

A. Dipping the livestock to prevent tick and pest infestation weekly. The government should consider returning the public dip to help the community as they did years ago.

There are few ways of prevention and one has been mentioned.

Giving the livestock a special type of salt to prevent them from eating soil which may causes diseases.

Buying deworming drugs and administering every 3 months to prevent worm infestation to the livestock.

Vaccination of all the livestock.

Q. Does the national or county government come to the community to assist in managing of these livestock diseases?

A. No they do not.

Q. Going back to the list of diseases, is there any that we listed that was not there previously but has come recently?

A. Ormilo which attacks the goats.

Q. Anything additional to our discussion?

A. If the community dip that is like a pool issue can be taken into consideration, it will help the community much. The spray doesn’t help much.

Thank you all for taking time to participate in the discussion
