# Supplementary material for: Prioritization of livestock diseases by pastoralists in Oloitoktok Sub County, Kajiado County, Kenya
Source: PLoS One. 2023 Jul 12;18(7):e0287456. doi: 10.1371/journal.pone.0287456 (PMC10337939; doi:10.1371/journal.pone.0287456)
Supplement: S1 Data — (ZIP) [file pone.0287456.s001.zip › Oloitoktok transciptions/Transcripts Oloitoktok H/KII K.docx]

# KII

Q: Please tell us your name and designation.

A:

Q: What area do you cover?

A:

Q: Do you know how large an area you cover or how many villages you serve?

A: No I do not know because I am newly deployed here.

Q: How long have you been in your role?

A: About 18 months.

Q: And the time you have been in this practice?

A: In animal health from 1988.

Q:What is your highest level of education?

A: I have a certificate in Animal health.

Q: Could you tell us what your job entails.

A: Most of disease control in this area I found is being handled by private practitioners so I mainly do meat inspection because clinical work is mainly done by private people.

Q: So currently you do not do any clinical work?

A: Yes because I found it that way and maybe it could be because of lack of stuff so they engage with those private practitioners.

Q: Are you engaged in any extension services or advice to farmers?

A: of course, when you meet them here at the market someone will tell you I have this problem then you try to solve the problem if it is beyond you then you push it to your immediate boss.

Q: How about disease surveillance, are you involved in that?

A:Not really but if you are talking about disease occurrences then you just get from the farmers then you report but let me say this, of late it has become difficult because even if you report nothing is done so what do you do? In fact you don’t even put it in writing because even if you do nothing is done so you just report it verbally.

Q: In your opinion now that you have been in this area, what are the challenges that the pastoralist face.

A:I would say the first one is drought. You will find that they keep migrating from this area towards Kyulu and Amboseli. Secondly there is this problem of human animal conflict where by you find wild animals have eaten their animals and even elephants trampling on them and of course now when it comes to diseases you find that they just go and pick the medicine and get maybe a qualified person to inject the animals.

Q: So they buy the drugs themselves?

A: Yes.

Q: Do they have traditional drugs that they use?

A: No, I have never seen

Q: You have talked about diseases, can you say they are a problem.

A:Yes because sometimes back we had problems of LSD and they vaccinated ,when it comes to FMD its like it’s a non-issue to them.

Q:In the recent past have you had cases of notifiable disease in the area?

A:You cannot specifically say yes because you have not collected samples so its speculation so like right now there is an outcry of stray dogs and dog bites but since we have not taken samples would we really say its rabies ? You cannot say its rabies but you can say suspected case.

Q:So apart from rabies which other zoonotic disease have your encountered here?

A: Let me say this, I don’t know whether warms are they zoonotic though I have never encountered any that are zoonotic.

Q: You said you also do meat inspection?

A: Yes, that is the major activity.

Q: Do you think the pastoralist are knowledgeable on how to identify sick animals?

A: They are very knowledgeable, not only identifying a sick animal but a particular disease. If it is FMD they know outright it is FMD. They are conversant.

Q: Do you know what they use to identify sick animals?

A: It is just physical examination. If it is FMD.LSD or BQ there are those clinical signs that one is able to see.

Q: When they identify that an animal is sick do they look for help or sort themselves?

A: They sort themselves. I think you have traversed Kajiado. You find that before that farmer comes to you they will have really tried to battle the disease and will come to you as the last resort. There somewhere I correct them because you will find they are giving the animal the right treatment but they don’t follow the manufacturer’s instructions because normally you will find that they don’t get hold of the animal to treat it, they just chase it around and inject so they can’t use the long needle to give an intra muscular injection so normally they give subcutaneous so poor drug administration is a major problem.

Q: So is there any training that you give the pastoralists on such?

A: You just give them when such a case arises and they will spread the word. They are very good at spreading the word.

Q:For the period that you have been here are you able to tell the seasons that these diseases appear?

A:Of course the rainy season there are many cases of BQ,it is all over. I think FMD has no season.

Q:How about any zoonotic diseases?

A:You cannot say zoonotic or rather FMD if you have not taken the samples.

Q:So would you say a challenge is how to confirm ,would you say that?

A:That is the biggest challenge. There was case of suspected rabies, when you tell the farmer we will take samples and tale to Kabete and the investigations will cost you this much the person will be less concern and let it be. They say they can’t use their money. There was another case of poisoning in Mashuru,I took samples and when we told the farmer the cost for the test he declined and at that time there were about 20 animals that were down because of pesticides.

Q: Had they given the animals pesticides?

A: What happened is they after spraying the pesticide the remains in the pumps flowed in a pond and the animals drunk from there.

Q: So that was a problem of environment management, what do you think?

A: Yes and I would say in this area environment management is very poor.

Q: Why would you say that?

A: When you go around this area you will find galleys, soil erosion, there is burning if charcoal

Q: As we are going to finish, is there collaboration between you people in animal health with those in human health, environment to ensure that all are safe and good?

A:I tend to think that is not there. What I can say is that when it comes to meat inspection there is collaboration between me and public health due to issuing of health certificates because they are handling food and also the people in charge of the environment because of the slaughter area so they come in because of pollution.

Q: You have talked about issuing permits. Which is the furthest point that this animals go to?

A:They go up to Nairobi.

Q:Do you monitor where they have come from?

A:They source them locally. You know it’s not possible to tell animals from Tanzania unlike before where they had a mark.

Q:So there is movement from across the border?

A: I understand there is but I cannot say authoritatively that it is there. You will find them saying these animals are from Tanzania but I cannot identify them personally.

Q: When migrating with the animals during drought do they come to seek for clearance from your office?

A: No, you know they migrate as if they are grazing and they cannot come and seek because they don’t know where they are going.

Q: And even when they come back you will not know?

A: You won’t.

Q: Do you think that movement can cause spread of diseases?

A: Of course. Although I cannot say that I have seen but you will find say different type of tick from Kyulu that will cause disease. These people they don’t seek advice and will only come when they have reached the dead end.

Q: And in these area do you think they have prioritized zoonotic diseases?

A: Let me just say Rabies is a concern to them, I cannot say I have heard any complaint on brucellosis and I cannot say it’s a concern to them.

Q:As we finish what can you say about how the pastoralist stay with their animals and the environment.

A:I can sum it this way, we are failing. Years ago we were giving vaccinations of FMD twice a year and a thorough campaign but nowadays we do not do it and it is not a must because funds are not available.

Q:What about how they interact with the environment?

A: You see these people do not usually mismanage their land, maybe over grazing otherwise they don’t cut tree for charcoal, they preserve their environment.

Q: Any other remarks?

A:Let the government provide medicine or vaccines the way it was doing before and that’s the way we can assist these people otherwise without that the livestock industry is almost collapsing and let them also employ technical people.
