# Supplementary material for: Prioritization of livestock diseases by pastoralists in Oloitoktok Sub County, Kajiado County, Kenya
Source: PLoS One. 2023 Jul 12;18(7):e0287456. doi: 10.1371/journal.pone.0287456 (PMC10337939; doi:10.1371/journal.pone.0287456)
Supplement: S1 Data — (ZIP) [file pone.0287456.s001.zip › Oloitoktok transciptions/Transcripts Oloitoktok H/KII J.docx]

# KII

Q: Could you please tell us what your job entails?

A: My work is to rear cows and goats and farming .That is my work.

Q: Do you work with the chief as a village elder?

A: Yes I am a village elder.

Q: What is the name of the village that you head?

A:

Q: How many villages do you cover?

A: Three

Q: How long have you been an elder?

A: I stared when I was 30 years old.

Q: How did you become an elder? Are you elected?

A:I work .You know if someone works people know and see so I did a good job ,people saw and I was elected .You are first elected as a leader in planning for pasture for the animals where they will graze at what times so that they don’t die during drought.

Q: Would you say that is your work as a village elder? Planning for pasture.

A: Yes.

Q: Another one?

A: Even diseases. At times we have many diseases that people don’t know. So many of them come to ask me what the diseases is and what medicine to use. They call me at times to go and see the sick animal. You know this diseases have names and some people don’t know them.

Q: When they ask about the medicine, is it the conventional medicine or the traditional medicine?

A: Both because you know for example foot and mouth if it infects and animal you must inject it with terramycin, at times you take it to a place with salt for it to step on or eat and that helps .

Q: What are the signs of foot and mouth?

A: The mouth rotes and even the tongue and the cow stays for about a week without eating it can even die. Then the foot also gets infected and the cow cannot move.

Q: Do you know what causes foot and mouth disease? Where does it come from?

A: This diseases is the one causing us so many problems. I don’t know where it comes from but every year it comes twice. It comes at the beginning of the year then at the end of the year it will also infect the animals and we don’t know what causes it.No one knows what causes it but it is there all the time.

Q: The beginning of the year is usually dry or does it usually rain?

A: Mostly during the rains. It comes even during drought but it is mostly during the rain.

Q: What challenges do pastoralists face in your area?

A: Diseases. Foot and mouth that is number one. It comes every year and it comes twice a year. When you treat the cow by injecting it, it will be ok for about six months then it comes back again. At times the government brings the drugs and injects them but lately they have not been doing that. When they used to vaccinate every year it was really helping

Q: When they vaccinate do they charge you?

A: They never used to charge but nowadays they charge although it’s not a lot.

Q: So any farmer can afford?

A: Yes, they can pay.

Q: What other problem do they face apart from foot and mouth?

A: Another one is orkipey .This enters goats and cows.

Q: What does it do to the animal?

A: It enters the lungs inside. The animal coughs and it is like pneumonia and the animal dies. It mostly affects the goats.

Q: Do you have problems with pasture?

A: During drought we don’t have pasture and we have to move to go where we can get pasture. At times we go very far but if it rains we plan it ourselves so that they eat in one place and move to the other, like that until the rains come again.

Q: Do all the animals graze together?

A: Yes.

Q: You have mentioned that you go far to look for pasture. How far do you go?

A: We go very far.Do you know a place called Chyulu, we go there. We have forest there with a lot of pasture but no water so when it dry we take our animals there where there is pasture but no water. So everyone takes water to their animals with tanks like this one. We hire trucks to ferry the water there but the animals can stay for about two days before they drink water. We do that until the rains come.

Q:When you go to the forest do you meet with wild animals?

A: Yes, many of them.

Q: So they just graze together?

A: Yes.

Q: When you are moving the livestock in search of pasture, are you given any permits?

A: No, we don’t get any papers .You know at times we have the government parks that we can’t enter but we have our places that border the park and we go there but you know the animals will come there and attack our animals.

Q: Can you say that wild animals attacking your animals is also a problem?

A: Yes, but there is a *Mzungu* who has made a project that compensates when our animas are killed with lions. But here we don’t take the payment because you know the cows are big and are expensive you can get a cow going for 100,000 or 50,000 and they want to compensate a flat rate of 20,000 so we refused and said let it be however if we find the lion eating the animals we will kill it.But we don’t go hunting for them, we like the animals because they help us even educate our children .You know here in Amboseli we have animals and the animals are ours. They help us so much, KWS has a project that give us bursary for our children.

Q:Are there times that you cross into Tanzania in search of pasture?

A:Yes ,even the other day the Tanzanians migrated into Kenya because we had received rain and had grass and we also move there at times.

Q:So if I may take you back ,which diseases give you problems here? You had mentioned foot and mouth disease.

A: You know when Oloirobi gets into animals it will also get into people. You know we milk the cows and drink milk, at times the meat and so the disease will infect people.Orkipey is another bad one but does not infect people much. The third one is Engororo .If it infects and animal it dies and if people eat it they also get infected.

Q:Do you know any disease that can come from milk?

A: I see that is Oloirobi. But nowadays people are more enlightened in the old days they were not boiling milk but now they boil milk and when they do that they are less likely to be infected.

Q: When you look at you animals are you able to tell when one is sick?

A: Yes

Q: What will you look at or for?

A: Mostly I will look at the fur. When the animal is sick the fur will look rough but then you will need to look further to see what disease it has then get the medicine and inject it. At times you will see it has a swollen stomach you with then get a rope tie it to a tree and not give it water for two days after which you inject it and it will get well.

Q: Why do you deny it water?

A: There are some diseases that don’t want water, so you have to restrain it from eating or drinking water and so when you do this then inject it, it gets better and recovers.

Q: What else will you look at?

A: You will look at the eyes and the mouth. You know when an animal is not sick it will chew cud (ngamura) .You will also see the mouth is watery and even the eyes.

Q:So you will look at the skin, the mouth and the eyes?

A: Yes.

Q:So when you identify that the animal is sick what is the first thing you do?

A: I will isolate it, then I go and get the medicine to inject it.At time back in the days when we saw that an animal is sick and even nowadays we would go get the traditional medicine ,a specific tree that we could crush and give it

Q: How will you decide whether to use the traditional medicine or go and purchase from the shop?

A: You know the animals migrate and might be somewhere where there is no shop. So in that case if I have to save the animal then I have to use the traditional medicine. But if they are near we have this drug called Terramycin that we use on them and it treats many diseases. And the white one called penicillin even that is good for animals.

Q: How did you know about these drugs, were you taught?

A: Yes we were taught.

Q: Are there times that experts come to teach you here?

A: They come but not often but you know that someone who keeps livestock if you teach them even ones they will not forget. And it will stay in their head which drug is better than the other.

Q: You have mentioned that there are diseases that can come from animals to human, kindly repeat them for me.

A: I have said Oloirobi,Embururu will also come from animals to humans especially if you eat the dead animal

Q: So what do you usually do when your animal dies?

A:We used to eat ,but not anymore.

Q:Do you burn it, burry it or leave it where it has died?

A: We just leave it there. At time we dig a hole at times we leave it there for the hyenas to come and eat. But you know the Maasai now are educated so we don’t eat but if you go to other interior areas people still eat.

Q: When an animal is giving birth do you help is at times?

A: Yes we have Maasai doctors who help in such cases

Q: Have you heard of diseases that can be transmitted that way maybe by touching animal blood.

A: If the animal is sick it will infect people but if it is not sick then it’s okay.

Q: Still staying with the diseases, if someone gets a disease form animals where will they get help from?

A: They go to the hospital and get tested, if it is oloirobi then there is medicine that they will be given.

Q: And the hospital is near your village?

A: Yes

Q: What is it called?

A:It is in a place called Namelok.and we also have those private doctors who also help and if it get worse we take the person to Oloitokitok ,Kimana or Isineti .

Q:Of the diseases you have told me which one would you give the highest priority?

A: There is another diseases that is very bad. It is called Engati.It comes at its time. In March and April it infects the cows and they die. There is no medicine and even the government has not found one. Now it even infects the goats .There is also another one called Ormilo in goats.It enters the head of the goat and sheep and they go blind .It does not dies immediately but it will die and when you slaughter it you find it’s only the head with a problem.

Q:Do you know where the disease comes from?

A:It comes from the animal called Engati (wildebeest) ,they have given birth now.They give birth in March and they all give birth at the same time. When it rains the grass where they gave birth will grow or if water collects where they gave birth and if our animals drink this water or eat this grass they get sick with the disease.

Q: And you say that has no treatment

A: No medicine yet. But it has killed many animals and it does not discriminate the small or big ones. It will start now from March to April many animals will die. But Maasai have known. When they suspect that their animal has been infected with it they sell them. It is only the head that will the thrown and if you test the meat there will be no other disease.

Q: What will the head have to tell you its Engati?

A: The animal will be blind. It won’t be able to eat properly but eventually it will die.

Q:So of all these diseases that can get to human beings form livestock which would you give the highest priority ?

A:I would say Oloirobi.

Q: Is there any help you have received on this?

A: The government used to help but I don’t know if things have become difficult nowadays. In the past they would come every year to vaccinate the animals.

Q: As we finish, what can you do to prevent your animals from getting sick?

A: There is nothing I can do. Like my animals I have divided them into two. I have some here and the other half is in a place called Irisa.If these are infected you can’t take any of them to the other half so you only mix them when they have recovered. So I separate them. I can also look for the private doctor to go and vaccinate them for me but it is a lot of money.

Q:Are there times that you take the animals to the deep?

A:In the past we had a deep where animals would go in and it was very good ,but now people don’t want that .

Q:Why ?

A: I also don’t know.They made one for us here but people stopped taking the animals.You know at times it’s the cost.

Q: So they charge?

A: Yes.

Q: How much per animal?

A: They charge about 20 to 30 shillings and people have to do the math. If you have about 50 or 20 cows you can buy deep at 250 shillings and you spray with a pump instead of being charged 20 or 30 shillings per animal which is more expensive .That is the reason.

Q: How about vaccinations?

A: They don’t come every year.

Q: So if the vaccines are brought you would take your animals?

A: Yes, and you see that one is not expensive, it’s much lower compared to the private ones.

Q: So the farmers can afford?

A: Yes

Q: Thank you very much for your time.
