# Supplementary material for: Prioritization of livestock diseases by pastoralists in Oloitoktok Sub County, Kajiado County, Kenya
Source: PLoS One. 2023 Jul 12;18(7):e0287456. doi: 10.1371/journal.pone.0287456 (PMC10337939; doi:10.1371/journal.pone.0287456)
Supplement: S1 Data — (ZIP) [file pone.0287456.s001.zip › Oloitoktok transciptions/Transcripts Oloitoktok H/KII M.docx]

# IDI

**Q**: What is your role as the

**A**: My role as the is coordinating health services in this sub county and to ensure the services are running well in our sub county and addressing all issues that pertain to health issues affecting human beings and all survey studies regarding reproductive health and general public health conditions, running the administrative services in the facility and rural health facilities in this sub county.

**Q**: Are you also in charge of disease surveillance in this area?

**A**: Sometimes, though we have a disease surveillance coordinator. We have divided ourselves into various sections each with its own coordinator. We have coordinator of disease surveillance, we have coordinator of public health so we have coordinators of various units in the sub county so I am just overseeing all of them, and the units are coordinated from office.

**Q**: Do you have people who educate the public on health issues affecting the society?

A: Yes, we have health officers on the ground ranging from CHVs who educate the people regarding the health services available in their areas, they also conduct something called community health action day, community dialogue day when they see that there is an upsurge of a particular health problem in their community .They just gather the public or the villages that they are representing and inform them of what is happening in their catchment area where they serve then they tell them of the condition that are there and what they need to do to reduce this problems .We also have community health assistants that is the CHAS who supervise the CHVs in different community health units in various villages attached to the nearest health facility maybe a dispensary ,a health centre or a hospital .With them we also have a public health officer coordinating with the CHAS who supervises them and helps in coordination to ensure that the community services are taken care of in their villages in sanitation ,access to clean safe water ,proper nutrition and all other health issues in the villages.

**Q**: What are some of the examples of solutions that people give during dialogue day?

**A**:For example if there is a problem that they have identified ie the CHV and CHA in a certain village is say there is a high rates of malnutrition maybe because they have no access to all food groups they suggest to people to do kitchen gardens to grow vegetables , they suggested that people rear chicken to supplement their protein source from eggs , practice zero grazing to be able to get milk. At least they can be able to come up with the solutions themselves that they are able to do at their local level

**Q**: How long have you been in this locality as the M.O H?

**A**: I have been here for around 18 years.

**Q**: For the period you have been here, what would you say are some of the zoonotic diseases that people suffer from or that are reported in this area?

**A**: The cases that we have seen that are at least linked to zoonotic diseases what we have seen commonly is Brucellosis .This is what we have seen several times in this area.

**Q**: How many cases do you receive on average in say a month or six months?

**A**: Because we get our reports every month, I would say an average of around ten cases in a month from our catchment area although in some areas we don’t have laboratory services so they wil just treat them clinically without isolating to confirm if it is a brucellosis case, we have very few laboratory services in this sub county so the cases that we are sure of are the ones that have been confirmed by the laboratory.

Q: Are there areas that report more cases than other areas?

A: No, at least each area has a case. It is just spread all over

Q: Are there seasonal variations on the disease?

A: We have never analysed it in terms of seasons because this has never appeared as a priority to us to dig in to find out. Maybe we have left it to the veterinary people, we have not laid emphasis on it.

Q: From your experience, when someone has a zoonotic disease or any sort of infection, is the hospital the first place they seek help?

A: The hospital is usually the last resort since their first option is always traditional treatment unless the situation is crucial. They usually start from the herbal, the traditional treatment. Those who come to the hospital first are the other non Masai communities but the natives of this area start with the traditional herbal medicine but when things become worse they now come here when they are very late.

Q: During your disease surveillance, is there a collaboration between you as the MOH , the veterinary and wildlife department?

A:Yes I can say collaboration is there when we have issues touching all of us although the collaboration is not that strong ,it is in a very small way and it is upon us to take it further but we have not taken time to interact and have a discussion together. We had a group from another University came to do a study in the area and that is when we actually met and exchanged the health issues and veterinary issues ,how we need to collaborate together to assist the communities in these conditions .The collaboration is there but it is not well coordinated.

Q: Have you ever received any training on one health?

A: Not yet but I know a bit on it.There was a time a group of public health students from Moi University, University of Nairobi and other African countries apart from Kenya came to educate people on one health . They went to Amboseli area and did a brief introduction but it was not a proper training on one health.

**Q**: Do you think training on one health is important?

A: Yes, it is very important.Infact I would love that collaboration to be strengthened and also have a link with them so that we can be able to exchange ideas and solutions because we have seen zoonotic diseases coming to human beings even the Covid 19 source is the animals to human beings so that means we have to strengthen that collaboration to ensure at least we are well equipped both sides..

**Q**: Are there any diseases you prioritize over others in your facility or in the area that you oversee?

**A**:Yes,when we get morbidity data from various facilities we rank them from top ten diseases ,top five conditions in our sub county so our top ten priority ranging from respiratory conditions , diarrhoeal conditions, hypertension, diabetes, however, diabetes has risen,it is a life style problem

Q:Is this the case even among the natives?

A: Yes, now it cuts across. it is even affecting the natives. We then have skin conditions, eye conditions , road accidents motor bikes are so many so we get so many road accidents , pneumonia, burns though burn cases comes with season, when it’s cold we get many cases but not when its warm.

Q: Is brucellosis as one of the zoonotic diseases you have mention a priority ?

A: It has never appeared among the top ten but in the top twenty.

Q: Why is this so?

A: This is because the locals have not taken into consideration on vaccinating their animals, properly boiling their milk before consumption and keeping a reasonable distance between where they sleep and the animals. This can nonetheless change if they receive the proper education and sensitization.

Q: Since you mentioned that they have their own solutions could that be a factor?

A: Yes that can be a subject of discussion with them, community dialogue because if that condition is in their community it then needs to be brought forward for them to be told where the conditions come from

Q: As we conclude what are your views and final comments regarding on the situation of health and the zoonotic conditions in this area?

A: My recommendations or my feeling is that it is high time we need to collaborate and work together with the veterinary and agriculture officers because our services and what we need to tackle in our fields are related and I look forward to these collaborations for us to work together and share our problems and make the lives of our community better .
