# Supplementary material for: Prioritization of livestock diseases by pastoralists in Oloitoktok Sub County, Kajiado County, Kenya
Source: PLoS One. 2023 Jul 12;18(7):e0287456. doi: 10.1371/journal.pone.0287456 (PMC10337939; doi:10.1371/journal.pone.0287456)
Supplement: S1 Data — (ZIP) [file pone.0287456.s001.zip › Oloitoktok transciptions/Transcripts Oloitoktok H/IDI M 1.docx]

# IDI

Q: What are your full name?

A:

Q: Which ward is this?

A:

Q: Which village?

A:

Q: How old are you?

A: I am 54 years old.

Q: What is your highest level of education?

A: I have never gone to school.

Q: Which religion do you practice?

A: Christianity.

Q: For how long have you been practicing livestock farming?

A: Since childhood.

Q: How did you acquire your livestock?

A: The first cows were gifts from my parents but with time, I bought my own.

Q: Apart from cows, are there any other animals that you keep?

A: I also keep goats and sheep. At times I finish them because of school but I start keeping afresh

Q: Which animal do you prefer the most between cows, goat and sheep?

A: I prefer all but currently where I am living, keeping goats is a hard task because of limited space. Goats are good even their prices are good but because of limited space I now keep only cows and sheep. I love all of them but I don’t have the ability to take care of them.

Q: What is the importance of animals to you?

A: They are very important to me, Personally I have not gone to school and if one is not educated you cannot find employment and even if you get employed it will be a very low job we therefore focus on your animals that can help us because you can sell and take your child to school, you can get milk and that is why cows are very important to us.

Q: Can we therefore say that your economy depends on the animals

A: Yes although I do some farming but animals are number one

Q: Where do you graze your animals?

A: They just graze around here because I don’t have children to take them grazing in the forest I am therefore forced to graze them close by. The cows are not many because the areas to feed them have become small and if you let them go far you will see they won’t be good.

Q: What do you mean by this?

A: I mean they tend to lose weight and also there will be a decrease in the milk production too when animals go far even if they are going to graze and even when there is a disease outbreak the animals that go far will be severely affected but those that stay around will be ok.

Q: Are there times you take your animals grazing far from home?

A: Yes, but currently I have a manageable number and there is really no need for that unless when it gets very dry then I will have no option but to move them in search of pasture .And you see if you have say only five cows you do not need to move them as you can comfortably stay with them but if you move them then you will go at a loss

Q: What are some of the challenges you face in this area as a livestock farmer?

A: We face challenges like diseases .The diseases have increased .There was a diseases that was referred to as *homa ya ngombe*, the number of cases has really gone up and this is a problem. The cows are always being injected.

Q: Is pasture a problem?

A: Yes it is a problem because pasture available has reduced but we just try and make due with what is available, if you have a small number of animals you can even decide to buy fodder for them

Q: Which livestock diseases are causing you problems in this are?

A: We have several diseases, we have the disease that enters the feet, we call it *homa ya ngombe* (*Oloirobi*)foot and mouth this severely affects the cows especially the limbs as it even cracks the hooves and even the mouth. Another one is contagious caprine pleuropneumonia (CCPP) (*Orkipei* )appears like rashes mostly kill goats.We also have enterotoxemia disease (*enairowua)* that usually appears in rainy seasons it also kills goats. Then we also have Lumpy skin disease (*Enarirri*) which is very bad disease, there was an outbreak last year and it affected very many animals and killed many of them. In the past when we had disease outbreaks the veterinary doctors would come very fast and treat the animals but that is no longer the case we don’t know what changed.

Q: From the diseases you have mentioned, which ones come in the rainy seasons and during drought?

A: There is one that I have not mentioned that comes during drought, it is called bovine fever( *nunuk)* and it attacks the animals because of the dust. It come together with trypanosomiasis (*engoroto*) and if it comes and you fail to notice it and by bad luck the animal drinks water then the animal will die instantly.

Q: What about during rainy weather?

A: In cold rainy seasons the most common is foot and mouth ( *oloirobi)* spreads in cows especially when people bring animals from the market . Another one that comes from wild animals is malignant catarrhal fever (*inkutie oo nchangit*) it affects the head, the animal looks ok but it will eventually die. There is another one called coenurosis (*Ormilo*) in sheep and this one we have not found a cure for it, the only solution is the knife that is the other bad one as it continues to finish our livestock.

Q: Do you know which wild animal transmits the disease to your livestock?

A: In most cases the Maasai believe it is the wildebeests. When it gives birth and a cow grazes there then it will get the disease so it comes mostly when the wildebeests have given birth.

Q: Do you think there are diseases cows can contract while grazing in other areas?

A: Yes, like the one from wildebeests and foot and mouth. You can also go to the market and buy and an animal that is already infected without knowing and when you bring it home it will infect your animals so we just try and treat them.

Q: Do you think there are areas that have diseases that are not in this area or does this area have diseases that are not found in some other areas?

A: Yes, there can be an outbreak in one area but it will eventually disappear. In the past the Maasai were few and they lived in the forest and if an outbreak was noted in say a goat from Sineti they would be blocked there and would not even be brought to the market but those days are gone..

Q: How can you tell when your animals are sick?

A: You can tell, you will see the animal is dull, it has a rough coat, it will be just standing not no appetite and if it is a cow producing milk it will have low milk production.

Q: What is the first thing that you do once you have identified a sick animal?

A: I will inject it with terramycin.

Q: Do you do it yourself

A: Yes

Q: Where did you learn the skill of injecting animals from?

A: We learned as we were growing up.

Q: Are there any traditional methods of treatment you can use?

A: These days people don’t use them however in the past when we were growing up people used to boil Aloe Vera (*esukrei)* and cured their cows of ECF but nowadays I don’t see that practice ,even the plant is hard to find .

Q: Do you receive any help with the diseases from the county government or health officers when there is an outbreak of a disease?

A: In the past they would come and vaccinate the animals when there is an outbreak but nowadays they don’t come ,it has been long.

Q: Do you know if there are diseases that can come from animals and infect humans?

A: Yes. If an animal gets LSD(Enaririi) Maasai don’t eat it because they say if you eat it you will die ,when the animal dies it is buried because we know the diseases is bad and also when there is an outbreak of FMD(Oloirobi) people also get it because they use milk but people from this area now boil milk because they know that if they use without boiling then they will get infected . Usually caused by eating meat from an infected cow.

Q: Do people still carry out cultural practices like drinking animal blood?

A: Not all.I am a Christian and a pastor so we believe it will be like drinking human blood.With us when a cow dies without being slaughtered, we cannot eat that meat.We say blood is life ,we have come to know this through teachings of Christ ,we say the blood of Christ was poured to save us and so we say that is life and we shoul not use it however in the past before we were born again we would pierce the neck of a cow and drink so now we teach people not to do that although we still have a few who still practice that .

Q: Of the diseases that you have mentioned as zoonotic, which one should be given priority?

A: You know any disease that comes should be dealt with at that time, all those diseases should be dealt with because we don’t know what to do .When we have LSD we don’t eat the animal and that is a loss so if there is an outbreak it should be dealt with and even when we have FMD children are rushed to hospital and even adults go to hospital to seek treatment because people say the animals have *homa* that will infect them .

Q: So between those two which one is higher

A: FMD is always with us. You may not have it here but when you go to Amboseli you will find it or Birikani, so it’s just going round never goes away but LSD just comes with time.Oloirobi should be given priority because it never goes away and the one affecting goats CCPP( *Orkipei*)

Q: Do these diseases like FMD and CCPP affect the prices of the animals when you decide to sell them?

A: Yes, we get losses because when the animal has FMD no one will buy, you can’t take them to the and they will also lose and also when we have LSD it is very bad

Q:In the past year or two have you had cases of LSD in this area?

A: Yes ,we had it last year .We washed the cows in the deep because people were scared and the drug used in the deep was very good ,we have tried o look for it but we cannot find it ,I think its only found with the government so we just try to use the drugs available in the market but they are not as effective ,when I washed them with the one that was brought they never got LSD even FMD because that drug was very powerful compared to the ones that we buy which are not as effective

Q: What preventive measures to you take to protect your animals from diseases?

A:You know ticks cause diseases so you must try and wash the animals like every Saturday to get rid of the ticks ,so in my opinion washing the animals will help prevent diseases.

Q:Is there any other way or method that you use

A:When we see that an animal is sick we inject it with medicine immediately

Q: How about vaccinating them ?

A:We only had vaccination in the past years by the government

Q:So you only depend on the government for vaccination

A:No that was in the past, we were sure vaccines would come but nowadays they don’t

Q: If there was some form of training or sensitization about diseases that is to be brought in your area .what would you like to be educated on regarding livestock diseases?

A: I would want to know about the diseases, where they come from and the solutions and ways to follow.

Q: How would you like to receive this information?

A:Apart from the cost ,seminars would be the most effective because people will get a chance to ask questions but on the radio it is announced and it just passes many will not take it seriously and if you put poster many will see but just pass, I have seen that our people get more from seminars

Q: Have you been affected by FMD in the past 6 months?

A: Yes, even the other day some cows were affected and one still has not really recovered well.

Q: What is the first solution when someone falls sick in your household?

A: Take them to the hospital.

Q: Do you use traditional treatment of any kind?

A: We have them .We use them only when the person has not gotten very sick .We have medicine that we use but only if you have not gotten sick maybe you have stayed or long without releasing bile (*nyongo*) so you use the drug and vomit or another that will make you diarrhoea but if you go to the hospital you will get better faster but when someone is very sick and has no strength we rush them to the hospital

Q: Do you have anything to add on what we have talked about?

A: What I can add is that you know how the situation is now .The government should try assisting the people and see how they live since our animals also contribute to the economy
