# Supplementary material for: Prioritization of livestock diseases by pastoralists in Oloitoktok Sub County, Kajiado County, Kenya
Source: PLoS One. 2023 Jul 12;18(7):e0287456. doi: 10.1371/journal.pone.0287456 (PMC10337939; doi:10.1371/journal.pone.0287456)
Supplement: S1 Data — (ZIP) [file pone.0287456.s001.zip › Oloitoktok transciptions/Transcripts Oloitoktok H/KII A.docx]

# KII

Q: What is your name ?

A:

Q: What is your designation here?

A:

Q: In which Sub-county?

A:
Q: Which area do you cover?
A: I cover the Ward level.

Q: Which ward is this?

A:

Q: How many village does this Ward cover?

A:

Q: For how long have you been holding that position?

A: I have been in the position since 2018.

Q: What is your highest qualification?

A: B. sc. in animal health.

Q: What does role entail?

A: I deal with general animal health including controlling diseases for example when there is any outbreak we vaccinate animals and even when it comes to treatment we assist farmers and also in terms of markets we issue movement permits, I am also in charge of inspecting meat around this area of Oloitokitok and Rombo and extension services.

Q: Which extension services do you offer?

A: Generally animal health and production because it is part of the disease management.

Q: Do you train any of the pastoralists?

A: Yes, when we are doing extension we normally teach them various skills, mostly during chief barazas we organize and talk to them concerning animal health.

Q: What about disease surveillance?

A: We also do disease surveillance.

Q: In your experience of the years you have been here, what would you say are the challenges these pastoralists encounter most?

A: They mostly encounter problems of drought because sometimes rain becomes erratic, they then migrate with animals to other areas and face challenges such as other diseases from that area and lose many animals. When they go out looking for pasture they return with foreign diseases back to this place. So those are the challenges we see. Generally, they take their cows for dipping and they try to wash them although when it comes to Deeping their animals you know its spraying and some of them have very big numbers of animals and the way they would be able to tend to each animal will be minimal in terms of spraying them so they will not e able to cover the animals properly so tick borne diseases becomes a challenge.

Q: When you say movement, do they move to neighboring Tanzania or do they just move within?

A: Yes, they even move to Tanzania. You know the Maasai of Kenya, especially the ones from Oloitokitok, are related to the Maasai of Kisongo, the ones who are across, actually they are relatives. Unfortunately, sometimes because of the international boundaries, the Tanzania government confiscates their animals. Some time, back they crossed the border and their animals were confiscated because when they were entering Tanzania. They entered through the areas that are inhabited by the Chagas who are farmers so there was commotion because they had taken their animals to feed on the few grass they had. The ones who are near Namanga go to their villages. Sometimes they cross to Tanzania but mostly because of boundaries issue, they just cross inter county boundaries. They can go to Taveta and even Makueni; you can hear them going to Kwale as well. They go very far.

Q: Do they also need permits when they move locally?

A: A bigger population will move without because some of them do not know if they are supposed to get movement permits. In addition to that, the distance where somebody is matters because one might be at a place called Otasika or Intilal. He sees that coming all the way here to seek a movement permit for going to Mombasa if they are going to Mombasa will take a lot of his time and the animals are dying because of drought. Therefore, this person will risk it and go without a permit. Although, there are a few who will go to the veterinary department to seek permission from the other side so that it can facilitate the movement of our animals. These ones put their animals in vehicles and take them through the road. Nevertheless, those who do not have permits use shortcuts and when they reach Taveta, they are automatically residents of that area. Generally, I would not say that they do not know they are supposed to have permits. Unfortunately, the way they are cornered by the situation fools them to either go without permits or come to the office depending on how they understand. For example, there is a man here in Ndarara, who was taking his cows to Kwale, he told m and we called the veterinary doctor in Kwale who gave him a green light. I wrote him the permit and he took his cows even now some of his cows are there.

Q: Which are the common diseases affecting them in this area?

A: Mostly Anaplasmosis particularly the lower belt, cases of upper belt are rare but have ECF. Cases of heart water and Babesiosis are also common. I remember there was a time I went to attend to some animals that were infected with Babesiosis because when I injected them, they responded very well. Some other cases like ephemeral fever are rare.

Q: What has been the recent notifiable disease in this area?

A: There is a time we had Foot-and-Mouth and Lumpy skin Disease. We conducted some vaccinations and it was all good.

Q: How do the pastoralists respond when you are doing a vaccination?

A: They appreciate so long as you inform them on time because somebody could be residing here but his animals are in another area. Therefore, if you do not give him adequate time he may not be able to bring the animals because he needs to coordinate properly with the person taking care of his cattle and sometimes there are no phones to communicate. Thus, for these programs to take place, we tell them in advance through coordination with chiefs and the elders of the village. We tell them to mobilize their people and when they are properly mobilized you find it being okay. Sometimes, the charges also matter as we mostly charge 20 shillings per cow for the one organized by the county, however, some of them complain that it is too much and ask for a reduction. This is mainly affected by the season and timing because sometimes you find that in the market the cows are not being bought therefore complain that they cannot even sell one cow to take care of the others. It is not that they do not want the vaccination they lack the money. When marketing is doing fine, they respond very well. This clearly shows that it mostly depends on the time and season, for example during the drought season you find them being reluctant because there are a lot of problems such as scarcity of water and pasture, attacks from diseases and needs at home among many others.

Q: In the last three to six months, have you encountered any zoonotic disease?
A: You cannot say we have not encountered because things like hydatidosis in animals are there. I have not seen cases of brucellosis of late. However, in this area, when I tried to inquire from the ministry of health, I would find cases of brucellosis that have been reported to clinicians. Hence, generally I would say that although it may not be very common, if you follow up you would find people who have contracted it. Nevertheless, for the last six months cases like hydatidosis and fascioliasis have been seen but not rampant. In damp areas, cases of bilharzia are not a threat because of the good drainage system in the area.

Q: Does the drainage system contribute to bilharzia infection in the area?

A: Yes. This is because stagnant water provide good breeding grounds for snails and fasciola species because the life cycle of schistosoma must go through the snail and fluke.

Q: Do pastoralists call you when they have problems with their animals?

A: Yes.

Q: Does this mean they have a way of knowing when their animals are sick?

A: Yes. These farmers have stayed with animals for a very long time, especially the Maasai community because they are the majority here. Most of them have been brought up in homes rearing animals. Therefore, in terms of ethno medicine, they even have some herbs, which they boil for the animals, and they respond well. However, when they have tried their herbs and failed, they now try to look for the veterinaries. This is the only time they try to call you. They will not call you before they challenge that thing on their own since they mostly start by trying to challenge it. Most of them have these terramycin,adamycine,streptomycin at home at their homes, will first try to control the situation, and will only call for assistance when the situation is only worsening.

Q: Does this mean they try to cure the disease on their own?

A: Yes, because of the history and vastness of this place. For instance, somebody who is 60 to 70km away, when the animals come in the evening and notices one of them is not well, the first thing they do is giving the animal the medicine they have at home. When they see that the medicine is not working, they now look for our numbers. Although this only happens occasionally because other times when they try and it does not work, they look for help from the health department. They now come to us and we advice them properly on what to do. Generally, they try solving the issue first before coming to us.

Q: How are the seasons here? Are there seasons with more diseases than others?

A: Now we are approaching the rainy season, the drought season this time was not that bad because we had rains in January. We are normally supposed to have long rains in this area from November to December.It was not too much but it rained again in January. As you can see, we have maize and food in plenty. Generally, this is the time we have everything including the ticks and whatever. They must increase in number because of the favorable weather. However, sometimes they have a hard task attacking the animals because they are healthy hence, in good shape to fight them. Mainly during drought, when animals go to drink from the communal watering points because their owners lack enough water to give them, they end up infecting each other with diseases. This happens because if someone’s animal had FMD, it ends up infecting the other animals when they take water. When the drought season is starting causing scarcity of water and pasture, the focal point where they meet they end up infecting each other. Therefore, it is safe to say that during drought many diseases are seen spreading when the animals go to common areas looking for pasture and water. This is the time where cases like foot and mouth, lumpy skin, CBPP among others are very common. Areas along Nairobi experience long rains unlike this area where we have short rains. This means if we do not get rain now, there will be scarcity around July hence drought towards November. Therefore, these animals will migrate to a common area in search of pasture and water including the ones from Namanga area and will meet at Chyulu. This is the time they interact and spread diseases to each other. If the county government does not organize vaccination programs now before the drought that is July then cases of animal deaths from diseases will be high because of outbreaks of diseases.

Q: In your own observation and experience, which zoonotic disease would you say is very severe in this area if you were to rank them?

A: Hydatidosis, although normally it will be seen affecting food animals, I would say it is more common because we normally collect data when inspecting meat and we will say the liver has been condemned because of hydatidosis, the lungs and those things so I would say it is more common . Issues of brucellosis sometimes we normally say that when we are observing you will find signs of it. You will not always find all the signs, there is only one time though in the past I had observed a sign for another disease and I thought it was it. Generally, you will not find those signs very much but when you go back to the human clinicians and ask for data for Brucella, you find it in plenty. There was a survey conducted by doctors from The University of Nairobi and they found presence of brucella and had spread all the way to Chyulu. They found that cows have it but in terms of prevalence, Hydatidosis is more common. Brucellosis based on the clinical record, is also there but I would say since it is found in the animals, we deal with it before human beings contract it. Although, the one affecting people a lot is brucella has been reported and is on record. Cases of rabies are rare and the people are rushed to hospital before it gets serious. Sometimes when we have adequate vaccines, we conduct rabies vaccination. Rabies, brucella and hydatid are the most prevalent ones. People are aware of it and hence takes measures to avoid it by boiling milk before drinking. Issues like teaniasis are not very common in this area. This might be because of long-term exposure to public health education in terms of disease control. You rarely find such when you are inspecting the animals and it can take a long time before you actually see one. In addition to that, people have actually taken the initiative of building toilets in their homes and not going to the bushes. Even if they go to the bushes, so long as the meat they are eating is clean, you do not expect them to have teanisis. I would say teanisis is not a big problem in this area in terms of animals. Maybe after proper correspondence with the public health and clinician’s records, you can actually know the prevalence of teanisis within the sub-county. The four diseases I have mentioned are the ones that might be an issue to people here but teaniasis is not a big problem. I can rank Brucella the first as a threat followed by Hydatid.

Q: You have said that if I want know the cases of brucellosis, I have to check clinical records correct?

A: Yes because we do not have those tests.

Q: Do you as the animal health expert have a collaboration with the human health experts or everyone focuses on their own area?

A: The collaboration is there. This is because if we find a case we are suspecting we normally collaborate with them because of issues of one health approach. If I find a situation that may affect people, I will work with my colleague there and inform him what I am finding. Some of these tests cannot be done here because our lab is not updated like theirs they have facilities. They will be able to know quickly when it is in humans because people will go there and they will take samples and conduct the procedural tests. For us when we have any tests we send it to Kabete or Athi River, where there is a lab there.

Q: So there is collaboration?

A: Yes, if I find a case that I should report to them I normally inform them.

Q: Do you have anything you might want to add in terms of how the one health approach can be improved or in your daily operations, is the collaboration perfect with the environment, health and livestock?

A: The most important thing because the people were aware is that one health approach has been adopted worldwide. This means the people who are dealing with animal health, human health and environment focus on one health. When they are dealing with a problem, they will deal with it together. Therefore, I would say there is communication for instance, if I have a problem here I would contact my colleague there and tell him to alert the superiors in charge. There was a time we were doing vaccinations. We had people from environment, although we were in the same team, the vets would be ahead and start vaccinating the animals. People from public health were also behind us, we had environmentalists who were coming to see how we were conducting ourselves. For example, if we wanted to use the washrooms what we would do. This is because ideally, we are not supposed to go and start polluting the environment when we go to vaccinate the animals. Instead, we are actually supposed to be quite sensitive to the environmental issues therefore should not pollute more but leave it better than we found it. The needles we were using we were supposed to be very careful when handling. We had containers where we would put them after using because if someone were pricked, it would cause severe injuries. There are also environmental issues; some people from the environment health do a follow up although sometimes the farming in the area normally harbors it. I think the situation in that area should be improved in order to better conduct the study in the area. One health approach should be facilitated more in terms of funding. This is because if you have to move far to go and start conducting a vaccination somewhere and you need somebody from the public health, environment, vet or another social worker elsewhere then you need to be properly facilitated in terms of finance if you have to move together. For instance, covid-19 has really changed things. Now, you have to be properly facilitated with the vehicles. Back then, we used to sit near each other nowadays you it is impossible because of social distancing. Therefore, if a vehicle used to carry seven people in the past, nowadays it carries only four people. Hence, funding must be accounted for because of how things are due to covid-19. From the example of the car, funding should be increased since the cars used need to be added because we have to accommodate those people and the equipment we use for the job.
