# Supplementary material for: Prioritization of livestock diseases by pastoralists in Oloitoktok Sub County, Kajiado County, Kenya
Source: PLoS One. 2023 Jul 12;18(7):e0287456. doi: 10.1371/journal.pone.0287456 (PMC10337939; doi:10.1371/journal.pone.0287456)
Supplement: S1 Data — (ZIP) [file pone.0287456.s001.zip › Oloitoktok transciptions/Transcripts Oloitoktok H/FGD M 3.docx]

# FGD

Q: I would like us to list the names of diseases attacking cattle in this area in any language

A : Engoloen, Oleroro, Unuku, Isuro, Ingenerubo, Ndoigiri, foot and mouth and sleeping sickness.

Q: How do you know that your livestock are sick? What aspects do you look at?

A: In your opinion, are these the common diseases that affect your livestock?

A: Yes, those are the common ones

: Firstly, you look at the skin, how the fur is raised, sometimes you can look at the hooves because some diseases infect the livestock through the hooves, the eyes and mouth and nose; the livestock may have mucus, a watery mouth or eyes, livestock become lazy, they don’t eat, they just sleep, livestock urine; It doesn’t urinate

Q: When you see these signs on your livestock, as a livestock keeper what is the step you’ll take?

A: Report to an elder, for example, if a child was looking after livestock, when he gets home in the evening, the reports it to his parents and medication through injection will be administered by the farmer

Q: Who will administer the medication?

A: We do it ourselves

Q: Where do you get the knowledge to inject a livestock with medication?

A: From our parents. We do it ourselves because when we were growing up, our parents taught us how and this knowledge is transferred from parent to children so I got from my father and my son will get it from me. There is a syringe; we buy medication

Q: Do you know the type of medication you supposed to administer to the cow?

A: Yes, we do

Q: How do you know about the type of medicine to administer to your livestock?

A: You look at the signs to determine the type of medication to administer to the livestock, for example, if it has signs of trypanosomiasis, you administer tryponosomiasis medication, if there are signs of a flu, you administer medication for a flu.

Q: Did you get all this knowledge from your parents?

A: Yes, the knowledge comes from our parents and sometimes, but sometimes when it is complicated, when you go to buy the medication at the agro vet, you describe the signs that the livestock as to the agro vet attendant. The agro vet tells you what that type of diseases. However, in the past years terramycin was the common medication. This is not the case as nowadays we come across very many types of medication but we get help from people who have gone to school on the names and the amount of mcc according to how it has been prescribed

Q: So when you buy medication, is it already prescribed?

A: Yes.

Q: When you go to the agro vet, do you explain the signs of the disease or you already know which medication you are going to buy?

A: You go when you have decided the medication you want to get

Q: You say you have medication in stock?

A: Yes we do. These are for emergency cases and sometimes the medication cannot be found here we store them at home. In the evening, when a cow portrays some signs of sickness, they are given an injection. For example, every livestock keeper has two types of medicine; ademycin and terramycin. If the sickness is complicated, medication is bought from agrovets. You explain the signs to them. However, there is no livestock keeper who does not have ademycin in his house. This is because the common type of disease in goats in this area needs these two types of medication.

Q: At what point do you decide that the medication that I have is not working?

A: There are complicated diseases which the doctor can tell us about; discourse fever caused by ticks. This disease cannot be recognized easily. Sometimes you use ademycin, the symptoms persist. You use Novidium but there are no changes, Udrelpen and still no changes. When this disease persists, the cow starts developing a foul smell from the skin. When you have administered medicine to the cattle and it is not working and the cattle generates foul smell, then you can look for medicine for oltikana because it’s a rare medicine and the disease is also uncommon. In case of no luck then head to Oloitokitok. In Oloitokitok there are agrovets and there are some who are retired veterinary officers so you just explain it to them. If he tells you it’s oltikana then he will help you look for medication if he doesn’t have. However, some people apply ashes all on the cow’s back.

Q: So that is the traditional method?

A: Yes, this disease is common when there is a lot of dust and residual rain water

Q: In what season is the disease common? During a dry season?

A: It is common during the dry seasons especially after a rainy season mostly in December and January when it is very hot. This happens when the cattle drink stagnant, overstayed residual rain water. We believe that when a cow drinks this water, the water causes Red yellow fever.

Q: Do you cover it when you sleep or what do you do?

A: You tie it in a ‘mogo’ so that it moves from the cattle’s muscle to the ’ mogo’, the mostly used medication is covering the cattle with ashes for almost two days then they will recover on their own.

Q: Among these diseases, are there some that are transferrable from the livestock to human being?

A: Yes, possible number 3: Orkipei, and also number 6: Engororo

Q: Is there any diseases not listed that is transferrable from livestock to human beings?

A: Orkipei, oloirobi, engororo, enkurukanjangit

Q: These ones that are starred are the ones that you think are transferrable?

A: Yes

Q: Are these all the diseases?

A: Yes

Q: Does everyone agree that these diseases are transferrable form livestock to human beings?

A: Yes

Q: Could you describe the symptoms exhibited by an animal suffering from these diseases exhibits? For example, an animal suffering from orkipei, what are the symptoms the animal that can be seen?

A: Coughing, sometimes diarrhea, breathing heavily that leads to difficulty in breathing, mucus.it loses weight after some time. The animal may die in case it doesn’t receive help.

Q: And the next one?

A: Peeling of the hooves, difficulty in walking, it can be also observed through drinking of its milk, the milk causes a flu, foul smell even in the mouth

Q: Is there any other symptoms or we proceed to the next?

A: Lumps can be visible on the meat, red skin, it will also die if not treated

Q: Symptoms of the next disease? How does this disease affect the cattle?

A: When you eat the meat from the affected cow it causes diarrhea

Q: Are there any visible signs on the meat?

A: Blood from both ears and mouth. The animal’s stool will also have blood stains in it. Blood and mucus are the main signs. There are also swelling on one side, for example on the leg. Furthermore, there is clotting of blood.

Q: What human actions can lead to transmission of animal diseases?

A: Drinking milk, eating meat from the affected cattle.

Q: Are these the only ways in which animal diseases can affect human being?

A: Yes

Q: What about cooked meat?

A: Cooked meat is okay. The meat is better roasted, however boiled meat is better than roasted meat.

Q: We are in agreement when we say that these are the diseases that can be transmitted from livestock to human beings and they can be transmitted through drinking milk or eating meat from the affected cattle, right?

A: Yes

Q: What signs will I have if I get a disease from cattle?

Q: In humans?

A: Yes, in humans

A: You can get a headache and coughing. If not treated early it develops to Tuberculosis.

Q: Any other different opinions?

A: The first one, Orkipei, Cattle that have Orkipei cause a flu. It causes pneumonia in children. When you go to the hospital, it is discovered that Orkipei is in the area

Engororo, when you eat meat from a cow with this disease, you will experience stomachaches, diarrhea or pain in the lungs

Oloirobi can cause flu in a whole village and also causes cracking of the mouth.

Q: So when Oloirobi is present, there is flu?

A: Yes

Q: I would like to know different seasons with the type of diseases are most common. For example, dry season. Let’s start with the rainy season, which months do you experience rain?

Mid October, November and December.

Q: So the rainy season starts in mid-October?

Yes, 15^th^ of October

Q: What are the diseases common during this season?

A: Oloirobi, every time.

Q: When is the dry season?

A: Between mid-December to around mid-January thereafter we experience long rains.

Q: Which is the driest season in the area?

A: July to September. However, there are two rainy seasons. From March to May. These are the long rains.

Q: What diseases are common during this season?

A: Olerobi

Q We have discussed about diseases that can be transmitted from animals to human beings, ways in which they can be transmitted; through drinking milk and eating beef and the seasons that these diseases are mostly common. What risky actions can lead to getting these diseases apart from drinking milk and eating meat from affected cattle.

A: Being close to them when they sneeze or going into the cowshed.

Q: Is there any other way?

Silence

Q: Do you help your livestock when giving birth? Let’s say a cow or a goat?

A: Yes, every time they do it is necessary that you help them.

Q: Do you think that can cause transmission of diseases?

A: No, it doesn’t.

Q: Normally, who looks after the cows, goats and sheep in the community?

A: They are often mixed then one person goes to herd them. Initially children used to herd them but nowadays we employ the moran.

Q: So there is no division of duties such as women looking after one type of livestock?

A: No but the women take care of the young livestock. However, there are lambs that are lambs that have grown a little bit then they are taken care of by boys. Cows and goats can be looked after by boys or people who have been employed.

Q: Who milks the livestock?

A: Women do.

Q: In case you get any of these diseases, where is the first place that you can get help?

A: Dispensary. Nevertheless, there are many ways, before you go to the hospital you can get medicine from a shop first. Sometimes, we treat them by ourselves

Q: So are there organic medicines?

A: Yes

Q: You also buy from chemists?

A: Yes

Q: How will you know the type of medicine you are going to buy?

A: You go to the shop attendant and describe to them how you feel then he/she advices you on the type of medicine to buy. You are your own doctor though; one can use herbs. When you have a flu you take flu medication.

Q: When do you decide you need a doctor?

A: It depends; you are your own doctor. Some people use herbs until they get well.

Q: So other people use herbs while some go to the hospital and others will go to the chemists?

A: Yes

Q: I would like us to list some of the diseases that can cause death from the ones we’ve discussed.

A: In an order, tell me the diseases that need to be prioritized. Oltikana and orkipei. Every year the cattle are affected by these diseases.

Q: I’d like us to list five diseases that are transmitted from animals to people, which ones are to be prioritized?

A: Olerobi, Engororo, Nkuruno

Q: Why have the first disease be prioritized?

A: Because it affects the head. When the disease affects a goat, it doesn’t have direction in every homestead, there is always at least one livestock that is affected. The only treatment is to sell or slaughter the affected animal. It doesn’t have treatment so it has become a huge challenge to everyone.

Q: Why have you prioritized the second disease over the other one.

A: This is because it is very common. In every homestead there is livestock affected by it and there is no medication for this disease. Even though there is medicine, no one is able to help their neighbors with the. The vaccine is not easily found. We can go up to three years without the vaccine. We have not found a way to help ourselves with this disease. The responsibility therefore rests on an individual to sort himself out. Only one out of ten people can be able to help themselves out. Even if you decide to sort yourself the medicine is very expensive to buy.

Q: How about these that can affect human beings?

Why have you decided to make Olerobi priority?

A: This is because it has been common every year, even now, livestock are at home with a strong flu. The disease spreads easily from animals to human beings and this spreads easily in human beings, like a whole family can be affected by the flu. When you go to the hospitals they are flooded because of the outbreak. This disease has been frequent.

Q; We’ve discussed on places you can get help in case you get the disease. You’ve said Oloirobi is common, however, in the last six months to one year period, do you know of anyone who has been affected by these other diseases?

A: Yes, Engororo. This one causes anthrax or tropical cancer. I’ve heard a person stalking about tropical cancer. It is as a result of eating meat that has been infected by Engororo. It is not a common disease.

Q: So there should be help in dealing with this diseases?

A: Yes

Q: Which diseases would you like to be prioritized and why?

A: Orkipei, if possible number 3 also. We need a vaccine.

Q: Why?

A: Because it is expensive and it kills a lot of livestock. Oloirobi is easily transmitted to human beings however if doesn’t cause death.

Q: Death on human beings or livestock?

A: Livestock but orkipei is experienced throughout the year and causes death in livestock mostly ademycin helps in disease number one, however, we’ve tried ademycin and Penicillin, which have not worked in this case.

Q: It doesn’t have a cure?

A: No, the solution is slaughtering the animal.

Q: What can you do to prevent livestock from getting these diseases? Diseases that we have listed first.

A: Limit dipping the livestock because most of these diseases are airborne diseases. To avoid spreading of orkipei, we restrict movement of the affected livestock to other areas.

Q: Who is in charge of enforcing these restrictions?

A: There is a grazing committee.

Q: How do you know one has this disease?

A: It can be felt. Even when you got to the market, you only touch to know.

Q: What about number 7? We restrict movement.

A: Are there any other opinions?

Q: What about vaccination?

A: That applies to oloirobi. There are times that the government aid us with the vaccine but not nowadays you call the doctor. A doctor advised me to administer the medication in July to prevent Oloirobi. The vaccine is to be administered twice in a year after every six months.

Q: Who caters for the payment of the vaccine?

A: Initially we had prioritized Orkipei because we need vaccines and it is expensive to the livestock keepers even though few people try, it is very expensive.

Q: We will conclude here. We now know of the diseases to prioritize and the medication you also prioritize. Thank you for your time. Does anyone have a question? Thank you very much
