# Supplementary material for: Prioritization of livestock diseases by pastoralists in Oloitoktok Sub County, Kajiado County, Kenya
Source: PLoS One. 2023 Jul 12;18(7):e0287456. doi: 10.1371/journal.pone.0287456 (PMC10337939; doi:10.1371/journal.pone.0287456)
Supplement: S1 Data — (ZIP) [file pone.0287456.s001.zip › Oloitoktok transciptions/Transcripts Oloitoktok H/KII L.docx]

# KII

Q: You can start by telling us your name

A:

Q: What is the name of this dispensary?

A: Amboseli dispensary.

Q: What sub-county is it located?

A:

Q: How many villages does it cover?

A: It covers many villages though they are scattered. As you have seen while coming, from the gate some villages are seen up to the hill and about 10 kilometers from the border there are other villages that we serve.

Q: Does this mean it is a very big area?

A: Yes, it is but scattered.

Q: Is this the nearest dispensary someone can come to whenever there is a case?

A: We have two hospitals right now in Musethia the other one in Ngongarok. Ngongarok is about 15 kilometers away from here.

Q: How long have you been in charge in this area?

A: I have been here since 2014. This is the 7^th^ year.

Q: What is your highest level of education?
A: I am a an enrolled community nurse.

Q: Can you tell us about your role?

A: As you can see we have many roles here, one is with the outpatient patients. Outpatient those who are sick, we see them, cure and we dispense them with drugs. We also see the under-five; the ones we usually check for their weight and immunization. We also do deliveries though they are few because these people are transforming and actually making use of the hospitals. Previously, they used to use the TBE’s there in their manyattas but we are trying to make them know the importance of hospitals for deliveries. Although we have few deliveries, we are still going on with that process.

Q: So how are you trying to achieve that? Are you going to educate or give any materials? How are you trying to change that?
A: Previously I was alone, but of late, we have someone who follows up on them in the village. Perhaps you might have seen them outside. Enlightening their villages on coming for antenatal care here. In addition to that, to also embrace hospital deliveries. That is what we have been doing for a while now. Maybe in the next few years they shall catch up.

Q: What are some of the diseases you get in this area?

A: The common diseases are respiratory illness also skin and eye. As you have seen, the place is hot if you go to the village, you will see a lot of insects and flies. These flies lead to eye infections from one animal to another and the transmission is very high. The respiratory illness are the most common and plenty in number. In fact, it has been the order of the day. Therefore, our biggest problem is the respiratory diseases, the skin and the eye.

Q: Are you involved in any diseases surveillance or reporting of any kind?
A: This year no but we have been reporting. There is a form whereby, there are some diseases, which we usually report on every week. Such diseases are diarrhea, malaria, dysentery, if there is measles. However, I have not seen such lately.

Q: In the last how many months can you say this?

A: Three months. Although, we usually have malaria and dysentery.

Q: Does your facility get any cases of zoonotic diseases, diseases transmitted from animals to human beings whether domestic or wild?

A: Lately I have not seen any.

Q: When you say lately, which period do you mean?
A: We do have cases of rabies, though as an injury.

Q: Does this mean there has not been a case on that?

A: No, we usually just prevent them through these immunization but we have not seen any in this area.

Q: Do you immunize children against rabies?
A: No. Somebody bitten for maybe by a dog we usually give them, if we do not have, we send them for it.

Q: Do you have cases of people coming here bitten by dogs?
A: Not many, it is rare and we can stay up to 6 months or so.

Q: Does this mean that you have not handled any zoonotic cases in your facility?

A: No, unless animal-human conflict.

Q: What are some of the cases?

A: Cases like maybe somebody has been attacked by a buffalo or an elephant. These are common.

Q: How do you handle such cases?
A: Actually, we handle them according to their severity. Some if it is a cut we usually treat it as a cut. At times, we also involve the relevant authority, the KWS for them to take action. Some are so severe such that instead of even reporting here, they go straight to Oloitoktok where they are admitted. My work is just to report to them that have such cases and have referred them to Oloitoktok.

Q: How long have you been here?

A: This is my 7^th^ year.

Q: In this facility?

A: Yes.

Q: How would you describe the habits of the people in this area in terms of seeking medical help from health facilities? Is this something they easily do and do they directly come to the hospital when they fall sick?

A: Previously, getting 5 patients a day was difficult . When I inquired what was happening, I came to realize that they were using herbal medicines. Somebody might be having maybe diarrhea but opt to stay home and try self-medication with those herbal medicines. They will only come here for treatment only if the condition is severe. However, right now they have really embraced hospital treatment and usually come. I have not seen anyone resisting hospital treatment.

Q: In your opinion, what do you feel has changed that? Why do you think they are now embracing it?

A: I think this is because in the olden days, there were old men who could be seen in every place had knowledge on the herbs contributed unlike today they are very few who can be consulted. The other thing is, hospitals are cheaper and no money is required from them to get treatment. I think they have embraced this because of the government policy. Although they have been asked to be under NHIF, most of them have not done that yet. It actually does not cost them anything to come here and enjoy.

Q: Does this mean that the free medical services have helped?

A: They have helped a lot.

Q: In your opinion, do you think the hospital is the first place they come for help in case they feel unwell?
A: Right now, it is the first place they come.

Q: Is there any collaboration in terms of you in human health and in animal health in controlling diseases in this area in this Sub-county?

A: Yes there is, especially when it comes to water. There is a borehole shared by humans and animals, this is where many transmissions take place though not identified. However, when you try to get the source of maybe diarrhea and try to see what they are doing and how they get their daily water consumption, one can say there is some collaboration.

Q: Can you elaborate further on how you collaborate?

A: Using the same water where cows come and drink then women also come to fetch the same water, you see there is that contamination.

Q: Is there a time when the sub county veterinary will tell you there is an outbreak of this disease that can come to humans you should watch out for it or is there any kind of sharing of information between human health, animal health and maybe the KWS?
A: Right now no. However, we play our part by not only education them. In addition to that, like the one I told you about, she is trying to get involved by assisting them how they can construct or put a pipe there so women can get water apart from that water which is also used by animals. We have not actually talked to the vet since they are very rare here.

Q: Is there any reason for this?

A: You know here, it is a hospital their field area. There are some gates there with lights inside the park, where they try to keep their animals. You can only find them here only when the animals are sick.

Q: You said you have not reported any cases of zoonotic diseases in the time you have been here. Do you think they could be given a priority like you have talked about respiratory infection as one of your biggest challenge now and you have mentioned maybe someone bitten by a dog would come to seek for assistance? How do you rate them in terms of prioritization of the two, would you prioritize zoonotic disease and how would you do that?

A: I can say it is necessary since there was a time I was having cases on venom from snakebites. Although, you can stay here even some years without having such a case, it can be a priority because those cases are not easy. Snakebite or dog bite are severe cases and can be prioritized because one case can mean death.

Q: What steps would you take in case there is a zoonotic disease; have you ever heard cases of brucellosis in this area?

A: The problem we have here is that we do not have a lab. We have been planning with the sub-county how we can establish one because the catchment area is big. Apart from that, the other reason is, we usually send people to a facility called Namelok or Kimana I believe you have seen it on your way here. You see, direct diagnosis is hard without a lab though we have seen some referred here after being diagnosed and they take their medication here.

Q: Does this mean you are not able to say this is brucellosis?

A: Yes, because sometimes you cannot say this is a brucella using the signs and symptoms so we have to go further and do some lab investigations.

Q: Are the signs and symptoms common in your patients?

A: For example, this year I have found maybe two but there are cases that have been referred. Sometimes they are not indigenous of a place you can see somebody from Kimana or somewhere else coming to stay here. There was another time you see that the same person we shifted from here to Murtut. Few people are actually residents of this place since they have some relation between here in Namelok and Murtut. Somebody can be sent to Amboseli to go tell the one who is there to be giving injections because of that disease. This is why you cannot establish right away where they have acquired the disease, here or wherever they were.

Q: Do you still administer drugs from this place?
A: Yes, we administer.

Q: Are they several say in the last 6 months?
A: I have seen two in the last 6 months but yearly several maybe 3 or 4.

Q: Do you think it is a disease that should raise concern?

A: Yes.

Q: In terms of brucellosis and rabies, which one would you rank higher in your in terms of cases that you get?
A: The higher is brucellosis because I have seen it severally than others.

Q: Have you seen more cases of brucella?
A: Yes, though diagnosed from another facility, we have treated them here.

Q: This means it is not directly diagnosed here, correct?
A: I have not said that the disease is not here, it is only because of lab issues. We are the ones that sometimes send them to those facilities to be tested and others come already tested.

Q: As we finish, can you just give me your general view or perspective on zoonotic diseases in Oloitoktok and Amboseli at large?

A: As I have seen, those zoonotic diseases should be prioritized when it comes to finding the sources. I know some are even from our herds of cattle and domestic animals. In Oloitoktok and Amboseli at large, some people have met there death through these animals. I know some who were almost dying but they were taken care of so I can say zoonotic diseases should be taken as a priority in terms of taking care of our animals. Once animals are taken care of by our veterinary officers and us as a community, we are also involved by registering our domestic animals and taking care of them. It is important in order to cut the transmission of zoonotic diseases, though I have never thought about it earlier it is something new to me. This is why I have even left brucellosis, which sometimes may be common here. I think the government, WHO and the community can focus and collaborate to fight against both zoonotic diseases or cut the transmission of those diseases.
