# Supplementary material for: Prioritization of livestock diseases by pastoralists in Oloitoktok Sub County, Kajiado County, Kenya
Source: PLoS One. 2023 Jul 12;18(7):e0287456. doi: 10.1371/journal.pone.0287456 (PMC10337939; doi:10.1371/journal.pone.0287456)
Supplement: S1 Data — (ZIP) [file pone.0287456.s001.zip › Oloitoktok transciptions/Transcripts Oloitoktok H/IDI M 3.docx]

# IDI

Q: Kindly tell us your name.

A:

Q: What is the name of your ward?

A:

Q:And the village?

A:

Q: What is your age?

A: I am 38 years old.

Q: What is your education level?

A: I did not go far, I dropped out of school.

Q:What is your religion?

A: Pentecost

Q:For how long have you kept livestock?

A:I have kept them for long ,from the time I found my father with them when I was a child up to now.

Q: So your whole life you have been keeping them?

A: My whole life, that was my school.

Q:How did you start?

A: I started herding my father’s cows and then I got to get my own.

Q: How did you get yours? Did you buy them, were you gifted or you inherited from your father?

A: I was first given by my father, when you are taking care of your father’s animals you are given some among them, cows and goats and you take care of them until they increase in numbers I then got a job and bought mine but the ones that I started with I was given by my father.

Q: Why do you like keeping livestock and which ones?

A: I like keeping cows, goats and sheep because it has helped me up until this time. It is what feeds me ,when I sell them I can take my children to school at times we slaughter them ,that is why I love them.

Q: Can we therefore say you depend on them economically?

A: Yes my economy is dependent on my livestock.

Q:Of the livestcok you keep is there one that you prefer over the other?

A: I love all of them because they are mine, I love all of them because they all profit me.

Q:Where do you feed your livestock usually ?

A: I feed them around these hills.

Q:What is the place called?

A: Kyulu hills.

Q:What about during the dry season?

A:At the moment we still have grass so we just graze around here but when it is dry we migrate and go to Kyulu.

Q: So which months do you migrate to Kyulu?

A: From August it will be dry so will start following graze on the hills.

Q: Are there times when you get into Tsavo?

A: Yes at times we go into Tsavo when we don’t have grass.

Q: Is it easy?

A: No it is very hard and when you are caught it is bad.

Q: Are there times when you cross and go into Tanzania?

A: We have never gone to Tanzania.

Q: How about the pastoralist from Tanzania, do they cross into Kenya?

A: Yes they come at times.

Q: When you enter Tsavo, do your animals mix with wild animals?

A: Yes they mix even now here.

Q: They mix with which animals?

A: Zebras, Buffalos, giraffes and many of these small animals even lions.

Q: What are some of the challenges that you face as a pastoralist from this area?

A: The real challenge apart from diseases are the Lions because they attack our animals when grazing.

Q: When you talk of diseases which ones are you referring to?

A: We have diseases like Oloirobi,Engororo,Oltikana,Orkipey,there is one that came the other day that affects goats ,it affects the head and the goat keeps going round and if it is severe it paralyses the back and the goat cannot walk. This is what is disturbing goats and sheep but it does not infect the cows.

Q: What do you call it?

A: We call it Ormilo in Maasai.

Q: Is it common?

A: It is very common.

Q: Was it there in the past?

A: No it was not there in the past, we see it has come not too long ago. It used to affect the wildebeest as we here and that is how it has come to be transmitted to goats when it comes into contact with the fur from the wildebeest. It is because they mix with the goats and cows that’s how it has come to goats.

Q: The diseases that you have mentioned to me, do they come with seasons?

A: Yes, they come with seasons. The one called Oloirobi comes with season from March to April but lately it is there all the time. It would come during the rain and if it infected cows from say this Boma,it would infect animals in the whole area. It would come and go but now we keep seeing it.

Q: Would you say that there are some diseases found in some areas and not here for example from the hills or in Tsavo?

A:Yes,the one called Engoroto is mostly common in the hills because it is still brought by the wild animals.If it infects a cow it grows very thin ,when we are here we don’t experience that but when they migrate to the hill they get infected.

Q:And which disease is common in this area?

A: We still have Engoroto in this area but it is not common. Oloirobi is what is very common and Orkipei is also common affecting goat and sheep.

Q:How do you identify sick animals?

A: An animal cannot tell you they are sick so you have to know. You will see that the coat has changed you will know it is sick and you therefore inject it.

Q:When you say the coat has changed what do you mean?

A: In the case of Engoroto there are marks that you can see that will be indicative of the disease, it will have marks like ring worms and the skin will also appear different. The fur will shed.

Q: What is the first thing that you do when you notice such signs?

A: You will inject it with Terramycin before you know which disease it is and if it does not work you look for another drug.

Q: And the Terramycin do you buy or you have it in stock?

A: We usually buy and keep in the house if you see signs that your cow is sick you inject them.

Q: Did anyone teach you or you taught yourselves how to inject?

A: We just found it and we were taught by our parents.

Q: What will make you try and get alternative help?

A: If you inject it and see no change within three to four days you will then have to look for an alternative drug.

Q: Are there any traditional medicine that you use?

A: We don’t have any traditional medicine that we use for the diseases apart form one that we use when a cow has given birth and has not released the placenta so we can get the traditional medicine from the bush, boil it and give it to drink.

Q: Do we have government veterinary officers or doctors who help you?

A: No.It is long since I saw them.

Q: Do you know of diseases that can come from animals and infect people?

A: Yes, like the one I have mentioned Oloirobi it can come from animals and infect people because we drink that milk so when the animals have Oloirobi and we still use the milk and at times we also get infected and go to the hospital and get treated but it is the Oloirobi from cows.

Q: How does one know that they have been infected?

A: It is like a cold that moved from the animals and infects you.

Q: Apart from milk, is there another way that one can get infected by a disease from animals?

A: I do not know any other way.

Q: Of the diseases that you have mentioned to me, which one do you think should be given a higher priority?

A: The one called Engoroto because it really stays in the animal making it thing before it kills it and there is also one called Engororo that is very bad and finishes cows.

Q:Do you know if that can infect people?

A:I do not know.

Q: What can you do to protect your animals from getting sick and also not to infect you?

A: What is usually do because there is something that causes diseases so much when it bites a cow, Ticks. There are different ticks when it bites a cow it bring it those diseases because the animals go to the forest, you may find it has bitten a buffalo or wildebeest and when it bites the cow then it will give it disease so I wash my animals every week, every Saturday I make sure I have washed them and I feel that is better because it has reduced the diseases they are not as many because of washing them with deep.

Q: How did you know that you should be washing them, were you taught?

A:I just found out from the village, I was not taught .I just knew because we are with these animals.If you see your father doing it you also learn.

Q:Do you have any other way apart from washing them?

A:No.

Q:Do you know anyone who has been infected with a zoonotic disease in the recent past?

A:No.

Q:If by bad luck and animal gets sick and dies, what do you do?

A: There is nothing we can do, we just slaughter it and eat it.

Q: You don’t throw it away or burn it?

A: No, if you through it away it will be a curse, you cannot through away a whole cow like that. Although I am a Christian so I cannot eat something that has died but I am saying what happens because even here in our boma there are those that are not borne again but for me since I am borne again I will not eat something that is dead. But if we see it is about to die we cut the neck before it dies so much as it’s the disease that has killed it we will not throw it away.

Q: For those that say it’s a curse to throw away the animal what do they say would happen to you if you did?

A: You cannot throw away your full cow for the lion to eat, you better even slaughter it and give your children. We don’t really know we found it that way and we have followed.

Q: Apart from ticks that you have mentioned, do you know any other way that diseases can be transmitted to your livestock?

A: I don’t really know, you see even this Oloirobi I don’t know what cuts the cows hooves and I don’t know what causes that and at times it causes bruises on the tongue and makes the animal unable to eat grass so I don’t know what causes it.

Q:If by bad luck someone gets a zoonotic disease ,where is the first place that they would go for help?

A: I do not know because it has never happened.

Q;What else can you add on what we have talked about?

A: What I can add is if there can be help to prevent these diseases or educating people on diseases that can cause harm if people eat dead animals ,education is important and also if there is anything you can do to prevent the diseases from infecting the cows that would be great

Q:Thnak you very much for your time.
