# Supplementary material for: Prioritization of livestock diseases by pastoralists in Oloitoktok Sub County, Kajiado County, Kenya
Source: PLoS One. 2023 Jul 12;18(7):e0287456. doi: 10.1371/journal.pone.0287456 (PMC10337939; doi:10.1371/journal.pone.0287456)
Supplement: S1 Data — (ZIP) [file pone.0287456.s001.zip › Oloitoktok transciptions/Transcripts Oloitoktok H/FGD M 1.docx]

# FOCUS GROUP DISCUSSION

Q: As we start, which are some of the common diseases livestock diseases in this area, one of you will write them down for us?

A: Ormilo , Nunuk, Oloirobi, Engutikio oo changit, Orkipeii, olarashrash, kileny,oltikana, kububuo,engoroto.

Q: we shall add other as we continue if you remember for now you say this are the common ones

A: Yes

Q: If we take Oloirobi for instance, how you would know that your cow has contracted it?

A: The sign is that the animal will have pain in the mouth and feet, it will not be walking properly and will produce a lot of saliva from the mouth, the hooves will be cracked and it will not have strength to walk. It will have a lot of mucus

Q: So if you see these signs you will know it is Oloirobi

A: Yes

Q: Normally after seeing that, what is the first thing you will do?

A: We have medicine that we usually inject them with, we have adamycin or tylocin and this helps the animals.

Q: Do you do it yourselves or call veterinarians to help you inject the infected cow.

A: We do it ourselves.

Q: Where did you get this knowledge?

A: By observing the veterinary doctors and our parents do it.We used to see out parent do it and now we do it ourselves

Q: Basically, you try treating the disease yourself right?

A: Yes.

Q: Does everyone agree that oloirobi is a big problem in this area?

A: Yes, it is a very big problem.

Q: Can you explain how and why it is a problem?

A: It can cause a miscarriage in cows and this is the biggest effect of this disease.

Q: Apart from the miscarriages in cows, why do you also think the disease is a problem?

A: It weakens the cow a lot such that during drought, the cow will die quickly. It is also a challenge because it is ever present even in cold weather. It affects the legs of the cow making it difficult for it to walk. Sore mouth makes it hard for the cow to eat. In addition to that, it is easily transmitted from one cow to another. Low production of milk not only because the cow is unable to walk around and feed but also cannot eat because of the sore mouth. At times drinking water is also a problem because of the cracked lips and mouth.

Q: Is the disease present at all times or it has seasons?

A: It is ever present be it rain or drought.

Q: From the mentioned diseases, which one is more common in rainy seasons?

A: Nguruiyatanet is the most common during cold weather.

Q: Which effects does it have on the animal?

A: Sore eyes and can even cause blindness. They contract it from the wildebeest when the cows graze or drink water near the place the wildebeests have given birth and sad thing is we do not know the cure.

Q: Did you also experience this disease in the past this area?

A: Yes.

Q: What about engororo, what does it do to your animals?

A: It malnourishes the cow and rapidly weakens it. From minute 9:23- 9:37 not audible.

Q: Can it also kill the cow?

A: Yes, it can even kill the cow.

Q: What can you say about the olekipei disease?

A: This one affects the lungs especially in goats.

Q: How will you know when your goat has contracted it?

A: Excessive coughing.

Q: From the mentioned diseases, which ones can be transmitted from animals to human beings?

A: Oloirobi is the most common especially if you consume milk from an infected cow without boiling.

Q: Which other disease do you know?

A: Nunuk is transmitted by cows and another one is narrir.

Q: If you were to receive help from the government to help you deal with these diseases, which one would be your priority and why?

A: Elmilo because it has no cure compared to oloirobi that at least has a cure. Narrir is the second one as it brings loss to the owners since the cow has to be buried because it cannot be consumed.

Q: Which is the next priority?

A: Nguruiyatanet also has no cure and the options are to either slaughter or bury the animals.

Q: Is there anyone who thinks oloirobi should also be included in the list of priorities?

A: Yes.

Q: Why?

A: This is because it never ends hence, recurring.

Q: So far, you have only mentioned four, can you name two more?

A: Oleikipei is sort of the same like tuberculosis in human beings because of the excessive coughing. It kills many goats.

Q: The last one?

A: Kububu and kilen are almost similar. It is caused by ticks and makes the cow sleep where it is grazing and not come back home. This causes the cow to be attacked by the wild animals. Kububu mainly affects the cows whereas kilen attacks the goats.

Q: From the names of diseases you have given, is oloirobi the only one that can affect human beings too?

A: Narrir also affects human beings.

Q: Have you ever seen someone who has contracted narrir?

A: Yes.

Q: Can you explain further, how was the person, how were you able to tell it was the disease and which help did they receive?

A: One time, an infected cow died and when people consumed the meat, many were terribly ill.

Q: How were they helped?

A: They were taken to the hospital and others used Maasai traditional herbs which was effective and helped cure them.

Q: Is there someone else with a similar experience?

A: This disease is very dangerous since it can also kill someone.

Q: So what is the ideal way of dealing with an infected cow?

A: Burying so that even the dogs cannot eat the meat because they can also die from taking the infected meat.

Q: Has anyone seen or heard about someone who was infected with oloirobi, either in his homestead or village?

A: Yes, this is a common case and considering that the most common way in which it is transmitted is by consuming contaminated milk from an infected cow. Children are the first to contract it then adults.

Q: What is the first thing you do when you suspect either you or any of your children have been infected?

A: Go to the hospital for injections.

Q: Do you have any traditional methods for curing the disease?

A: We have them.

Q: At which point do you decide whether to go to the hospital or use the traditional methods?

A: If you see the first signs, you use the traditional herbs but when symptoms persist, you go to the hospital. Such symptoms are vomiting and diarrhoea.

Q: Does everyone agree with this notion?

A: Yes.

Q: Is there a different opinion?

A: No, because when you contract Oloirobi you vomit and diarrhoea.

Q : Can you also add on how these diseases can be transmitted from animals to people. He has mentioned milk and meat?

A : Well Oloirobi is like a flu, hence is communicable by air. If children play around infected animals, their cough transmits the infection from the children and spreads to the parents when they come back in the house. So air.

Q : Can interacting with animals be a source of diseases?

A : Yes, because we interact with them a lot.

Q : When do rainy seasons start around here and in what months?

A : Starts in October, ends in December and breaks for two months before starting again at around March to mid May.

Q : And at what time does Elnino start?

A : Elnino has no season. It is always there. And Narrir mostly comes during that rainy season. Especially when wild animals from Tanzania migrate into Kenya after it has rained.

Q : So wild animals bring the diseases?

A : Yes.

Q : What about Oloirobi?

A : Comes during the rainy season too.

Q : Before you had mentioned its always present?

A : No. It only rains twice in a year but it’s ever cold and between those periods Oloirobi affects us and that makes it seem like it’s always present.

Q : Olekipei?

A : All seasons.

Q : What about Kobubo and Kilen?

A : Always but depends on the area.

Q : When you are grazing your animals, how far do you go?

A : Maasai mara, Mombasa, everywhere.

Q : So for example, would you say that there is a disease in chul and not here?

A : Yes like Ongorotok, the disease that removes tail hairs is more present in chul because its mostly found in hilly places. And chul is a hilly place.

Q : And do you know what causes it?

A : Tse tse fly.

Q : Lets take Oloirobi for instance because it affects people, does it affect certain people more than others?

A : Yes mostly children. Because when a cow gives birth, the first milk is given to children who cannot wait for it to boil.

Q : So Oloirobi mostly affects children because they drink milk?

A : Yes.

Q : What do you do in prevention so that your livestock do not get infected?

A : Prevention is hard but when a disease arises in a certain area, we quarantine the animals from getting to other regions and spreading the disease. Sometimes we also vaccinate and wash with insecticides.

Q : When an area is affected, who makes sure the quarantined animals do not move from that area?

A : The elders in the community and veterinarians.

Q : Private veterinarians or government veterinarians?

A : Most are from the government but there are private veterinarians you can call.

Q : Government veterinarians vaccinate everyone’s animals?

A : Yes.

Q : The medicine you buy and use, how do you know it’s the right one for a disease?

A : For example Telocyn for Oleikipei. When you hear coughing from an animal, you inject it.

Q : And where did you learn that from?

A : From our fathers.

Q : How does your livestock falling ill impact you as a farmer?

A : That is our source of livelihood so it affects the whole family. It may cause stress, death and sometimes starvation.

Q : What disease among these would have a high impact in destroying your source of income by far?

A : Narrir, because you can’t sell or eat the affected animal.

Q : Do your domestic animals mix with the wild animals?

A : Yes.

Q : How is that relationship?

A : It’s very problematic because the wild animals cause diseases to our animals i.e Kobubo which is caused by ticks from herds of zebras. They also build up competition for pastures. Some wild animals like the lion kill our livestock.

Q : How is the relationship between Park rangers and cattle herders?

A: It’s very bad. They care more about the wild animals than our domestic animals.

Q : Is there anything you’d like to add to what we’ve discussed?

A : We are happy for your inquiry and we would like you to get us the medicine we lack for diseases like Elmilo and we’ll have been aided. We request that you all also do not take long to give us findings of your research. We would request to partner with the government that during times of famine, we may be allowed to graze on their parks.
