# Supplementary material for: Prioritization of livestock diseases by pastoralists in Oloitoktok Sub County, Kajiado County, Kenya
Source: PLoS One. 2023 Jul 12;18(7):e0287456. doi: 10.1371/journal.pone.0287456 (PMC10337939; doi:10.1371/journal.pone.0287456)
Supplement: S1 Data — (ZIP) [file pone.0287456.s001.zip › Oloitoktok transciptions/Transcripts Oloitoktok H/KII B.docx]

# KII

Q: As we start, kindly tell us your name

A:

Q: What is your designation?

A:

Q: How big is the village?

A: It is a big village and covers a large area.

Q: In which sub county is it?

A: It is in Kajiado.

Q: What is the name of the village?

A:

Q: How long have you been in that position?

A:I have stayed for long.

Q:How were you elected?

A:The neighbors look at the work you are doing then the see it is good work then they elect you.

Q:Are there many people elected?

A: They are many but every zone elects their leader.

Q: Have you worked for more than 10 or 20 years?

A:I have been there for some time and you know we don’t get paid for this ,it is about making sacrifice to work for people.

Q: What level of education do you have?

A: You know us we never went to school but we are knowledgeable and know that which is not good and that which is good and can make people live together in peace.

Q: Could you tell me what your job entails?

A: I do my work and when I see people having challenges I go and help them, I bring people together for them to leave in peace without fighting.

Q:For the period that you have been a leader ,what would you say are some of the challenges that pastoralists face in their daily life?

A: There are many diseases affecting the animals and we don’t get medicine. You know cows now are sick with this drought. Diseases have attacked the animals because of the drought.

Q: So most diseases come during the drought ?

A: During drought and when we get the green grass like now many animals get sick because the blood is flowing and the diseases that are dormant in the body come out.

Q: Do you have different seasons?

A: Yes we have different seasons.

Q: How many season do you have in a year?

A: We have three. In December it’s a good season for our livestock, during drought it is bad for animals because they die from starvation. June is the cold season and it enters the cows like pneumonia.

Q: So each season comes with its challenges.

A: Yes each season has its problems.

Q: Since you know that each season has its challenges are there measures that you have put in place to counter them?

A:At times the county can bring vaccines that help control the diseases ,but the diseases have become so many.We have a disease called Orkipei ,another one called Nahrii and another one that infects the cows like Pneumonia and the animal cannot stand. We just inject it with medicine.

Q: How does Orkipei affect the animal?

A: It makes the animal cough.

Q: Do you know what causes it?

A: It’s the sun, when the sun is too hot we have Orkipei.

Q: So the biggest challenge is diseases?

A: Yes.

Q: How about pasture?

A: At times it is a problem, when it has not rained then it becomes a big problem because there is no grass and the animals suffer so much

Q: Do you graze all the animals together that is cows, sheep and goats?

A: We graze them together but we separate them, we can have cows one side sheep and goats another side. We do management plan. Put goats here and sheep here.

Q: In your village, how far do you go when looking for pasture?

A: We go far.

Q:Are there times when you cross into Tanzania?

A:Yes ,at times we cross to Tanzania ,at times we go to Kyulu hills at times we go to Rombo.We go where it has rained .

Q:Are there times when your animals mix with the wild animals?

A:At times they mix with wild animals because we have a conservancy and in the conservancy it’s like a park so the animals mix with the wild ones, they graze together although they bring ticks, they eat grass together but if they drink stagnant water that is when they get sick .

Q:You have mentioned diseases as a challenge ,please tell me the diseases that give you problems.

A: There is a disease now called Keolunguny It infects the head and there is no cure.

Q: What does it do to the animals?

A: It enters the goat and it turn mad.When it infects a goat it can start running and it might run and disappear. The goat can appear drunk and even when it is injected it does not recover. There is no drug.

Q:Do you know what causes it?

A:We do not know the cause. We have even brought doctors who have taken blood samples and have said there is no cure for the disease and it is the disease that is finishing goats.

Q: When did you last have a serious disease outbreak?

A: The diseases are always with us ,there is no month that you can say there are no diseases, they are always there. We see as if it is airborne because there is no time when the diseases are not there.

Q: How do you identify sick animals?

A:It will have a rough coat , some will cough and some will even be shivering and some will have rough fur that way we will know that the animal is sick.

Q:Can you then know what disease it is?

A:Yes I can know if it is Oloirobi it will produce saliva from the mouth.If it is Oltikana it will have bloody urine ,if it is Orkipei the animal will be breathing heavily. If it is that disease for the head the goat will be running and falling and it can stay for a week without walking.

Q:When you see such sign in your animal what do you do?

A:You go to the shop and buy medicine .Right now we have many shops that sell medicine so you just go and say my goat has orkipei and they will give you the medicine for orkipei or if its oloirobi they give you penicillin .So that is what we do ,when the animal is sick you buy medicine and come and treat your animal.

Q:Do you use any traditional medicine?

A:For cows now we don’t have or for a cow that has given birth but has not released the placenta we have a tree called Ormumunyi that we boil the back and give it, this helps remove the placenta.

Q:Do you know of any disease that can come from animals and infect people?

A:Yes,Oloirobi if you drink milk that is not boiled you will get it.

Q: Do you know any other?

A: The other one is Nahrrii. If you eat an infected animal you will also get the disease.

Q:Where do people get help from incase of infection with Oloirobi?

A:You just go to hospital and you get medicine.

Q:So you have said you can get infected when you eat meat or drink milk?

A: Yes, although in the old days we used to drink milk that is not boiled and we never got sick. We would even drink dirty water in the bush and not get sick. You know now we have done some little research ,someone who looks after the animals like a young man who migrates with them never gets sick unlike the ones who stay at home .The ones that go with the animals even when they drink dirty water nothing happens to them and they eve drink milk without boiling and you know there is a new problem that has come because of the drought ,cows have started eating tomatoes, where tomatoes have been removed and the tomatoes were sprayed with chemicals which can enter the cow and cause harm and people will also drink milk form that animal and it is what you see bringing a lot of problems. That is what think is the problem.

Q: What do you think can be done to control spread of diseases and secondly for the farmers not to get diseases from their animals?

A: Maybe the government gets good medicine that can protect the animals and prevent them from infecting people because we eat meat from our animals because that is our wealth .I don’t know if the government has done research on vaccines that the will be giving our animals because I have seen the government in the past about two years ago they brought vaccines for cows and goats and then after the vaccination the animals never got sick, diseases reduced and that time because all the animals were vaccinated and I see it helped us at that time.

Q:So vaccination is important?

A: Vaccination is very important for the animals.

Q:What about disease infecting humans from livestock ?

A:You know we can’t know what the problem is there because we have cows, we drink milk ,we eat meat and that is our food ,we don’t know it is God who know what will happen because I think it is the air that has brought many diseases

Q:As we finish you have told me disease from milk and some causing wounds and the other diseases that give you problems ,which one would you give the highest priority ?

A: The one I would give priority is the one in goats called Kileny The one that infect the goat’s brain. That disease has no cure and it gives us so many problems. If your goats give you 50 kids you will only end up maybe with 5 or 6 that don’t get the disease the rest get it.

Q: When the animal dies what do you do?

A: You know right now people have joined churches, a goat might die and no one eats it because they are born again but other people will eat it, they boil and eat it.

Q: Would you says it’s because of religious teachings?

A: You know it is religious teaching that brought this thing that when an animal dies you can’t eat it because someone who is borne again cannot eat something that has died.

Q: So do you burry it or leave it there?

A: We just leave it there.

Q: As we conclude what can you add?

A: You know at the moment diseases have increased. We have all manner of diseases we have named them until we are now tired because diseases have become so many in people and in animals and we cannot say it is because we are drinking milk or eating meat ,I will just say it is the times. Diseases are so many now compare to the old days.

Q: Thank you very much for your time that is all I had.
