# Supplementary material for: Prioritization of livestock diseases by pastoralists in Oloitoktok Sub County, Kajiado County, Kenya
Source: PLoS One. 2023 Jul 12;18(7):e0287456. doi: 10.1371/journal.pone.0287456 (PMC10337939; doi:10.1371/journal.pone.0287456)
Supplement: S1 Data — (ZIP) [file pone.0287456.s001.zip › Oloitoktok transciptions/Transcripts Oloitoktok H/IDI M 2.docx]

# IDI

Q: What is your name?

A:

Q: What is the name of your ward?

A:

Q: What is the name of your village?

A:

Q: How old are you?

A: I am 52 years old.

Q: What is your highest level of education?

A: I never went to school, I have only saw the roof of the school but never got inside.

Q: What is your religion?

A: Christianity.

Q: For how long have you been keeping your animals, if you can remember?

A: Since I was born until now, I am a full pastoralist.

Q: How did you acquire your animals, did you buy, inherit from your father or gifted?

A: I inherited from my father though he is still alive. He gave me the small cows and since I am the firstborn in the homestead, I looked after his cows. Although they have become many, it is only right to say I got them from my father. However, I am now in my own home and my father in his.

Q: Do you only keep specific animals like only cows, sheep or goats only?

A: I keep all of them.

Q: How important are these animals to you?

A: They are very important to me because there is nothing else that I. My business is cows .I sell my cows to take my children to school, everything I do I depend on my cows nothing else. Cows and goats. If I want to buy a piece of land I sell my cows or goats and use the money to pay, I do not have any other business

Q: So we can say that animals are important to you?

A: Yes, very important especially cows. I wonder why the government has not set up a good project.

Q: For which animals?

A: For cows. Even now if I want one million shillings I can get it from selling cows in this boma.I have seen keeping livestock is very good.

Q: Where do you normally graze your animals ?

A: I feed them here, the mountains, everywhere so long as there is rain I go there.

Q: Is there different place you have secluded as a village designated for feeding or you just graze your cows anywhere?

A: Yes, there is though in July, I go near the park and during the rainy season, I go to the hills.

Q: What is the name of the hill?

A: Chyulu hills. I take my cows and goats there until December. Later on, I shift them down the slope where it rains and then move to Lang’ata a place called Katetema. It is a very good place for grazing your cows especially when it is raining. I even noticed the grass that side is better compared to the ones here. However, there are months where the cows refuse grazing on that side and actually prefer the ones here though now the cows have changed and want to graze on the hills.

Q: How do you know which side the cows prefer grazing?

A: You see where the animal is facing now, It faces the direction it wants to go graze on as now it is facing that direction.

Q: Do you determine this by looking at the direction the cow is facing?

A: Yes and it will refuse to go to the place it does not want to graze.

Q: Does this mean that most of the time the cow shows you the direction it wants to go?

A: Yes, even now if you take it where it does not want to go it will start mooing and make a lot of noise until you return it or pass where it wants to be, it remains silent. However, I usually know exactly where the cow wants to go.

Q: You mentioned there are times that you go near the park, which park is that?

A: Chyulu national park since we border both Chyulu and Tsavo west national park. See those hills, though distinct, there is a park and a boundary between Taita and Kajiado County. This side of the hills is Chyulu national park bordering Kibwezi.

Q: When you go near the park, is there a time you actually enter?

A: Yes, especially when it rains and we see grass in the park.

Q: Does this mean that you actually enter the park?

A: We do though not often. .

Q: Is doing this legal?

A: There are just people who protect the park and we still enter.

Q: When you do this, do you interact with the wild animals?

A: Yes, even here in the community.Infact the wild animals are more here than in the park. When it reaches 7:00pm, zebras come up to this point and mix with our cows.

Q: Have you ever gone to Tanzania to look for pasture?

A: No. I have not gone to Tanzania but I have entered Tsavo. I do not go to Tanzania.

Q: What are some of the challenges you face and risks you go through in search of pasture?

A: Drought, diseases affecting the cows especially the mouth region and it is deadly. Even now, cows are not that healthy as they are still recovering from that disease. It has been about for four years and the government has still not vaccinated them against such diseases. The foot and mouth disease has been a problem in cows. Ndorobo is in plenty in this area but when you cross that river, the diseases is not alarming compared to this side. I am not sure which grass is affecting these cows the most though Ndorobo is not that big of a problem since there is a drug to cure the disease. The disease that brings loss in this area is the one affecting the mouth. In sheep and goats, I am not sure about the name of the name because it has started affecting recently. It is deadly since there is no cure for it and the only solution is to put the animal down.

Q: What does it do to the animals?

A: It mostly affects sheep and goat in the head causing dizziness, Coenurosis(*Ormilo*) there is no cure yet although it does not kill the animals but it is a very big problem. Apart from drought, this disease and the mouth disease affecting cows are the ones challenging us the most in this area.

Q: Are there diseases you will find here but not in Chyulu or Tsavo when you take animals grazing?

A: Pastoralists know the different types of diseases in different areas and their cure too. They can easily differentiate the diseases and know which ones are transmitted from wild animals or grass. When they take their cows for dipping, they are immune to certain diseases. However, if they last up to three weeks without dipping the cows are heavily infested with ticks.

Q: Can you give me an example of a disease that can be transmitted from wild animals even if it is in Maasai?

A: He is saying there are diseases like enterotoxaemia, ECF and black water .

Q: Are these the diseases transmitted from wild animals?

A: Yes, from the wild animals to our domestic animals.

Q: Are there seasonal diseases, the ones that outbreak in the rainy or drought season?

A: During the rainy season, the diseases are in rampant.

Q: What are some of these diseases?

A: ECF, Enterotoxaemia, Trypanosomiasis but this attacks all the time.

Q: Are there diseases found in certain areas and not in others?

A: Yes.

Q: Which are some of the areas?

A: For example, black water disease is a lot here in Intilal.

Q: What does the disease do to the animals?

A: In Chyulu hills, there is grass called Olepito and when cows feed on it, they get the trypanosomiasis (*engoroto*) disease. It is not that deadly since there is a cure for it.

Q: Does it only surface during the rainy season?

A: It strikes all the time, during drought and the rainy season.

Q: How can you tell your animal is unwell?

A: Yes I know how to tell. I can just tell by looking at the skin and fur.

Q: How will the fur look like?

A: The fur will be rough, it will have teary eyes. In others I observe the tail and see they are shedding an I will know that that is Trypanosomiasis (*Engoroto*).The Maasai know how to tell when their animals are sick

Q: When you see or suspect your animal is sick, what is the first thing you do?

A: I inject them.

Q: Would you be having those drugs?

A: Yes, especially the one for trypanosomiasis disease, I have that drug.

Q: Does this mean you usually have medicine at home?

A: Yes but if there is one I have not heard of, I look for the drug and keep at home.

Q: Where do you get the medicine?

A: I buy them.

Q: Are these medicines easily accessible?

A: Yes, nowadays they are easily accessible.

Q: What is the normal price?

A: They have become very expensive.

Q: Are there other Maasai traditional methods you can use to treat your animals or do you normally buy medicine?

A: There is no other way you have to buy the medicine. However, when I see the condition of the animal has worsened, I call the veterinary doctor and he injects the animal.

Q: Is that a private veterinary doctor?

A: Yes.

Q: What about the county one?

A: No, there is no veterinary doctor from the county. This makes me wonder where they take these medicines.

Q: So can we say that when you see your animal is sick, you buy medicine?

A: Yes but when see the number of sick animals is large, I call the veterinary. The only disease currently stressing me out is the foot and mouth disease because the medication is very expensive. For instance, when you go to the county, the medicine is Ksh. 9,000, which is hard for the owners of the affected cows since the disease does not only affect one cow but as many as 200 cows at once. The other diseases are easier to manage since they only affect one cow at a time. The foot and mouth disease affects all cows and we have not yet received any assistance. This is why I usually call the chief and councilor in order for them to call the county to come and administer the oloirobi vaccine that is very hard to find.

Q: Where and how did you learn about injecting your animals when sick?

A: No one taught me how to administer the injections.

Q: Does this mean that you taught yourself?

A: Yes, I taught myself. We even call the veterinarians and tell them to bring the drugs at home.

Q: After they are brought, do you still inject them yourselves?

A: Yes, I inject them by myself.

Q: You said that there are no veterinarians from the county who walk around offering their services, is that correct?

A: Yes, there are no veterinarians from the county. However, there is only one man called Gitonga who walks around injecting cows.

Q: Does he always walks around or comes when he is called?

A: When you need him, you call him and he actually comes. For instance, if I call him right now and ask him to come tomorrow he will come.

Q: Do you know any diseases transmitted by animals to human beings?

A: Yes, there is one disease called FMD ( *oloirobi)*. When cows get this disease, they also infect humans with it.

Q: What does this *oloirobi* do to the cow?

A: It usually affects the legs and mouth making it difficult to eat. The disease is dangerous especially to the young calf. When the cows contract FMD, the Maasai use fire to tattoo the cow with the letter X.

Q: Does this help in any way?

A: We believe it helps.

Q: You tattoo with the fire the letter X?

A: Yes, with fire.

Q: Where?

A: On the stomach.

Q: Does it work?

A: Yes.

Q: What do you do when it affects humans?

A: We just go to the hospital for treatment.

Q: How do you know you have contracted the disease?

A: You will have symptoms almost similar to the one a cow has when it has contracted the disease like sore mouth.

Q: Apart from FMD, do you know any other disease transmitted from animals to human beings?

A: Yes, there is another one called anthrax. It usually attacks cows and when you eat meat that is not well cooked, you can get it and it is the most dangerous disease in human beings. It kills cows and you will find even village elders telling people to ensure they cook their meat well because this disease is very dangerous.

Q: Do you get this disease when you eat meat that is not properly cooked?

A: Yes, you can easily contract the disease.

Q: Do you know any disease you can get from consuming milk?

A: That one is oloirobi and when the cow has this disease, you should not drink the milk until it completely heals. However, if it is angoroto you can still milk the cow.

Q: Can you say the most harmful one is anthrax?

A: Yes, though the most dangerous one in this area is oloirobi. Nonetheless, it does not kill cows though it has affected many cows especially in May. Nowadays, it affects the cows monthly such that there are times you can find it has immobilized the cow. In the past three years, the disease has been in rampant. There are times we get vaccines and others we do not.

Q: Does the government bring vaccines?

A: Yes, they bring every year.

Q: Are the vaccines free or you pay?

A: The vaccines are free when they see the animals are many though we give the doctor a goat as a token.

Q: Can you say that one way of preventing these diseases is through vaccination?

A: Yes.

Q: What is another way you can use to prevent these diseases from attacking your animals?

A: I do not know any other way because people have become many. Others go to Kimana and can clearly see animals from that side are infected they pass here every Friday from Tanzania. Not a day passes without a cow being infected with oloirobi. I do not know if you have seen but the cows do not completely heal from oloirobi. I just look for the vaccine and administer because this movement spreads the disease a lot since the animals interact with the affected ones. My cows also get infected at the market when someone else’s cows have oloirobi and they interact with mine. I hear the disease is in Matabato yet it reaches here because of the movement and interactions between the animals.

Q: Can you say that when the animals interact with infected ones they can get the disease?

A: Yes. When cows drink from running water, it is hard for them to contract the disease unlike from boreholes where the water is stagnant.

Q: When they come to vaccinate the cows, do they call you to educate you on the prevention of these diseases?

A: No. They call the chief for him to inform us that on a certain day the veterinarian officers will be around to vaccinate the animals however, this used to happen long ago. That time the chief would bring the veterinarians but it has been over four years now.

Q: In the past 6 months or one year, have you seen or heard cases of these diseases transmitted from animals to human beings? You mentioned oloirobi is common, is it currently in the area?

A: Yes, it is in plenty. For example, that bull over there has oloirobi. It is still healing though sometimes you can find about a hundred cows sleeping because they are unable to walk.

Q: Do you call the veterinary when this happens?

A: Yes.

Q: What do you when the animals die?

A: We eat.

Q: Do you still eat the meat regardless of the disease that killed the animal?

A: Yes, even if it was a snakebite.

Q: Do you cook the meat or just eat it that way.

A: We cook the meat no one eats it that way apart from the lions.

Q: Do you eat the meat of an animal killed by a disease?

A: Yes.

Q: Do you know what causes FMD?

A: No. I only see the infected cows but I have no idea what causes it.

Q: In your family or this village, has someone ever contracted any disease from animals, from eating not well-cooked meat and what happened?

A: Yes, I have heard cases of people dying after eating meat from a cow and sheep.

Q: Which disease is the most dangerous to the wild animals?

A: Anthrax, though it does not kill them.

Q: In your own opinion, which animal disease affecting the people in this area do you think the government should prioritize?

A: FMD.

Q: Why?

A: This is because the medicine are expensive and hard to find. I was even looking for it the other day and was unable to find it. I have 200 cows and when I go to the veterinary, they ask for either Ksh. 80,000 or Ksh. 100,000 where do I get such an amount? I would like the government to try helping us with that since this disease affects all the cows unlike the other diseases. The oloirobi medicine is very hard to find, it is not found in shops and you have to go up to the national government county in order to get it.

Q: As we finish, where do you think one should get help from when they have contracted any disease from animals, using traditional herbs or go to the hospital?

A: I have never seen any traditional herbs that cure the animals. At times, one might try to give the cow herbs but they do not help in any way whatsoever.

Q: Do you mean to say that these herbs do not help?

A: No, they do not work.

Q: What can you add on the conversation we have had?

A: I just want to ask for help concerning the oloirobi medicine because it is the most dangerous disease-affecting cows. In addition to that, the dip too.

Q: You mentioned that you are given vaccinations, what about the dip?

A: No.

Q: Do you buy them?

A: Yes.

Q: Do you think using the dip is one of the ways to prevent the transmission of these diseases?

A: Yes, it prevents those other diseases especially in oltotikna and anthrax, dipping is the best. This helps get rid of the ticks in these animals. Unfortunately, it is not effective in FMDi.

Q: Do you have anything else you might want to add?

A: No.
