# Supplementary material for: Prioritization of livestock diseases by pastoralists in Oloitoktok Sub County, Kajiado County, Kenya
Source: PLoS One. 2023 Jul 12;18(7):e0287456. doi: 10.1371/journal.pone.0287456 (PMC10337939; doi:10.1371/journal.pone.0287456)
Supplement: S1 Data — (ZIP) [file pone.0287456.s001.zip › Oloitoktok transciptions/Transcripts Oloitoktok H/KII I.docx]

# KII

**Q:** What are your full names?

**A:**

**Q:** What is your designation?

**A:** I am a veterinarian working

**Q:** Which sub-county?

**A:**

**Q:** How long have you been doing this?

**A:** For about 15 years but I have been in this specific area for the past three years.

**Q:** What is your highest level of education?

**A:** I am a PHD student.

**Q:** What does your role entail?

**A:**It is more of Disease investigation, disease management and assisting other colleagues in other professions within the organization in matters that relate to veterinary issues.

**Q:** Do you specialize or focus on both domestic and wild animals?

**A:** We specialize mainly on the wild department but by the obligations of our profession, we do reporting for diseases across all species wild or domestic since diseases will not choose which group to attack. We report diseases within the area. However, my job description is on wildlife.

**Q:** Do you also interact with the Kenya Forest Services in your line of duty?

**A:** As a government parastatal, you deal with many officers from different sections of the government in both wildlife and forest services. Besides, forest service is a sister organization since we are in the same ministry and most parks have forests. Some of our parks have duo-gazettement because they are registered as both forests and as parks. We have a portion of the wildlife service and the forest service so we work together.

**Q:** Could you tell me about the interaction of pastoralists and wildlife in area your area that you cover?

**A:** The gazetted areas designated for parks is small compared to the animal home ranges which is extensive. This includes the pastoral areas that are around the park which are more of conservation areas that are not necessarily destroyed since they have habitats that are intact. Animals in the area have also adapted. There are also dispersal areas between ecosystems that have been gazetted as national parks. Therefore, there is heavy interaction between livestock and wildlife within this area; Amboseli and Tsavo ecosystem, where I work.

**Q:** What is animal home ranges as you have mentioned earlier?

**A:** An animal does not exist in a particular place like a human being would stay in a certain home. They would live and exist in an area that depend on different species and cover a big area. In the case of an elephant, they have a place where they would spend their nights and have a different place where they do their browsing and feeding. Scientifically, there are certain distances within which most animals will exist. There are some animals that are known to disperse in long distances whereas there are some that are restricted in an area.

**Q:** During these interactions, are there times where pastoralists enter the parks to graze?

**A:** There are certain government areas designated as wildlife areas but they are too small to hold the numbers that exist in the country. In fact, close to 80% of wildlife in the country live outside gazetted areas. It will be wrong to imagine that animals exist in the parks. They exist in natural habitats whereby pastoralists within this region have left most of the areas to be as natural as they were since the beginning of time so they are more suitable as breeding population of wildlife and existence of animals. This was so even in most areas of the country but when there was habitat destruction, maybe animals were bright enough to move to other areas. For now, most animals are still within the area because it is suitable and favourable for their existence. Therefore, this means that the wildlife does not exist in the parks alone but the almost the whole of Kajiado is a wildlife dispersal area.

**Q:** So does this mean that the pastoralists do not enter the park?

**A:** The Park is a very small entity in the ecosystem hence; even if pastoralists were to come, they would only spend a week before the grass is out because they have many livestock.It is god to note that at times of the year we have an understanding with the local community around the park and especially when it is very dry for them to bring on their animals to the watering area but specific areas and at specific times that is the agreement ,an MOU kind of and this is a way of trying to create co-existence between us the government and the community around us s. We have a population of about 1700 elephants in this area and I can tell you for sure these animals cannot have enough space in the park. They always come to the park to feed in the mashes then go to the villages in the evening. In order for the community to take care of them, we also take care of them when they need water. It is some sort of an understanding but there is no official agreement for anyone to come and graze in the park. It is not allowed. We however allow them to come for water occasionally.

**Q:** So can you say that it is seasonal?

**A:** Yes, it is only allowed during drought otherwise they have no other reason to come. It is also a big threat to them and a risk they are taking since we have lions and when they bring cows, they will be taken out.

**Q:** In your log experience, do you know of any diseases that can be transmitted by the wild animals to the domestic animals and later on to human beings?

**A:** Yes I do.

**Q:** Do you have any cases that have been reported in this area?

**A:** Not recently but there is always suspicion and the fact that we are not into very active surveillance in trying to establish these cases. Either way, every time we have rumours or indications, we take our tests but it is nothing very alarming despite the fact that we are always concerned and vigilant in case anything would come up.

**Q:** What are some of the indications that guide you?

**A:** For instance, rabies. This is because anytime a wild animal comes to any settlement area or are known to bite human beings or animals in the area; we always organize and take them to Kabete for rabies tests. Fortunately, we have not had any positive cases in the recent past that I have worked here although I am not sure about the long history of the place but that is one of the major concerns. However, we have not found any alarming cases. I think that before I came here around maybe 2007, 2008 or 2009, there was drought and suspicion of anthrax. Though I have never personally handled an anthrax case, I believe the main issue was drought. We never had human cases but had some suspects in wildlife but it was never confirmed so I would imagine there was no case. These are the indications we use; if there is any kind of concern that a disease would exist, we always do the testing. Testing is done mostly on need basis because if we did it actively it would be very costly and at times not easy to manage. We have not had any disease of concern as far as zoonosis is concerned but we are always alert because at times, the cycles would not be very visible. This however, does not mean there is no disease because there are chances that some people would get sick and rarely relate that to wild animals or the livestock they keep. Diseases would normally exist but they are not visible. An epidemic on the other hand, would be a major concern or a localised kind of attack

Q: What are some of the positive or negative effects you have noticed on the environment because of livestock keeping or any pastoralists’ activities in the area whether?

A: Some of the positive effects is that it is a way of life since it acts as an economy. Animals in the park would be on the negative side because they would mostly end up causing destruction of the habitat. One of the biggest problem that I have and it is a personal opinion is that the number of livestock in Kajiado south is more than the area holds. This causes a lot of habitat destruction in the area up to even Oloitoktok, there are no herbs or grass for animals to feed on although the area looks very green but there is nothing for livestock to feed on and I tend to think this is because of overgrazing. Ideally, I think animals and livestock exist in a good way around here but there are certain things like diseases though not zoonotic, affect the livestock like malignant catarrhal fever. However, this is not a problem for animals in the park but the areas around the park where there is livestock, the impact is negative. In addition to that, I am not sure there is a vaccine to malignant catarrhal fever but I think the endemic stability exists within the area though I am not sure how suitable it is to the local population.

Q: Is there collaboration between your department, the veterinary department and the ministry of health in managing such issues?

A: Yes, I think there is the zoonotic disease unit( ZDU ) where we have representation .We do disease reporting like any other veterinarian in the ministry because diseases will not really matter whether we are followed by a parastatal or any government organization. It is all under the government only difference is that we have different sections for speciality and not effectiveness of the management. There is collaboration and ideally as colleagues, we meet in different forums. We share an external programme on disease surveillance. We also experience challenges for example, the coronavirus has made meeting with colleagues a big problem.

Q: Do you have anything to add as we wrap this up?

A: I think diseases would exist and naturally before they erupt, they are somewhere within the ecosystem. Any disturbance like increase in wildlife population, increase in livestock because of the rise in economy. People are trying to be empowered for instance, if you had one cow you want to have two and imagine that you will get more income to sustain you. Nevertheless, nothing that can be done to the limited space. Changes in the habitat would also provoke other diseases that are not prevalent to come up. I think it is an issue of us being vigilant because there would always be diseases even if not seen because changes are continuous. We may have not gone up to the peaks where the diseases would erupt but maybe one time, a little change would overturn everything. Unfortunately, even in medical field I have never seen any doctors concerned about animals when you go to them. There are no epidemiological questions mostly but an imagination that any disease reported would be a human disease. Nobody investigates your human interaction with the animals. There is need for awareness because they know I am just not sure what they do about it.

Q: Is there a formal forum where you meet and talk about the one health approach ?

A: Yes, nowadays there are official forums. There are even common projects that are being undertaken in wildlife, livestock and human health. I think there are some standing committees that deal with such health approaches.

Q: Is there anything else?

A: No.
