# Supplementary material for: Prioritization of livestock diseases by pastoralists in Oloitoktok Sub County, Kajiado County, Kenya
Source: PLoS One. 2023 Jul 12;18(7):e0287456. doi: 10.1371/journal.pone.0287456 (PMC10337939; doi:10.1371/journal.pone.0287456)
Supplement: S1 Data — (ZIP) [file pone.0287456.s001.zip › Oloitoktok transciptions/Transcripts Oloitoktok H/IDI M 4.docx]

# IDI

Q: Good morning?

A:

Q: What is the name of your ward?

A:

Q: And the village?

A:

Q: How old are you?

A: 57 years.

Q: What is your education level?

A: I dropped out in class 2.

Q: What about your religion?

A: Good hope.

Q: How long have you kept livestock?

A: I have had livestock from a long time ago when our father was still here and livestock is a very good thing to the Maasai if it is not for the drought the Maasai can live very well with the animals because they are just like humans they use blood. We only have problems during drought but there is nothing that we can do because that is the work of God. So the livestock are very good to us even when a child is going to school you sale one and it is enough for all that work and it is what we mostly use in our life. If I go to Kimana I can sell one and bring food for the children because many Maasai don’t farm but now things are changing some practice farming.

Q: How did you start keeping livestock? Were you given by your father or you bought?

A: I was given by my father and he asked me to take care of them and also add mine.

Q: Which animals do you keep?

A: I keep sheep, goats, cows, donkeys and even chicken. They all have money and give us profit.

Q: So you can’t say that one is more important than the other?

A: They are all important because if I take this to the market it will help me solve the problem and if I take the cow to the market and the children are five I will close that issue.

Q: Where do you graze your animals?

A: This is my land and I also go to the conservancy but when the drought is too much ,like right now the animals are not here they go to Amboseli or even Lengesin where it has rained, we move them you know us we are people who migrate like animals and that is good for us because when we have drought here we don’t stay here I go to my brothers far and they will receive me and I will return when it rains.

Q: Where is that far place that you go to?

A:It is very far ,past Amboseli and it happens during the dry season.

Q: Which month does that happen?

A: Like this month, because it has not rained this year the way it should have, from March, April up to this time so the big animals have moved we are just left with this small ones.

Q: And which is the rainy season?

A: From December to January it’s the rainy season but it can start early is November.

Q: When you go to Amboseli, do your animals mix with the wild animals?

A: Yes they mix and at times the wild animals cause harm to ours but we have no otherwise because they are found in our land and they bring us benefit in terms of bursary.
